# Enantioselective Synthesis of Acyclic Orthogonally Functionalized Compounds Bearing a Quaternary Stereocenter Using Chiral Ammonium Salt Catalysis

**DOI:** 10.1002/open.202100162

**Published:** 2021-08-05

**Authors:** Katharina Röser, Bettina Berger, Michael Widhalm, Mario Waser

**Affiliations:** ^1^ Johannes Kepler University Linz Institute of Organic Chemistry Altenbergerstraße 69 4040 Linz Austria; ^2^ University of Vienna Institute of Chemical Catalysis Währinger Strasse 38 1090 Vienna Austria

**Keywords:** ammonium salt catalysis, asymmetric alkylation reactions, cyanoacetates, phase-transfer catalysis, quaternary stereogenic centers

## Abstract

We herein report an asymmetric protocol to access a series of orthogonally functionalized acyclic chiral target molecules containing a quaternary stereogenic center by carrying out the enantioselective α‐alkylation of novel orthogonally functionalized dioxolane‐containing cyanoacetates under chiral ammonium salt catalysis. By using just 1 mol % of Maruoka's spirocyclic ammonium salt catalysts enantioselectivities up to e.r.=97.5 : 2.5 could be achieved and further functional group manipulations of the products were carried out as well.

## Graphical abstract

**Highly functionalized** acyclic compounds bearing a quaternary stereocenter can be accessed by using a chiral ammonium salt‐catalyzed α‐alkylation protocol.

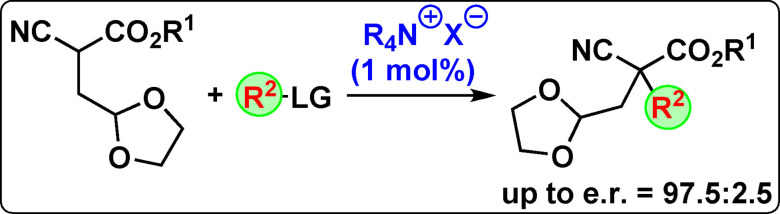

## 1 Introduction

**Scheme 1:**
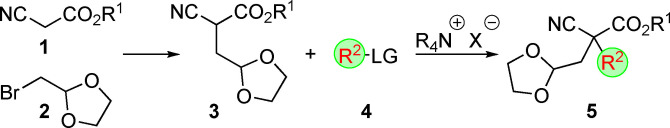
Investigated use of compounds **3** to access acyclic targets **5** under asymmetric ammonium salt catalysis.

The enantioselective construction of quaternary stereogenic centers, or so called all‐carbon stereocenters, has for decades been a topic of uttermost importance^[1]^ and the development of novel, especially asymmetric catalysis‐based, strategies is a worthwhile task.[^1^, ^2^, ^3^] Enantioselective catalytic α‐alkylation reactions of suited prochiral C‐pronucleophiles,^[4]^ i. e. enolate analogues, are amongst the most versatile approaches to install stereogenic centers and the use of appropriately α‐trisubstituted carbonyl‐ or carboxylic acid‐analogues allows for the direct formation of quaternary stereogenic centers.[^3^, ^4^] Asymmetric ion‐pairing organocatalysis, i. e. chiral ammonium salt catalysis, has emerged as one of the outstanding non‐covalent catalysis principles over the last four decades.^[5]^ Very remarkably, this concept has proven its potential for stereoselective enolate α‐functionalizations ever since the pioneering contributions by Merck scientists, who first reported the chiral ammonium salt catalyzed α‐alkylation of phenylindanone derivatives in 1984 already,^[6]^ and O'Donnell's group who introduced glycine Schiff base α‐alkylations in 1989.^[7]^ Following these seminal contributions, the use of chiral ammonium salts became a well‐described methodology for asymmetric α‐alkylations and over the years also various approaches for the construction of quaternary stereocenters by reacting cyclic[^6^, ^8^] as well as acyclic^[9]^ prochiral enolate analogues with Csp^3^‐alkylating agents (i. e. alkylhalides) have been introduced. Our groups have been engaged in the design and use of chiral ammonium salt catalysts for (novel) asymmetric α‐(hetero)functionalizations of prochiral C‐nucleophiles for a while.[^10^, ^11^] Based on the general high interest in acyclic compounds bearing a quaternary stereogenic center we now became interested in introducing the novel orthogonally functionalized α‐cyanoacetates **3** as a versatile substrate for (chiral ammonium salt‐catalyzed) asymmetric α‐alkylations.^[12]^ As outlined in Scheme 1, these starting materials should be accessible by alkylation of cyanoacetates **1** with the dioxolane‐based electrophile **2** and asymmetric α‐alkylations with Csp^3^‐based electrophiles **4** will give access to the highly functionalized chiral acyclic targets **5** then.

## 2 Results and Discussion

We started our investigations by focusing on the α‐benzylation of the *t*‐butyl ester **3 a**, which was accessed by alkylation of the corresponding cyanoacetate **1 a** with **2**,^[13]^ in the presence of the established chiral ammonium salts **A**,[^5^, ^6^, ^7^, ^14^] **B**,[^10b^, ^10c^] and **C**[^5^, ^15^] (Table 1 gives an overview about the most significant results obtained in a detailed screening of different conditions). First experiments with Cinchona alkaloid‐based ammonium salts **A1** and **A2** under classical biphasic phase‐transfer conditions (toluene / aqueous KOH) allowed for very clean reactions and gave product **5 a** in excellent yields after 2 h, but in a more or less racemic manner only (entries 1 and 2). Other Cinchona alkaloid‐based ammonium salts were tested under various conditions too, but no improvement was possible (details not given in the table). We next tested our own bifunctional ammonium salts **B**, but unfortunately these systems were not selective at all either (for a representative example see entry 3). Gratifyingly however, when using Maruoka's structurally more rigid spiro ammonium salt **C1** next, literally the first experiment with 5 mol % of this catalyst gave product **5 a** in a high isolated yield (87 %) and with good enantioselectivity (e.r.=94 : 6). Remarkably, when lowering the catalyst loading to 1 mol % only, the e.r. could even be improved to 96.5 : 3.5 (entry 5). Interestingly, catalyst **C2** performed notably less selective (entry 6) and we therefore screened a variety of different conditions with **C1** next (entries 7–13). Surprisingly however, neither the use of other solvents (entries 7 and 8), nor the use of other aqueous or solid bases (entries 9–11) did allow for any improvement. When lowering the temperature, the e.r. could not be improved further, but instead the reaction only slowed down significantly.

**Table 1:** Identification of the best‐suited conditions for the asymmetric synthesis of **5 a**.^[a]^

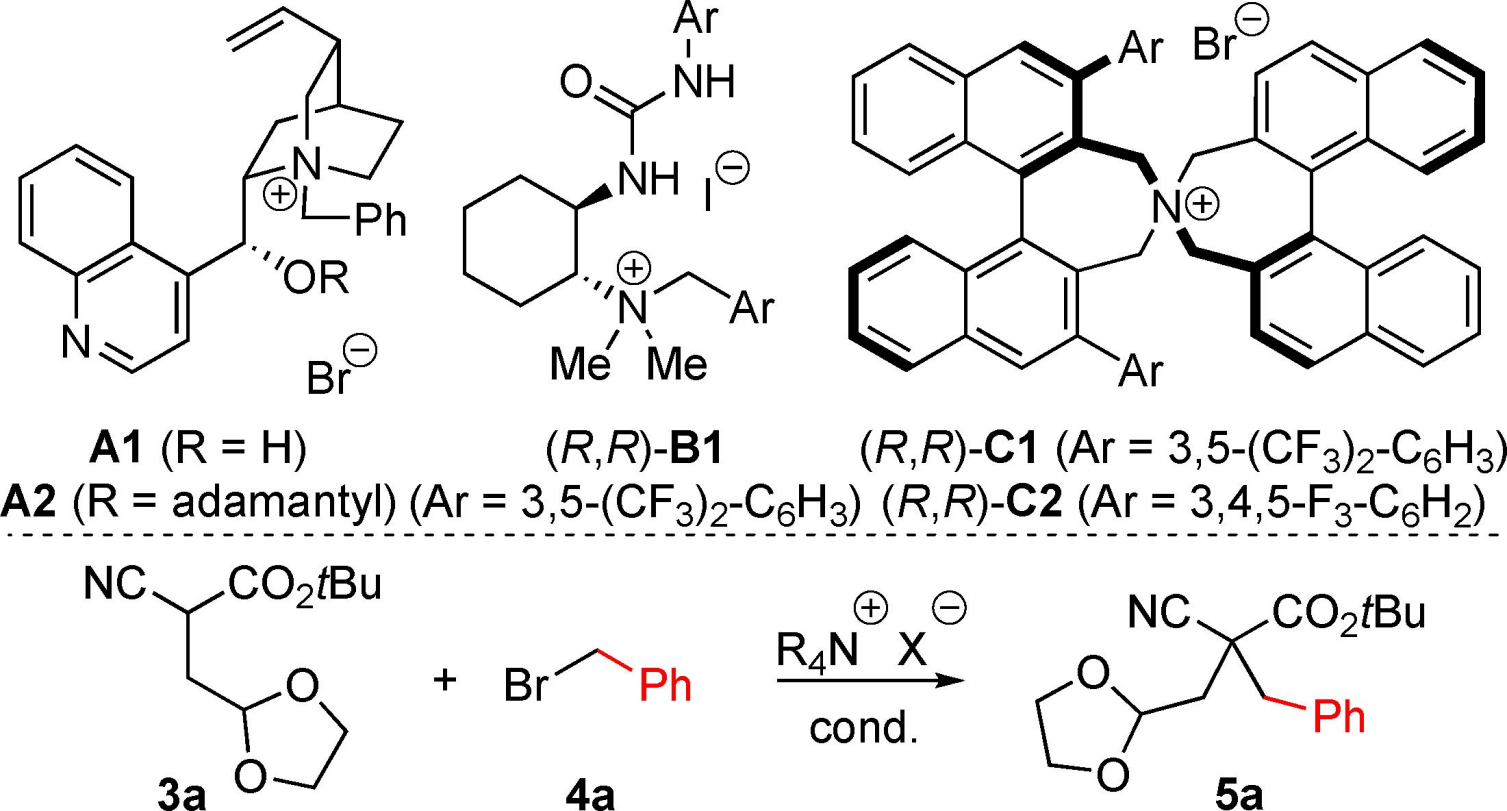							
Entry	Cat.	Solv.	Base	T [°C]	t [h]	Yield [%]^[b]^	e.r.^[c]^
1	**A1** (5 %)	toluene	KOH_(50 %)_ (30 eq.)	0	2	86	56 : 44
2	**A2** (5 %)	toluene	KOH_(50 %)_ (30 eq.)	0	2	88	51 : 49
3	**B1** (5 %)	toluene	KOH_(50 %)_ (30 eq.)	0	2	75	47 : 53
4	**C1** (5 %)	toluene	KOH_(50 %)_ (30 eq.)	0	2	87	94 : 6
5	**C1** (1 %)	toluene	KOH_(50 %)_ (30 eq.)	0	2	86	96.5 : 3.5
6	**C2** (1 %)	toluene	KOH_(50 %)_ (30 eq.)	0	2	89	84 : 16
7	**C1** (1 %)	CH_2_Cl_2_	KOH_(50 %)_ (30 eq.)	0	2	89	66 : 34
8	**C1** (1 %)	MTBE	KOH_(50 %)_ (30 eq.)	0	2	88	89 : 11
9	**C1** (1 %)	toluene	KOH_(s)_ (6 eq.)	0	2	85	95 : 5
10	**C1** (1 %)	toluene	Cs_2_CO_3(s)_ (6 eq.)	0	^20[d]^	87	88 : 12
11	**C1** (1 %)	toluene	NaOH_(50 %)_ (30 eq.)	0	2	87	93 : 7
12	**C1** (1 %)	toluene	KOH_(50 %)_ (30 eq.)	−20	^20[d]^	93	96 : 4
13	**C1** (1 %)	toluene	KOH_(50 %)_ (30 eq.)	−78	^20[d]^	70	96 : 4

[a] Unless otherwise stated all reactions were carried out using 0.1 mmol **3 a** and 0.12 mmol **4 a** in the indicated solvent (0.09 M with respect to **3 a**); [b] Isolated yields. [c] Determined by HPLC using a chiral stationary phase. Given as the ratio of (−)‐**5 a** : (+)‐**5 a**. [d] Incomplete conversion after 2 h.

Accordingly, the initially identified conditions (entry 5) remained the best‐suited, giving product **5 a** in excellent yield and very good enantioselectivity under operationally simple conditions.

**Scheme 2:**
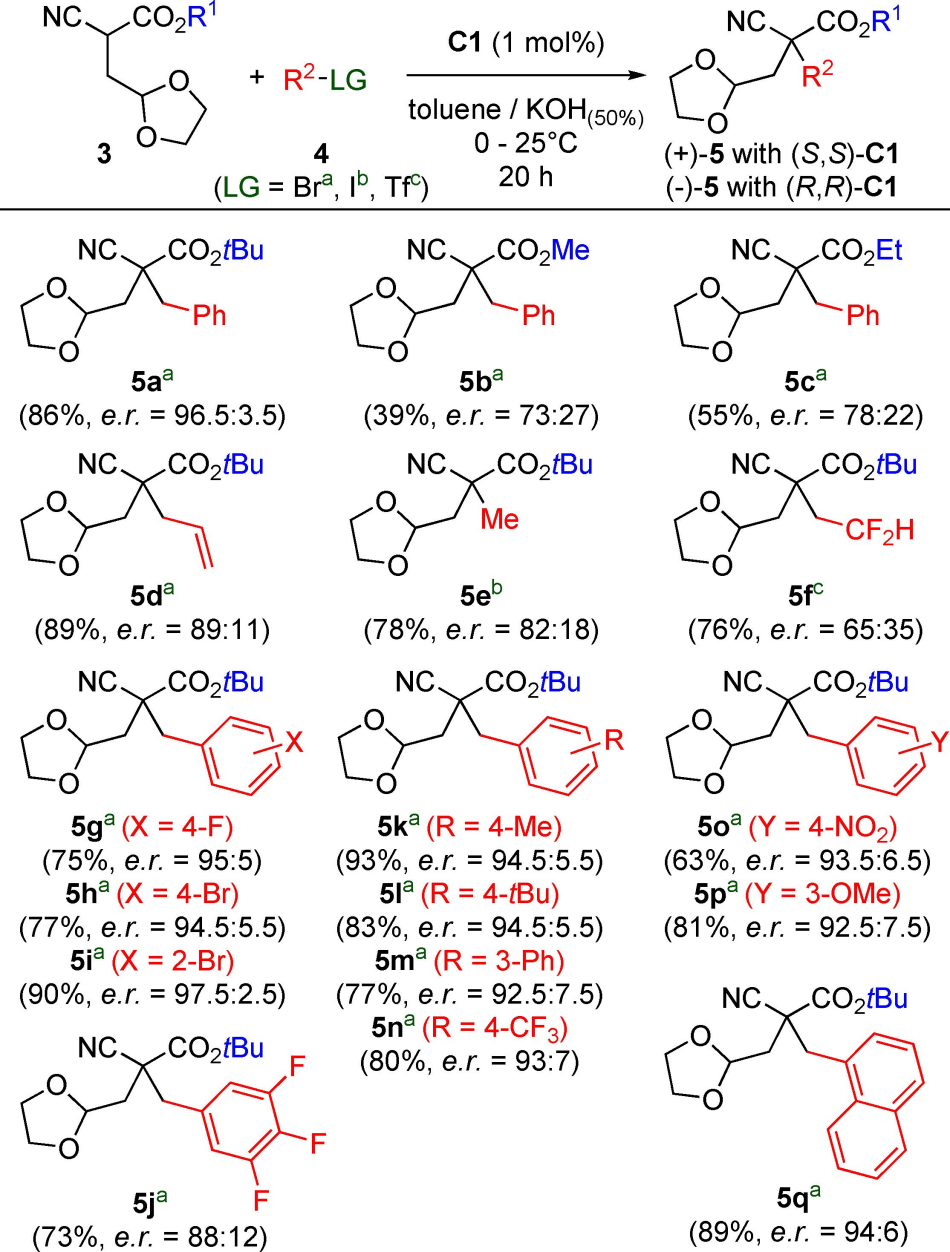
Applications scope of the asymmetric α‐alkylation of dioxolane‐containing cyanoacetates **3**.

With this optimized procedure at hand, we next investigated the application scope of this methodology by testing the synthesis of a variety of novel acyclic targets **5** containing a quaternary stereogenic center as outlined in Scheme 2 (all reactions were run for 20 h to ensure complete conversion of starting material **3** in each case). Varying the ester group R^1^ of starting material **3** revealed that less bulky esters perform clearly less selective compared to the initially used *t*‐butyl ester (compare products **5 a**–**c**). Carrying out the alkylation of **3 a** with different electrophiles showed that different benzylic halides allow for more or less similar selectivities and high yields (products **5 g**–**q**), while allylbromide (product **5 d**), methyliodide (product **5 e**) and 2,2‐difluoroethyl triflate (product **5 f**) gave lower selectivities only. Nevertheless, the application scope was in general relatively broad and robust and we also found that the use of the (*S*,*S*)‐catalyst enantiomer always gives (+)‐**5** derivatives, while (*R*,*R*)‐**C1** results in the formation of the (−)‐enantiomers exclusively.

**Scheme 3:**
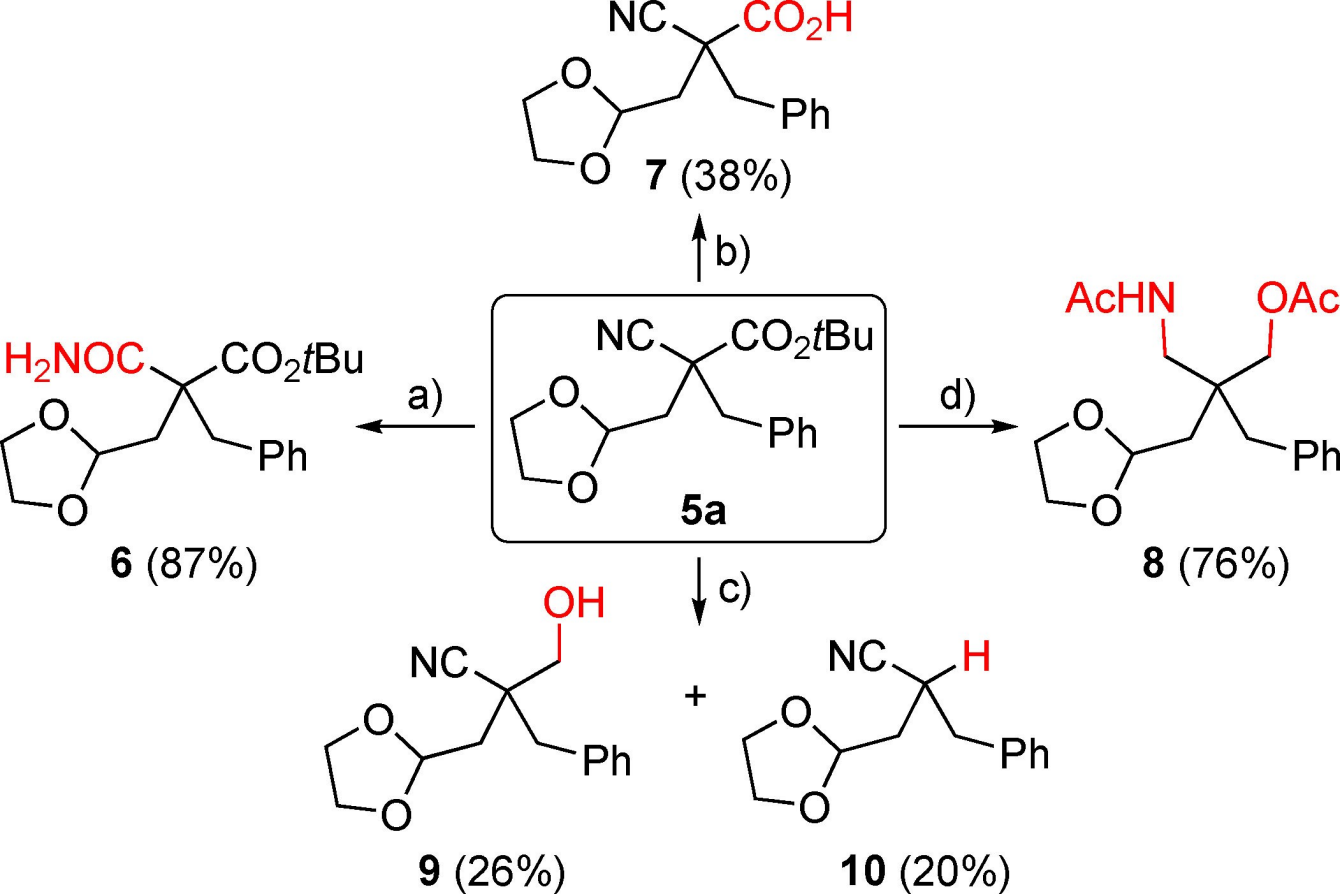
Functional group manipulations of product **5 a** [a) H_2_O_2_, Na_2_CO_3_; b) TFA; c) LiAlH_4_ (1.2 eq.); d) LiAlH_4_ (10 eq.) followed by Ac_2_O, pyridine].^[13]^

Finally, we also tested the suitability of product **5 a** to undergo further functional group manipulations (Scheme 3; all these reactions were carried out on racemic material without any extensive optimization of conditions and yield and should serve as a proof‐of‐concept mainly). It was possible to either selectivity hydrolyze the nitrile group under basic oxidative conditions (giving the primary amide‐containing **6**) or debutylate the ester under acidic conditions (accessing the free acid **7**). Surprisingly however, attempts to selectively hydrolyze the acetal failed, resulting in several unidentified decomposition products only. Furthermore, reduction with a stoichiometric amount of LiAlH_4_ resulted in the formation of a separable mixture of the alcohol **9** and the decarboxylated product **10**, while the use of an excess of LiAlH_4_ allowed for the reduction of both, the nitrile and the ester group (giving the diacetate **8** after protection with Ac_2_O), demonstrating the versatility of products **5** for further transformations.

## 3 Conclusions

Summing our investigations up, we identified robust and functional group‐tolerant conditions to carry out the asymmetric α‐alkylation of the novel orthogonally functionalized dioxolane‐containing cyanoacetates **3** under chiral ammonium salt catalysis. Key to success was the use of the structurally rigid spirocyclic Maruoka ammonium salt catalysts **C**, which gave access to the acyclic products **5**, which contain a quaternary stereogenic center, with enantioselectivities up to e.r.=97.5 : 2.5 when using just 1 mol % of the catalyst.

## Experimental Section

General details can be found in the online supporting information. This document contains the analytical data of the reaction products as well as copies of NMR spectra and HPLC traces.

**General asymmetric α‐alkylation procedure**: A solution of starting material **3** (0.10 mmol) in 1.2 mL toluene is cooled to 0 °C (Ar‐atmosphere). Subsequently, catalyst **C1** (1 mol‐%, 1.1 mg), aq. KOH_50%_ (227 μL, 30 eq) and electrophile **4** (0.12 mmol, 1.2 eq.) are added and the reaction mixture is stirred for 20 h (slow warm up to RT). Then 3.0 mL diethyl ether and 1.5 mL water are added and the phases are separated. The aqueous layer is extracted 5× with 2 mL Et_2_O each and the combined org. phases are washed with 7 mL brine. The org. phase is dried over Na_2_SO_4_, filtered over cotton and the solvent is evaporated. Purification of the crude products are performed by column chromatography using heptane:EtOAc 20/1 to 10/1.

**Product 5 a**. Prepared according to the general procedure and isolated as an almost colorless oil in 86 % yield and with e.r.=96.5 : 3.5. ^1^H‐NMR (300 MHz, CDCl_3_, 298.0 K, *δ* [ppm]): 7.28–7.37 (m, 5H), 5.19 (dd, *J_1_* =6.3 Hz, *J_2_* =3.2 Hz, 1H), 3.81–4.04 (m, 4H), 3.17 (d, *J*=13.5 Hz, 1H), 3.07 (d, *J*=13.5 Hz, 1H), 2.38 (dd, *J_1_* =14.1 Hz, *J_2_* =6.3 Hz, 1H), 2.13 (dd, *J_1_* =14.1 Hz, *J_2_* =3.2 Hz, 1H), 1.35 (s, 9H). 13 C‐NMR (75 MHz, CDCl_3_, 298.0 K, δ [ppm]): 167.1 (1C), 133.9 (1C), 130.5 (2C), 128.5 (2C), 127.9 (1C), 118.8 (1C), 101.4 (1C), 84.0 (1C), 65.3 (1C), 64.8 (1C), 47.7 (1C), 43.9 (1C), 40.4 (1C), 27.7 (3C). HRMS of C_18_H_23_NO_4_: m/z calculated for [M+NH_4_]^+^: 335.1965; found: 335.1974. [α]_D_^24^ (c=1.00, CHCl_3_)=−24.5. HPLC (Chiralpak AD‐H, hexane/i‐PrOH 10/1, 0.5 mL min^−1^, 10 °C) retention times: *t*_major_=19.0 min, *t*_minor_=26.9 min.

## Conflict of interest

The authors declare no conflict of interest.

## Supporting information

As a service to our authors and readers, this journal provides supporting information supplied by the authors. Such materials are peer reviewed and may be re‐organized for online delivery, but are not copy‐edited or typeset. Technical support issues arising from supporting information (other than missing files) should be addressed to the authors.

Supporting Information
