# Supplementary material for: Enantioselective Synthesis of Acyclic Orthogonally Functionalized Compounds Bearing a Quaternary Stereocenter Using Chiral Ammonium Salt Catalysis
Source: ChemistryOpen. 2021 Aug 5;10(8):756–9. doi: 10.1002/open.202100162 (PMC8340069; doi:10.1002/open.202100162)
Supplement: Supplementary file 1 — Supporting Information [file OPEN-10-756-s001.pdf]

ChemistryOpen

Supporting Information

Enantioselective Synthesis of Acyclic Orthogonally Functionalized Compounds Bearing a Quaternary Stereocenter Using Chiral Ammonium Salt Catalysis

Katharina Röser, Bettina Berger, Michael Widhalm,* and Mario Waser*

SUPPORTING INFORMATION

List of Contents

1. General Information 2
2. Syntheses and Application Scope 3
2.1. Syntheses of starting materials 3a-3c 3
2.2. Asymmetric α-alkylation 5
2.3. Follow-up transformations 11
3. NMR spectra of new compounds 15
4. HPLC results for the products of the application scope 42

1. General Information

NMR spectra were recorded on a Bruker Avance III 300 MHz spectrometer with a broad band observe probe and a sample changer for 16 samples, on a Bruker Avance DRX 500 MHz spectrometer and on a Bruker Avance III 700 MHz spectrometer with an Ascend magnet and TCI cryoprobe, which are property to the Austro Czech NMR Research Center “RERI uasb”. All NMR spectra were referenced on the solvent residual peak ($CDCl_3$: δ 7.26 ppm for ^1H NMR and δ 77.16 ppm for ^{13}C NMR). NMR data are reported as follows: chemical shift (δ ppm), multiplicity (s = singlet, d = doublet, t = triplet, q = quartet, m = multiplet, br = broad), coupling constants (Hz) and integrals.

High resolution mass spectra were obtained using an Thermo Fisher Scientific LTQ Orbitrap XL hybrid FT mass spectrometer with an ESI source and an Agilent G1607A coaxial sprayer, an Agilent QTOF 6520 with ESI source, and a maXis ESI-Qq-TOF. Low resolution mass spectra were obtained using an Agilent LC/MSD Trap SL.

Optical rotations were measured on a Schmidt+Haensch Unipol L 100 polarimeter ($[\alpha]_D$ values are listed in deg·cm^3·g^{-1}·dm^{-1}; concentration *c* is given in g/100 mL).

Preparative column chromatography was carried out using Davisil LC 60A 70–200 MICRON silica gel. Thin layer chromatography was performed on Macherey-Nagel pre-coated TLC plates (silica gel, 60 F_{254}, 0.20 mm, ALUGRAM® Xtra SIL). TLC plates were visualized under 254 nm UV lamp or by iodine staining in case of non UV-active substances.

Enantiomeric ratios (*e.r.*) were determined by HPLC analysis using a Dionex Summit HPLC system with a CHIRALPAK AD-H (4.6 mm × 250 mm, 5 µm) chiral stationary phase.

All chemicals were purchased from commercial suppliers and used without further purification unless otherwise stated. Until further notice reactions are carried out under argon atmosphere.

2. Syntheses and Application Scope

2.1. Syntheses of starting materials 3a-3c

Starting materials **3a-3c** were synthesized by adapting a previously reported procedure.[1]

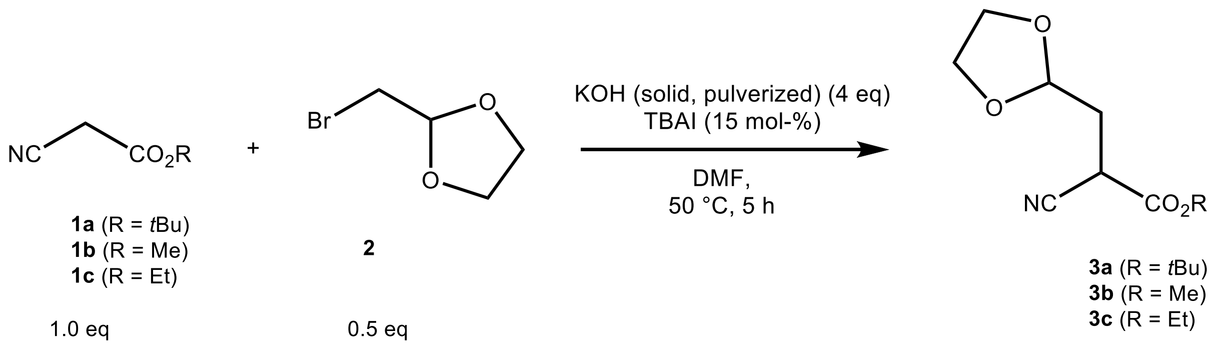

General procedure A: At r.t., 1.0 eq. of cyanoacetate **1** and 0.5 eq. of 2-bromomethyl-1,3-dioxolane (**2**) are dissolved in DMF (2 mL per mmol **1**). Under stirring, 15 mol-% TBAI and 4.0 eq. solid pulverized KOH are added and the reaction mixture is heated to 50 °C and stirred for 5 h.

The reaction mixture is allowed to cool to r.t. first and is then subsequently cooled with an ice bath and overlaid with Et_2O (around 1 mL per mmol **1**). By gentle addition of 2N HCl the pH value of the reaction mixture is set to pH 6. The separated aqueous phase is extracted 5x with Et_2O. The combined organic phases are washed once with H_2O, dried over Na_2SO_4, filtered over cotton and the solvent is evaporated.

Note: Analysis of the crude reaction mixture by ^{1}H NMR shows *in situ* yields of 30-60% (based on the limiting agent) besides unreacted starting materials **1** and **2**. Longer reaction times result in higher conversions but also increased sideproduct formation. For the subsequent asymmetric reaction clean product **3** is beneficial and purification of **3** becomes more difficult with increasing amounts of impurities.

The crude product is carefully purified by column chromatography using heptane/EtOAc 20/1 as the mobile phase and only the clean fractions (judged by ^{1}H NMR) were used for the asymmetric transformations while the impure fractions were repurified again. Product spots on TLC plates are made visible by iodine staining.

[1] A. Yu. Sizov, V. A. Dombrovskii, L. A. Yanovskaya; N. D. Zelinskii; *Russ. Chem. Bull.* **1991**, *40*, 955-961.

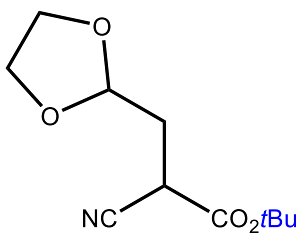

3a

Following the general procedure A (using 17.99 mmol, 2.54 g **1a**) product **3a** was obtained with 60% *in situ* yield (judged by ^{1}H NMR using an internal standard) and as a colorless to slightly yellowish clear oil in 25% yield after the first purification by column chromatography (507 mg, 2.23 mmol) together with roughly the same amount of impure **3a** that can be further purified by subsequent column chromatographic steps. ^{1}H-NMR (300 MHz, $CDCl_3$, 298.0 K, δ [ppm]): 5.08 (t, J = 4.00 Hz, 1H), 3.84-4.06 (m, 4H), 3.59 (dd, J_1 = 7.5 Hz, J_2 = 6.4 Hz, 1H), 2.21-2.37 (m, 2H), 1.49 (s, 9H). ^{13}C-NMR (75 MHz, $CDCl_3$, 298.0 K, δ [ppm]): 164.7 (1C), 116.9 (1C), 101.2 (1C), 84.2 (1C), 65.4 (1C), 65.3 (1C), 33.4 (2C), 27.9 (3C). HRMS of $C_{11}H_{17}NO_4$: m/z calculated for $[M+H]^+$: 228.1230; found: 228.1229.

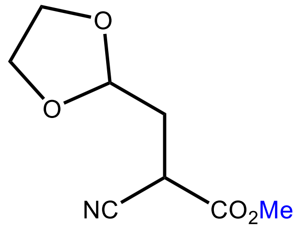

3b

Following the general procedure B (using 10 mmol, 1.0 g **1b**) product **3b** was obtained with 30% *in situ* yield (^{1}H NMR of the crude mixture) and in 14% isolated yield (132 mg, 0.7 mmol) as a colorless oil after the first purification by column chromatography. ^{1}H-NMR (300 MHz, $CDCl_3$, 298.0 K, δ [ppm]): 5.09 (t, J = 3.9 Hz, 1H), 3.86-4.07 (m, 4H), 3.82 (s, 3H), 3.72 (dd, J_1 = 7.3 Hz, J_2 = 6.5 Hz, 1H), 2.27-2.44 (m, 2H). ^{13}C-NMR (75 MHz, $CDCl_3$, 298.0 K, δ [ppm]): 166.4 (1C), 116.4 (1C), 101.0 (1C), 65.5 (1C), 65.4 (1C), 53.7 (1C), 33.4 (1C), 32.0 (1C). HRMS of $C_8H_{11}NO_4$: m/z calculated for $[M+NH_4]^+$: 203.1026; found: 203.1030.

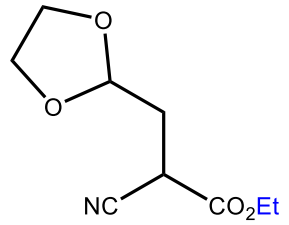

3c

Following the general procedure B (using 10 mmol, 1.13 g **1c**) product **3c** was obtained with 50% *in situ* yield (^{1}H NMR of the crude mixture) and in 21% isolated yield (223 mg, 1.05 mmol) as a colorless oil after the first purification by column chromatography. ^{1}H-NMR (300 MHz, $CDCl_3$, 298.0 K, δ [ppm]): 5.09 (t, J = 3.9 Hz, 1H), 4.27 (q, J = 7.1 Hz, 2H), 3.85-4.05 (m, 4H), 3.69 (dd, J_1 = 7.5 Hz, J_2 = 6.4 Hz, 1H), 2.26-2.43 (m, 2H), 1.32 (t, J = 7.1 Hz, 3H). ^{13}C-NMR (75 MHz, $CDCl_3$, 298.0 K, δ [ppm]): 165.9 (1C), 116.5 (1C), 101.0 (1C), 65.4 (1C), 65.3 (1C), 63.0 (1C), 33.3 (1C), 32.2 (1C), 14.0 (1C). HRMS of $C_9H_{13}NO_4$: m/z calculated for $[M+NH_4]^+$: 217.1183; found: 217.1188.

2.2. Asymmetric α-alkylation

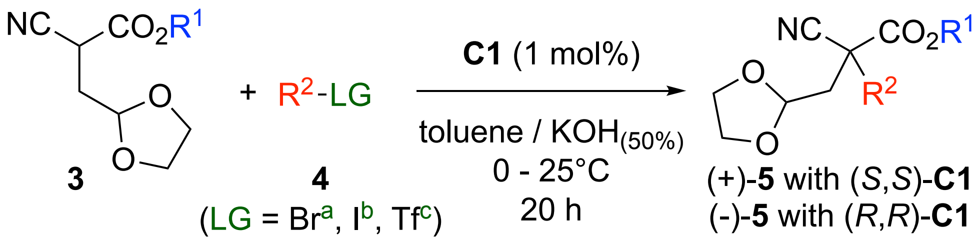

General procedure B: A solution of starting material **3** (0.10 mmol) in 1.2 mL toluene is cooled to 0 °C (Ar-atmosphere). Subsequently, catalyst **C1** (1 mol-%, 1.1 mg), aq. $KOH_{50\%}$ (30 eq, 227 µL) and electrophile **4** (0.12 mmol, 1.2 eq.) are added and the reaction mixture is stirred for 20 h (slow warm up to r.t.). Then 3.0 mL diethyl ether and 1.5 mL water are added and the phases are separated. The aqueous layer is extracted 5x with 2 mL Et_2O each and the combined org. phases are washed with 7 mL brine. The org. phase is dried over Na_2SO_4, filtered over cotton and evaporated to dryness. Purification of the crude products is performed by column chromatography using heptane/EtOAc 20/1 to 10/1.

Analytical details of products 5a-5q

5a: Prepared according to the general procedure B (LG=Br) and obtained as an almost colorless oil in 86% yield and with *e.r.* = 96.5:3.5.

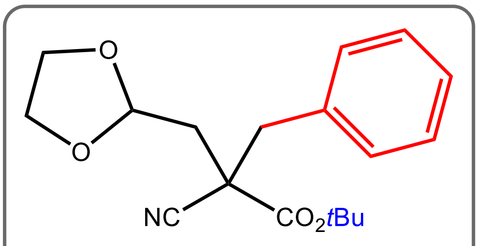

5a

^{1}H-NMR (300 MHz, $CDCl_3$, 298.0 K, δ [ppm]): 7.28-7.37 (m, 5H), 5.19 (dd, J_1 = 6.3 Hz, J_2 = 3.2 Hz, 1H), 3.81-4.04 (m, 4H), 3.17 (d, J = 13.5 Hz, 1H), 3.07 (d, J = 13.5 Hz, 1H), 2.38 (dd, J_1 = 14.1 Hz, J_2 = 6.3 Hz, 1H), 2.13 (dd, J_1 = 14.1 Hz, J_2 = 3.2 Hz, 1H), 1.35 (s, 9H). ^{13}C-NMR (75 MHz, $CDCl_3$, 298.0 K, δ [ppm]): 167.1 (1C), 133.9 (1C), 130.5 (2C), 128.5 (2C), 127.9 (1C), 118.8 (1C), 101.4 (1C), 84.0 (1C), 65.3 (1C), 64.8 (1C), 47.7 (1C), 43.9 (1C), 40.4 (1C), 27.7 (3C). HRMS of $C_{18}H_{23}NO_4$: *m/z* calculated for $[M+NH_4]^+$: 335.1965; found: 335.1974. $[\alpha]_D^{24}$ (c=1.00, $CHCl_3$) = -24.5. HPLC (Chiralpak AD-H, hexane/*i*-PrOH 10/1, 0.5 mL min^{-1}, 10 °C) retention times: t_{major} = 19.0 min, t_{minor} = 26.9 min.

5b: Prepared according to the general procedure B (LG=Br) and obtained as colorless oil in 39% yield and with *e.r.* = 73:27.

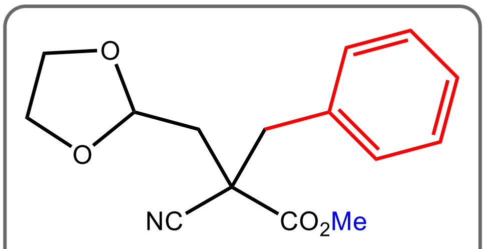

5b

^{1}H-NMR (300 MHz, $CDCl_3$, 298.0 K, δ [ppm]): 7.28-7.36 (m, 5H), 5.19 (dd, J_1 = 6.1 Hz, J_2 = 3.1 Hz, 1H), 3.84-4.04 (m, 4H), 3.71 (s, 3H), 3.25 (d, J= 13.4 Hz, 1H), 3.10 (d, J= 13.4 Hz, 1H), 2.45 (dd, J_1 = 14.2 Hz, J_2 = 6.1 Hz, 1H), 2.23 (dd, J_1 = 14.2 Hz, J_2 = 3.1 Hz, 1H). ^{13}C-NMR (75 MHz, CDCl3, 298.0 K, δ [ppm]): 169.0 (1C), 133.7 (1C), 130.2 (2C), 128.7 (2C), 128.2 (1C), 118.3 (1C), 101.3 (1C), 65.4 (1C), 65.1 (1C), 53.4 (1C), 47.3 (1C), 43.9 (1C), 40.4 (1C). HRMS of $C_{15}H_{17}NO_4$: m/z calculated for $[M+NH_4]^+$: 293.1496; found: 293.1506. $[\alpha]_D^{24}$

(c=1.00, $CHCl_3$) = +16.5. HPLC (Chiralpak AD-H, hexane/i-PrOH 10/1, 0.5 mL min^{-1}, 10 °C) retention times: t_{minor} = 30.5 min, t_{major} = 42.9 min.

5c: Prepared according to the general procedure B (LG=Br) and obtained as colorless oil in 55% yield and with *e.r.* = 78:22.

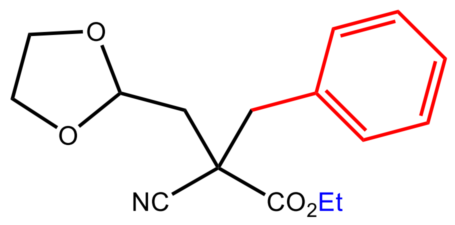

5c

^{1}H-NMR (300 MHz, $CDCl_3$, 298.0 K, δ [ppm]): 7.19-7.28 (m, 5H), 5.10 (dd, J_1 = 6.1 Hz, J_2 = 3.1 Hz, 1H), 4.02-4.13 (m, 2H), 3.74-3.95 (m, 4H), 3.13 (d, J = 13.4 Hz, 1H), 3.01 (d, J = 13.4 Hz, 1H), 2.36 (dd, J_1 = 14.2 Hz, J_2 = 6.1 Hz, 1H), 2.12 (dd, J_1 = 14.2 Hz, J_2 = 3.1 Hz, 1H), 1.10 (t, J = 7.1 Hz, 3H). ^{13}C-NMR (75 MHz, $CDCl_3$, 298.0 K, δ [ppm]): 168.4 (1C), 133.8 (1C), 130.3 (2C), 128.7 (2C), 128.1 (1C), 118.4 (1C), 101.3 (1C), 65.4 (1C), 65.0 (1C), 62.8 (1C), 47.3 (1C), 43.9 (1C), 40.3 (1C), 13.9 (1C). HRMS of $C_{16}H_{19}NO_4$: *m/z* calculated for $[M+NH_4]^+$: 307.1652; found: 307.1662. $[\alpha]_D^{24}$ (c=1.00, $CHCl_3$) = +18.7. HPLC (Chiralpak AD-H, hexane/*i*-PrOH 10/1, 0.5 mL min^{-1}, 10 °C) retention times: t_{minor} = 26.2 min, t_{major} = 38.5 min.

5d: Prepared according to the general procedure B (LG=Br) and obtained as almost colorless oil in 89% yield and with *e.r.* = 89:11.

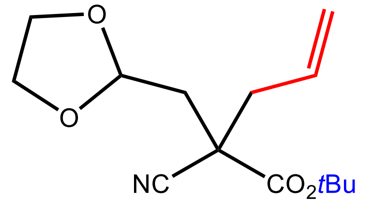

5d

^{1}H-NMR (300 MHz, $CDCl_3$, 298.0 K, δ [ppm]): 5.78-5.92 (m, 1H), 5.17 (dd, J_1 = 5.9 Hz, J_2 = 3.4 Hz, 1H), 3.81-4.04 (m, 4H), 2.62 (dd, J_1 = 13.7 Hz, J_2 = 7.2 Hz, 1H), 2.51 (dd, J_1 = 13.7 Hz, J_2 = 7.2 Hz, 1H), 2.29 (dd, J_1 = 14.2 Hz, J_2 = 5.9 Hz, 1H), 2.11 (dd, J_1 = 14.2 Hz, J_2 = 3.4 Hz, 1H), 1.50 (s, 9H). ^{13}C-NMR (75 MHz, $CDCl_3$, 298.0 K, δ [ppm]): 167.1 (1C), 130.5 (1C), 121.1 (1C), 118.6 (1C), 101.4 (1C), 84.0 (1C), 65.3 (1C), 64.9 (1C), 46.1 (1C), 42.4 (1C), 39.7 (1C), 33.3 (1C), 27.9 (3C). HRMS of $C_{14}H_{21}NO_4$: *m/z* calculated for $[M+NH_4]^+$: 285.1809; found: 285.1819. $[\alpha]_D^{24}$ (c=1.00, $CHCl_3$) = +1.2. HPLC (Chiralpak AD-H, hexane/*i*-PrOH 10/1, 0.5 mL min^{-1}, 10 °C) retention times: t_{minor} = 13.7 min, t_{major} = 14.7 min.

5e: Prepared according to the general procedure B (LG=I) and obtained as yellow oil in 78% yield and with *e.r.* = 82:18

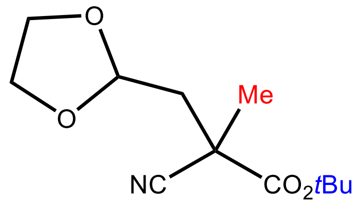

5e

^{1}H-NMR (300 MHz, $CDCl_3$, 298.0 K, δ [ppm]): 5.13 (dd, J_1 = 5.4 Hz, J_2 = 3.8 Hz, 1H), 3.82-4.03 (m, 4H), 2.33 (dd, J_1 = 14.3 Hz, J_2 = 5.5 Hz, 1H), 2.07 (dd, J_1 = 14.3 Hz, J_2 = 3.8 Hz, 1H), 1.50 (s, 9H). ^{13}C-NMR (75 MHz, $CDCl_3$, 298.0 K, δ [ppm]): 168.0 (1C), 119.9 (1C), 101.4 (1C), 83.8 (1C), 65.3 (1C), 64.9 (1C), 41.2 (1C), 41.1 (1C), 28.0 (3C), 24.6 (1C). HRMS of $C_{12}H_{19}NO_4$: *m/z* calculated for $[M+NH_4]^+$: 259.1652; found: 259.1658. $[\alpha]_D^{24}$ (c=1.00, $CHCl_3$) = +8.8. HPLC (Chiralpak AD-H, hexane/*i*-PrOH 20/1, 0.5 mL min^{-1}, 10 °C) retention times: t_{minor} = 15.4 min, t_{major} = 16.6 min.

5f: prepared according to the general procedure B (LG=Tf) and obtained as yellow oil in 76% yield and with *e.r.* = 65:35.

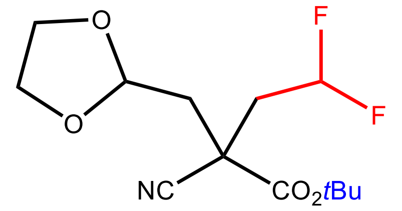

5f

^{1}H-NMR (300 MHz, $CDCl_3$, 298.0 K, δ [ppm]): 6.08 (ddt, J_1 = 55.3 Hz, J_2 = 5.8 Hz, J_3 = 3.7 Hz, 1H), 5.16 (dd, J_1 = 5.2 Hz, J_2 = 3.9 Hz, 1H), 3.83-4.04 (m, 4H), 2.11-2.59 (m, 4H), 1.51 (s, 9H). ^{13}C-NMR (75 MHz, $CDCl_3$, 298.0 K, δ [ppm]): 165.9 (1C), 117.4 (d, J = 22.1 Hz, 1C), 114.4 (1C), 100.9 (1C), 84.9 (1C), 65.2 (1C), 64.8 (1C), 41.9 (t, J = 5.3 Hz, 1C), 40.8 (dd, J_1 = 46.9 Hz, J_2 = 23.1 Hz, 1C), 27.6 (3C). ^{19}F-NMR (471 MHz, $CDCl_3$, 298.0 K, δ [ppm]): -114.73 (d-sep, J_1 = 55.3 Hz, J_2 = 9.2 Hz, 2F). HRMS of $C_{13}H_{19}F_2NO_4$: *m/z* calculated for $[M+NH_4]^+$: 309.1620; found: 309.1631. $[\alpha]_D^{24}$ (c=1.00, $CHCl_3$) = +1.5. HPLC (Chiralpak AD-H, hexane/*i*-PrOH 20/1, 0.5 mL min^{-1}, 10 °C) retention times: t_{minor} = 21.3 min, t_{major} = 22.6 min.

5g: Prepared according to the general procedure B (LG=Br) and obtained as yellow oil in 75% yield and with *e.r.* = 95:5.

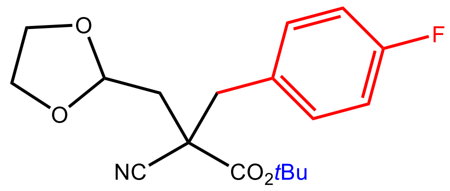

5g

^{1}H-NMR (300 MHz, $CDCl_3$, 298.0 K, δ [ppm]): 7.20-7.25 (m, 2H), 6.90-6.98 (m, 2H), 5.12 (dd, J_1 = 6.2 Hz, J_2 = 3.2 Hz, 1H), 3.75-3.97 (m, 4H), 3.05 (d, J = 13.6 Hz, 1H), 2.95 (d, J = 13.6 Hz, 1H), 2.30 (dd, J_1 = 14.1 Hz, J_2 = 6.2 Hz, 1H), 2.06 (dd, J_1 = 14.1 Hz, J_2 = 3.2 Hz, 1H), 1.29 (s, 9H). ^{13}C-NMR (75 MHz, $CDCl_3$, 298.0 K, δ [ppm]): 167.0 (1C), 162.7 (d, J = 246.5 Hz, 1C), 132.1 (d, J = 8.2 Hz, 2C), 129.8 (d, J = 3.3 Hz, 1C), 118.7 (1C), 115.4 (d, J = 21.4 Hz, 2C), 101.4 (1C), 84.2 (1C), 65.3 (1C), 64.9 (1C), 47.8 (1C), 43.0 (1C), 40.5 (1C), 27.7 (3C). ^{19}F-NMR (471 MHz, $CDCl_3$, 298.0 K, δ [ppm]): -114.53 (sep, J = 4.62 Hz, 1F). HRMS of $C_{18}H_{22}FNO_4$: *m/z* calculated for $[M+NH_4]^+$: 353.1871; found: 353.1878. $[\alpha]_D^{24}$ (c=1.00, $CHCl_3$) = +20.9. HPLC (Chiralpak AD-H, hexane/*i*-PrOH 10/1, 0.5 mL min^{-1}, 10 °C) retention times: t_{minor} = 21.7 min, t_{major} = 29.1 min.

5h: Prepared according to the general procedure B (LG=Br) and obtained as almost colorless oil in 77% yield and with *e.r.* = 94.5:5.5.

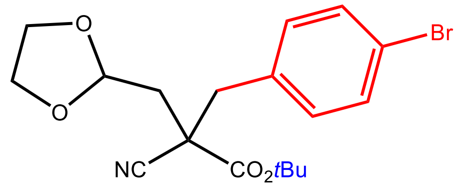

5h

^{1}H-NMR (300 MHz, $CDCl_3$, 298.0 K, δ [ppm]): 7.43-7.46 (m, 2H), 7.19-7.21 (m, 2H), 5.18 (dd, J_1 = 6.2 Hz, J_2 = 3.2 Hz, 1H), 3.83-4.03 (m, 4H), 3.11 (d, J = 13.5 Hz, 1H), 2.99 (d, J = 13.5 Hz, 1H), 2.36 (dd, J_1 = 14.1 Hz, J_2 = 6.2 Hz, 1H), 2.12 (dd, J_1 = 14.1 Hz, J_2 = 3.2 Hz, 1H), 1.37 (s, 9H). ^{13}C-NMR (75 MHz, $CDCl_3$, 298.0 K, δ [ppm]): 166.9 (1C), 133.0 (1C), 132.2 (2C), 131.7 (2C), 122.2 (1C), 118.6 (1C), 101.4 (1C), 84.3 (1C), 65.4 (1C), 64.9 (1C), 47.6 (1C), 43.2 (1C), 40.5 (1C), 27.8 (3C). HRMS of $C_{18}H_{22}BrNO_4$: *m/z* calculated for $[M+NH_4]^+$: 413.1070; found: 413.1074. $[\alpha]_D^{24}$ (c=1.00, $CHCl_3$) = +20.1. HPLC (Chiralpak AD-H, hexane/*i*-PrOH 10/1, 0.5 mL min^{-1}, 10 °C) retention times: t_{minor} = 22.8 min, t_{major} = 32.0 min.

5i: Prepared according to the general procedure B (LG=Br) and obtained as almost colorless oil in 90% yield and with *e.r.* = 97.5:2.5.

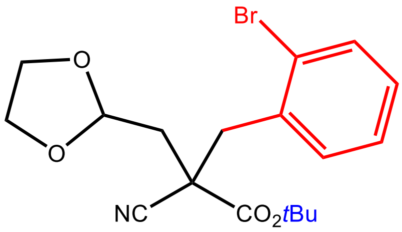

5i

^{1}H-NMR (300 MHz, $CDCl_3$, 298.0 K, δ [ppm]): 7.58 (dd, J_1 = 8.0 Hz, J_2 = 1.1 Hz, 1H), 7.54 (dd, J_1 = 7.7 Hz, J_2 = 1.6 Hz, 1H), 7.29 (td, J_1 = 7.5 Hz, J_2 = 1.1 Hz, 1H), 7.15 (td, J_1 = 7.7 Hz, J_2 = 1.6 Hz, 1H), 5.18 (dd, J_1 = 6.4 Hz, J_2 = 3.1 Hz, 1H), 3.81-4.03 (m, 4H), 3.45 (d, J = 14.1 Hz, 1H), 3.34 (d, J = 14.1 Hz, 1H), 2.49 (dd, J_1 = 14.1 Hz, J_2 = 6.4 Hz, 1H), 2.11 (dd, J_1 = 14.2 Hz, J_2 = 3.1 Hz, 1H), 1.44 (s, 9H). ^{13}C-NMR (75 MHz, $CDCl_3$, 298.0 K, δ [ppm]): 167.1 (1C), 134.0 (1C), 133.4 (1C), 131.7 (1C), 129.5 (1C), 127.7 (1C), 126.1 (1C), 118.6 (1C), 101.5 (1C), 84.2 (1C), 65.3 (1C), 64.8 (1C), 47.3 (1C), 41.8 (1C), 39.8 (1C), 27.8 (3C). HRMS of $C_{18}H_{22}BrNO_4$: *m/z* calculated for $[M+NH_4]^+$: 413.1070; found: 413.1076. $[\alpha]_D^{24}$ (c=1.00, $CHCl_3$) = +26.9. HPLC (Chiralpak AD-H, hexane/*i*-PrOH 10/1, 0.5 mL min^{-1}, 10 °C) retention times: t_{minor} = 22.3 min, t_{major} = 25.3 min.

5j: Prepared according to the general procedure B (LG=Br) and obtained as almost colorless oil in 73% yield and with *e.r.* = 88:12.

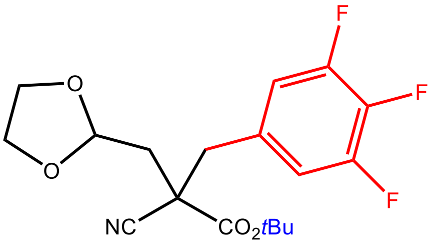

5j

^{1}H-NMR (300 MHz, $CDCl_3$, 298.0 K, δ [ppm]): 6.92-7.02 (m, 2H), 5.18 (dd, J_1 = 5.9 Hz, J_2 = 3.3 Hz), 3.83-4.04 (m, 4H), 3.11 (d, J = 13.6 Hz, 1H), 2.97 (d, J = 13.6 Hz, 1H), 2.36 (dd, J_1 = 14.1 Hz, J_2 = 6.0 Hz, 1H), 2.13 (dd, J_1 = 14.1 Hz, J_2 = 3.3 Hz, 1H), 1.40 (s, 9H). ^{13}C-NMR (75 MHz, $CDCl_3$, 298.0 K, δ [ppm]): 166.6 (1C), 151.0 (ddd, J_1 = 250.6 Hz, J_2 = 9.9 Hz, J_3 = 4.1 Hz, 1C), 139.5 (dt, J_1 = 252.4 Hz, J_2 = 15.2 Hz, 1C), 130.3-129.9 (m, 1C), 118.1 (1C), 114.7 (dd, J_1 = 14.7 Hz, J_2 = 6.7 Hz, 2C), 101.2 (1C), 84.8 (1C), 65.4 (1C), 64.9 (1C), 47.5 (1C), 42.7 (1C), 40.6 (1C), 27.8 (3C). ^{19}F-NMR (471 MHz, $CDCl_3$, 298.0 K, δ [ppm]): -133.98 (dd, J_1 = 20.4 Hz, J_2 = 7.8 Hz, 2F), -161.15 (tt, J_1 = 20.7 Hz, J_2 = 6.3 Hz, 1F). HRMS of $C_{18}H_{20}F_3NO_4$: *m/z* calculated for $[M+NH_4]^+$: 389.1682; found: 389.1685. $[\alpha]_D^{24}$ (c=1.00, $CHCl_3$) = +19.0. HPLC (Chiralpak AD-H, hexane/*i*-PrOH 10/1, 0.5 mL min^{-1}, 10 °C) retention times: t_{minor} = 18.6 min, t_{major} = 21.1 min.

5k: Prepared according to the general procedure B (LG=Br) and obtained as almost colorless oil in 93% yield and with *e.r.* = 94.5:5.5.

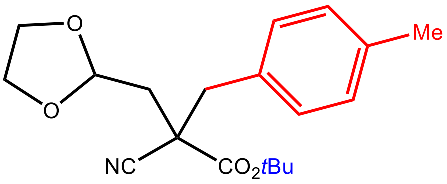

5k

^{1}H-NMR (300 MHz, $CDCl_3$, 298.0 K, δ [ppm]): 7.16 (dd, J_1 = 26.2 Hz, J_2 = 8.0 Hz, 4H), 5.18 (dd, J_1 = 6.3 Hz, J_2 = 3.2 Hz, 1H), 3.81-4.03 (m, 4H), 3.13 (d, J = 13.5 Hz, 1H), 2.99 (d, J = 13.5 Hz, 1H), 2.33-2.39 (m, 4H), 2.11 (dd, J_1 = 14.1 Hz, J_2 = 3.2 Hz, 1H), 1.38 (s, 9H). ^{13}C-NMR (75 MHz, $CDCl_3$, 298.0 K, δ [ppm]): 167.2 (1C), 137.6 (1C), 130.9 (1C), 130.3 (2C), 129.2 (2C), 118.8 (1C), 101.5 (1C), 83.9 (1C), 65.3 (1C), 64.8 (1C), 47.9 (1C), 43.5 (1C), 40.3 (1C), 27.7 (3C), 21.2 (1C). HRMS of $C_{19}H_{25}NO_4$: *m/z* calculated for $[M+NH_4]^+$: 349.2122; found: 349.2128. $[\alpha]_D^{24}$ (c=1.00, $CHCl_3$) = +20.7. HPLC (Chiralpak AD-H, hexane/*i*-PrOH 20/1, 0.5 mL min^{-1}, 10 °C) retention times: t_{minor} = 22.8 min, t_{major} = 34.3 min.

5l: Prepared according to the general procedure B (LG=Br) and obtained as yellowish oil in 83% yield and with *e.r.* = 94.5:5.5.

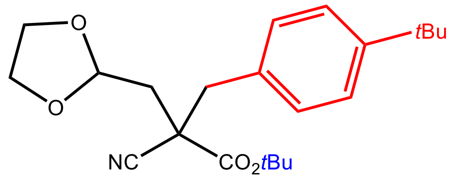

5l

^{1}H-NMR (300 MHz, $CDCl_3$, 298.0 K, δ [ppm]): 7.17-7.27 (m, 4H), 5.12 (dd, J_1 = 6.2 Hz, J_2 = 3.1 Hz, 1H), 3.76-3.96 (m, 4H), 3.04 (d, J = 13.5 Hz, 1H), 2.96 (d, J = 13.5 Hz, 1H), 2.30 (dd, J_1 = 14.1 Hz, J_2 = 6.3 Hz, 1H), 2.05 (dd, J_1 = 14.1 Hz, J_2 = 3.1 Hz, 1H), 1.28 (s, 9H), 1.23 (s, 9H). ^{13}C-NMR (75 MHz, $CDCl_3$, 298.0 K, δ [ppm]): 167.2 (1C), 150.9 (1C), 130.9 (1C), 130.2 (2C), 125.4 (2C), 119.0 (1C), 101.5 (1C), 83.8 (1C), 65.3 (1C), 64.8 (1C), 47.7 (1C), 43.5 (1C), 40.2 (1C), 34.6 (1C), 31.4 (3C), 27.7 (3C). HRMS of $C_{22}H_{31}NO_4$: *m/z* calculated for $[M+NH_4]^+$: 391.2591; found: 391.2599. $[\alpha]_D^{23}$ (c=1.00, $CHCl_3$) = +17.3. HPLC (Chiralpak AD-H, hexane/*i*-PrOH 30/1, 0.5 mL min^{-1}, 10 °C) retention times: t_{minor} = 21.5 min, t_{major} = 22.6 min.

5m: Prepared according to the general procedure B (LG=Br) and obtained as colorless oil in 77% yield and with with *e.r.* = 92.5:7.5.

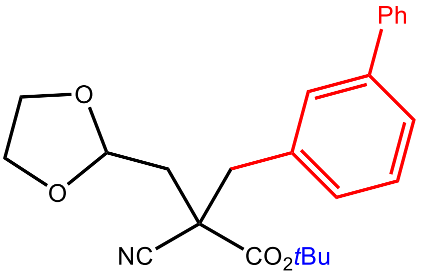

5m

^{1}H-NMR (300 MHz, $CDCl_3$, 298.0 K, δ [ppm]): 7.53-7.61 (m, 4H), 7.31-7.46 (m, 5H), 5.22 (dd, J_1 = 6.3 Hz, J_2 = 3.2 Hz, 1H), 3.83-4.05 (m, 4H), 3.22 (d, J = 13.5 Hz, 1H), 3.13 (d, J = 13.5 Hz, 1H), 2.42 (dd, J_1 = 14.1 Hz, J_2 = 6.3 Hz, 1H), 2.18 (dd, J_1 = 14.1 Hz, J_2 = 3.2 Hz, 1H), 1.34 (s, 9H). ^{13}C-NMR (75 MHz, $CDCl_3$, 298.0 K, δ [ppm]): 167.1 (1C), 141.5 (1C), 140.8 (1C), 134.5 (1C), 129.4 (1C), 129.3 (1C), 129.0 (1C), 128.9 (2C), 127.5 (1C), 127.3 (2C), 126.7 (1C), 118.9 (1C), 101.5 (1C), 84.0 (1C), 65.3 (1C), 64.8 (1C), 47.7 (1C), 44.0 (1C), 40.4 (1C), 27.7 (3C). HRMS of $C_{24}H_{27}NO_4$: *m/z* calculated for $[M+NH_4]^+$: 411.2278; found: 411.2279. $[\alpha]_D^{24}$ (c=1.00, $CHCl_3$) = +7.3. HPLC (Chiralpak AD-H, hexane/*i*-PrOH 10/1, 0.5 mL min^{-1}, 10 °C) retention times: t_{minor} = 24.4 min, t_{major} = 27.9 min.

5n: Prepared according to the general procedure B (LG=Br) and obtained as an almost colorless oil in 80% yield and with with *e.r.* = 93:7.

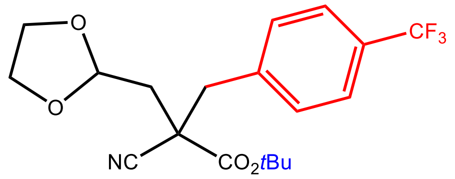

5n

^{1}H-NMR (300 MHz, $CDCl_3$, 298.0 K, δ [ppm]): 7.58 (d, J = 8.2 Hz, 2H), 7.46 (d, J = 8.1 Hz, 2H), 5.19 (dd, J_1 = 6.1 Hz, J_2 = 3.2 Hz, 1H), 3.82-4.04 (m, 4H), 3.21 (d, J = 14.4 Hz, 1H), 3.11 (d, J = 14.4 Hz, 1H), 2.40 (dd, J_1 = 14.1 Hz, J_2 = 6.1 Hz, 1H), 2.15 (dd, J_1 = 14.1 Hz, J_2 = 3.2 Hz, 1H), 1.35 (s, 9H). ^{13}C-NMR (75 MHz, $CDCl_3$, 298.0 K, δ [ppm]): 166.8 (1C), 138.1 (1C), 130.9 (2C), 130.2 (q, J = 32.6 Hz, 1C), 125.5 (q, J = 3.7 Hz, 2C), 124.0 (q, J = 271.6 Hz, 1C), 118.5 (1C), 101.3 (1C), 84.5 (1C), 65.4 (1C), 64.9 (1C), 47.5 (1C), 43.4 (1C), 40.6 (1C), 27.7 (3C). ^{19}F-NMR (471 MHz, $CDCl_3$, 298.0 K, δ [ppm]): -62.66 (3F). HRMS of $C_{19}H_{22}F_3NO_4$: *m/z* calculated for $[M+NH_4]^+$: 403.1839; found: 403.1844. $[\alpha]_D^{24}$ (c=1.00, $CHCl_3$) = +19.7. HPLC (Chiralpak AD-H, hexane/*i*-PrOH 10/1, 0.5 mL min^{-1}, 10 °C) retention times: t_{minor} = 18.3 min, t_{major} = 21.3 min.

5o: Prepared according to the general procedure B (LG=Br) and obtained as an almost colorless oil in 63% yield and with with *e.r.* = 93.5:6.5.

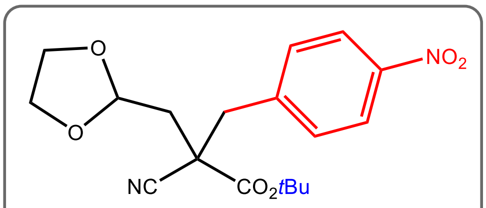

5o

^{1}H-NMR (300 MHz, $CDCl_3$, 298.0 K, δ [ppm]): 8.19 (d, J = 8.7 Hz, 2H), 7.52 (d, J = 8.7 Hz, 2H), 5.19 (dd, J_1 = 5.9 Hz, J_2 = 3.3 Hz, 1H), 3.83-4.05 (m, 4H), 3.27 (d, J = 13.4 Hz, 1H), 3.15 (d, J = 13.4 Hz, 1H), 2.41 (dd, J_1 = 14.1 Hz, J_2 = 6.00 Hz, 1H), 2.17 (dd, J_1 = 14.1 Hz, J_2 = 3.3 Hz, 1H), 1.36 (s, 9H). ^{13}C-NMR (75 MHz, $CDCl_3$, 298.0 K, δ [ppm]): 166.6 (1C), 147.8 (1C), 141.5 (1C), 131.5 (2C), 123.7 (2C), 118.2 (1C), 101.2 (1C), 84.8 (1C), 65.4 (1C), 64.9 (1C), 47.4 (1C), 43.1 (1C), 40.7 (1C), 27.8 (3C). HRMS of $C_{18}H_{22}N_2O_6$: *m/z* calculated for $[M+NH_4]^+$: 380.1816; found: 380.1820. $[\alpha]_D^{23}$ (c=1.00, $CHCl_3$) = +19.4. HPLC (Chiralpak AD-H, hexane/*i*-PrOH 10/1, 0.5 mL min^{-1}, 10 °C) retention times: t_{minor} = 47.3 min, t_{major} = 61.9 min.

5p: Prepared according to the general procedure B (LG=Br) and obtained as a slightly yellowish oil in 81% yield and with with *e.r.* = 92.5:7.5.

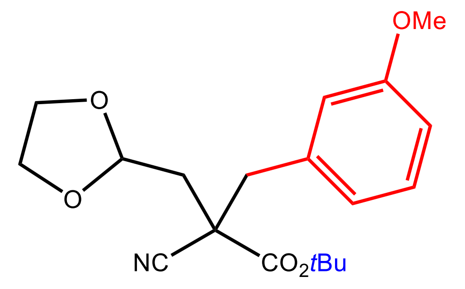

5p

^{1}H-NMR (300 MHz, $CDCl_3$, 298.0 K, δ [ppm]): 7.13-7.19 (m, 1H), 6.75-6.85 (m, 3H), 5.12 (dd, J_1 = 6.3 Hz, J_2 = 3.2 Hz, 1H), 3.75-3.96 (m, 4H), 3.73 (s, 3H), 3.06 (d, J = 13.4 Hz, 1H), 2.95 (d, J = 13.4 Hz, 1H), 2.30 (dd, J_1 = 14.1 Hz, J_2 = 6.3 Hz, 1H), 2.06 (dd, J_1 = 14.1 Hz, J_2 = 3.2 Hz, 1H), 1.31 (s, 9H). ^{13}C-NMR (75 MHz, $CDCl_3$, 298.0 K, δ [ppm]): 167.1 (1C), 159.6 (1C), 135.4 (1C), 129.5 (1C), 122.8 (1C), 118.8 (1C), 115.9 (1C), 113.7 (1C), 101.5 (1C), 84.0 (1C), 65.3 (1C), 64.8 (1C), 55.3 (1C), 47.7 (1C), 43.9 (1C), 40.4 (1C), 27.7 (3C). HRMS of $C_{19}H_{25}NO_5$: *m/z* calculated for $[M+NH_4]^+$: 365.2071; found: 365.2079. $[\alpha]_D^{24}$ (c=1.00, $CHCl_3$) = +19.6. HPLC (Chiralpak AD-H, hexane/*i*-PrOH 10/1, 0.5 mL min^{-1}, 10 °C) retention times: t_{minor} = 24.9 min, t_{major} = 31.1 min.

5q: Prepared according to the general procedure B (LG=Br) and obtained as almost colorless oil in 89% yield and with with *e.r.* = 94:6.

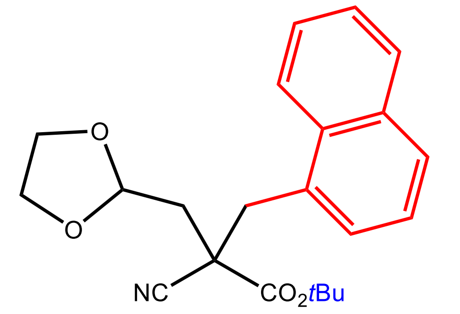

5q

^{1}H-NMR (300 MHz, $CDCl_3$, 298.0 K, δ [ppm]): 8.11 (d, J = 8.4 Hz, 1H), 7.80-7.86 (m, 2H), 7.63 (d, J = 6.9 Hz, 1H), 7.43-7.55 (m, 3H), 5.21 (dd, J_1 = 6.4 Hz, J_2 = 3.2 Hz, 1H), 3.81-4.04 (m, 4H), 3.67 (d, J = 14.2 Hz, 1H), 3.61 (d, J = 14.2 Hz, 1H), 2.52 (dd, J_1 = 14.1 Hz, J_2 = 6.4 Hz, 1H), 2.16 (dd, J_1 = 14.1 Hz, J_2 = 3.2 Hz, 1H), 1.25 (s, 9H). ^{13}C-NMR (75 MHz, $CDCl_3$, 298.0 K, δ [ppm]): 167.4 (1C), 134.0 (1C), 132.5 (1C), 130.4 (1C), 128.9 (1C), 128.8 (1C), 128.7 (1C), 126.3 (1C), 125.9 (1C), 125.3 (1C), 124.1 (1C), 119.0 (1C), 101.6 (1C), 84.0 (1C), 65.3 (1C), 64.8 (1C), 47.8 (1C), 40.5 (1C), 39.0 (1C), 27.6 (3C). HRMS of $C_{22}H_{25}NO_4$: *m/z* calculated for $[M+NH_4]^+$: 385.2122; found: 385.2125. $[\alpha]_D^{24}$ (c=1.00, $CHCl_3$) = +11.0. HPLC (Chiralpak AD-H, hexane/*i*-PrOH 10/1, 0.5 mL min^{-1}, 10 °C) retention times: t_{minor} = 25.9 min, t_{major} = 36.0 min.

2.3. Follow-up transformations

2.3.1. Procedure for conversion of the nitrile functionality into a primary amide

In accordance to a modified literature procedure,[2] 1 eq (0.10 mmol, 31.7 mg) of compound **5a** are dissolved in 2 mL EtOH and 5 eq (0.50 mmol, 53.0 mg) Na_2CO_3 are suspended in the solution. The mixture is cooled to 0 °C and 2 mL 35% aq. H_2O_2 solution is slowly added. The reaction mixture is stirred at r.t. for 69 h.

The reaction is quenched by addition of 4 mL H_2O and extracted 5x with 3 mL DCM. The organic phases are combined and twice washed with 4 mL H_2O; dried over Na_2SO_4, filtered over cotton and the solvent is evaporated.

The obtained crude product is purified by column chromatography using heptane/EtOAc 10/1 to 0/1, from which the product **6** is received as almost colorless oil in 87% isolated yield (29.3 mg, 0.087 mmol).

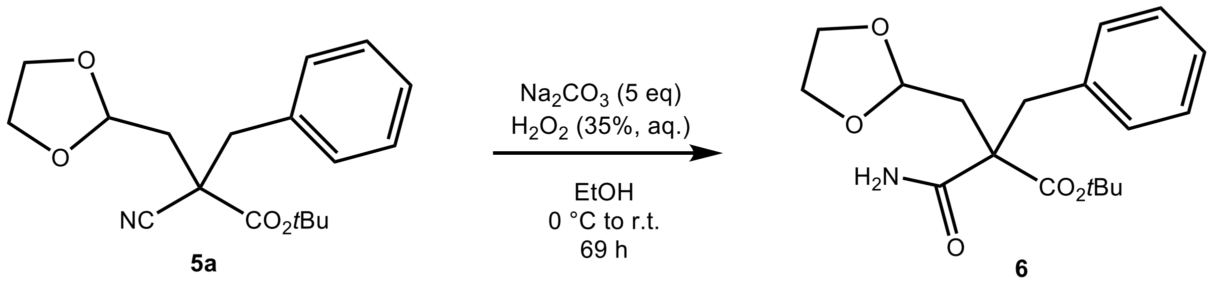

^{1}H-NMR (300 MHz, $CDCl_3$, 298.0 K, δ [ppm]): 8.06 (br s, 1H), 7.10-7.20 (m, 5H), 5.68 (br s, 1H), 4.90 (dd, J_1 = 5.6 Hz, J_2 = 4.1 Hz, 1H), 3.66-3.86 (m, 4H), 3.33 (d, J = 13.3 Hz, 1H), 2.95 (d, J = 13.3 Hz, 1H), 2.51 (dd, J_1 = 13.9 Hz, J_2 = 4.0 Hz, 1H), 2.20 (dd, J_1 = 13.9 Hz, J_2 = 5.7 Hz, 1H), 3.38 (s, 9H).

^{13}C-NMR (75 MHz, $CDCl_3$, 298.0 K, δ [ppm]): 173.4 (1C), 172.7 (1C), 136.3 (1C), 130.0 (2C), 128.2 (2C), 127.1 (1C), 102.1 (1C), 82.8 (1C), 65.0 (1C), 64.8 (1C), 56.0 (1C), 44.6 (1C), 41.7 (1C), 28.0 (3C).

HRMS of $C_{18}H_{25}NO_5$: *m/z* calculated for $[M+H]^+$: 336.1806; found: 336.1809.

2.3.2. Procedure for conversion of the ester functionality into the free carboxylic acid

Following a modified literature procedure, [3] at r.t. 1 eq (0.10 mmol, 31.8 mg) of compound **5a** is dissolved in 2 mL DCM and 10 eq (77.0 µL) of trifluoroacetic acid (TFA) are added. After stirring the reaction mixture at r.t. for 24 h the solvent and the excess of TFA is evaporated.

The product **7** is isolated as a white solid in 38% yield (10.0 mg, 0.038 mmol) after purification by column chromatography using heptane/DCM 1/4 and further purification by preparative TLC (silica gel plate; heptane/EtOAc 3/4).

[2] M. Du, L. Yu, T. Du, Z. Li, Y. Luo, X. Meng, Z. Tian, C. Zheng, W. Cao, G. Zhao; *Chem. Commun.*, **2020**, *56*, 1581-1584.
[3] Y. Odanaka, T. Kanemitsu, K. Iwasaki, Y. Mochizuki, M. Miyazaki, K. Nagata, M. Kato, T. Itoh; *Tetrahedron*, **2019**, *75*, 209-219.

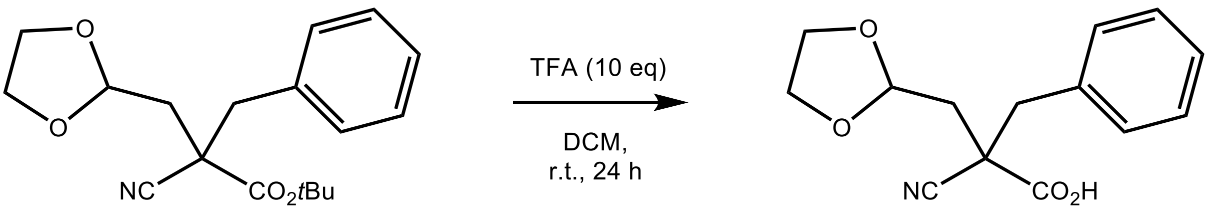

5a **7**

^{1}H-NMR (300 MHz, $CDCl_3$, 298.0 K, δ [ppm]): 7.25-7.38 (m, 5H), 5.28 (dd, J_1 = 4.7 Hz, J_2 = 2.9 Hz, 1H), 3.89-3.91 (m, 1H), 3.73-3.81 (m, 2H), 3.65-3.68 (m, 1H), 3.35 (d, J = 13.9 Hz, 1H), 3.23 (d, J = 13.9 Hz, 1H), 2.52-2.58 (m, 2H).

^{13}C-NMR (176 MHz, $CDCl_3$, 298.0 K, δ [ppm]): 169.9 (1C), 132.9 (1C), 130.2 (2C), 129.3 (2C), 128.6 (1C), 118.0 (1C), 102.3 (1C), 71.6 (1C), 61.6 (1C), 43.4 (1C), 41.2 (1C), 38.4 (1C).

HRMS of $C_{14}H_{15}NO_4$: *m/z* calculated for $[M+NH_4]^+$: 279.1339; found: 279.1344.

2.3.3. Procedure for simultaneous reduction and acetylation of both nitrile and ester functionalities

Applying modified literature procedure,[4] 1 eq (0.164 mmol, 52.0 mg) of substance 5**a** is dissolved in 4 mL dry THF. At 0 °C, a suspension of 10 eq (1.823 mmol, 69.2 mg) $LiAlH_4$ in 3 mL dry THF is added dropwise and the reaction mixture is stirred for 4 h. The reaction is quenched by careful addition of 6 mL EtOAc and 6 mL H_2O and solids are removed by filtration over Celite. The phases of the filtrate are separated and the aqueous phase is extracted 3x with 8 mL EtOAc. The combined organic phases are washed with 8 mL water and the solvent is evaporated. To the obtained residue 2 mL pyridine and 5 eq (82.0 µL) acetic anhydride are added and the reaction mixture is stirred at 70 °C for 17 h. The reaction mixture is concentrated *in vacuo*, the obtained residue is dissolved in 15 mL DCM and twice washed with 5 mL sat. Na_2CO_3-solution (aq.). DCM is evaporated, from which the crude product is received. Purification is performed by column chromatography using heptane/EtOAc 10/1 to 2/1 to 0/1, from which the diacetylated product **8** is isolated as yellowish oil in 85% yield (46.8 mg, 0.140 mmol).

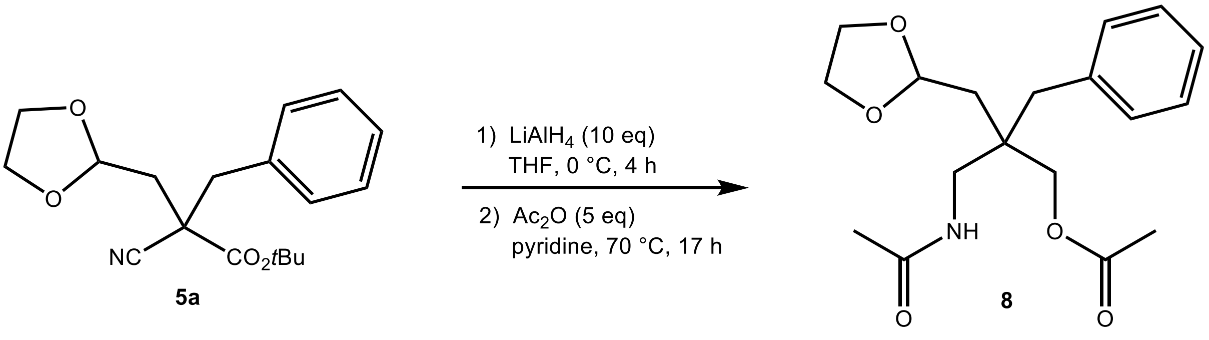

5a **8**

^{1}H-NMR (300 MHz, $CDCl_3$, 298.0 K, δ [ppm]): 7.13-7.30 (m, 5H), 6.27 (br s, 1H), 5.01 (t, J = 4.7 Hz, 1H), 3.82-4.05 (m, 5H), 3.70 (d, J = 11.4 Hz, 1H), 3.48 (dd, J_1 = 13.9 Hz, J_2 = 7.0 Hz, 1H), 3.25 (dd, J_1 = 14.0 Hz, J_2 = 6.1 Hz, 1H), 2.80 (d, J = 13.6 Hz, 1H), 2.68 (d, J = 13.6 Hz, 1H), 2.12 (s, 3H), 1.96 (s, 3H), 1.74 (dd, J_1 = 15.0 Hz, J_2 = 4.7 Hz, 1H), 1.59 (dd, J_1 = 15.0 Hz, J_2 = 4.9 Hz, 1H).

[4] A. G. Du Bellay; *Synthesis of ligands of Urotensin II receptor and Melatonin receptors. Pyrido[2,3-d]pyrimidinic or imidazo[1,2-a]pyridinic compounds*, PhD Thesis, University of Orléans, **2008**.

^{13}C-NMR (75 MHz, $CDCl_3$, 298.0 K, δ [ppm]): 171.1 (1C), 170.3 (1C), 136.5 (1C), 130.7 (2C), 128.4 (2C), 126.7 (1C), 101.8 (1C), 66.1 (1C), 64.9 (d, J=4.20 Hz, 1C), 43.2 (1C), 40.5 (1C), 40.1 (1C), 36.4 (1C), 29.8 (1C), 23.7 (1C), 21.1 (1C).

HRMS of $C_{18}H_{25}NO_5$: *m/z* calculated for $[M+Na]^+$: 358.1625; found: 358.1626.

2.3.4. Procedure for reduction of compound **5a**

In accordance to the modified literature procedure,[5] 1.0 eq (0.24 mmol, 75.9 mg) of compound **5a** are dissolved in 3.6 mL dry diethyl ether. At 0 °C, a suspension of 1.2 eq (11.1 mg) $LiAlH_4$ in 390 mL dry Et_2O is dropwise added. The reaction mixture is stirred overnight (22 h) at room temperature. The reaction is quenched by careful addition of 3 mL water. The solids are filtered off by suction filtration (Por. 3) and subsequently washed with Et_2O, which is collected in a separate round bottom flask and removed by concentration *in vacuo* yielding 29.0 mg slightly yellowish oil. In order to enhance the yield of the reaction, the filtrate consisting of the reaction solvent and the water from quenching the reaction is subjected to extraction: The separated aqueous phase is extracted 4x with 3 mL Et_2O and the combined organic layers are once washed with 2 mL water. The solvent is evaporated yielding another 34.8 mg yellowish oil. The obtained oily crude products of both work-up steps are combined. 38.7 mg of the combined crude products are purified by column chromatography (silica gel; heptane/EtOAc 10/1 to 2/1). In addition to recovering educt **5a** (23%, 13.3 mg, 0.042 mmol; Rf=0.71 in heptane/EtOAc 1/1), two compounds are isolated, alcohol **9** (Rf=0.46 in heptane/EtOAc 1/1) in 26% yield (11.9 mg, 0.048 mmol) as the main product of the reaction and decarboxylated compound **10** (20%, 8.1 mg, 0.037 mmol; Rf=0.64 in heptane/EtOAc 1/1). (The given yields are calculated for the isolated substances from column chromatography under consideration of the mass of the crude product subjected to purification)

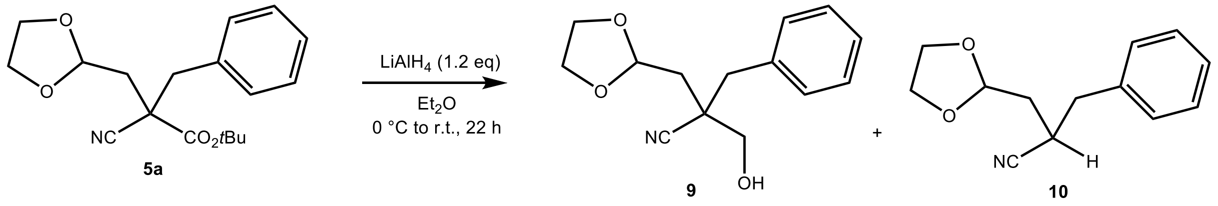

Compound 9: ^{1}H-NMR (300 MHz, $CDCl_3$, 298.0 K, δ [ppm]): 7.32-7.37 (m, 5H), 5.10 (dd, J_1 = 6.7 Hz, J_2 = 2.6 Hz, 1H), 3.81-4.02 (m, 5H), 3.67 (dd, J_1 = 11.7 Hz, J_2 = 8.1 Hz, 1H), 2.90-3.03 (m, 3H), 2.10 (dd, J_1 = 14.7 Hz, J_2 = 2.5 Hz, 1H), 1.92 (dd, J_1 = 14.8 Hz, J_2 = 6.7 Hz, 1H).

^{13}C-NMR (176 MHz, $CDCl_3$, 298.0 K, δ [ppm]): 134.7 (1C), 130.6 (2C), 128.7 (2C), 127.7 (1C), 121.4 (1C), 101.9 (1C), 65.5 (1C), 64.9 (1C), 64.8 (1C), 41.8 (1C), 41.1 (1C), 37.9 (1C).

HRMS of $C_{14}H_{17}NO_3$: *m/z* calculated for $[M+NH_4]^+$: 265.1546; found: 265.1554.

[5] M. Youngman, W. M. Kazmierski, H. Yang, C. J. Aquino; PTC Int. Appl. 2004055012, 01 Jul **2004**.

Compound 10: ^{1}H-NMR (300 MHz, $CDCl_3$, 298.0 K, δ [ppm]): 7.27-7.39 (m, 5H), 5.08 (dd, J_1 = 5.6 Hz, J_2 = 3.5 Hz, 1H), 3.88-4.02 (m, 4H), 2.96-3.11 (m, 3H), 2.08 (ddd, J_1 = 14.5 Hz, J_2 = 9.3 Hz, J_3 = 3.4 Hz, 1H), 1.90 (dd, J_1 = 14.1 Hz, J_2 = 5.2 Hz, 1H).

^{13}C-NMR (176 MHz, $CDCl_3$, 298.0 K, δ [ppm]): 136.7 (1C), 129.3 (2C), 128.9 (2C), 127.5 (1C), 121.5 (1C), 101.9 (1C), 65.4 (1C), 65.2 (1C), 38.7 (1C), 35.6 (1C), 28.7 (1C).

HRMS of $C_{13}H_{15}NO_2$: *m/z* calculated for $[M+Na]^+$: 240.0995; found: 240.0998.

3. NMR spectra of new compounds

NMR spectra of **3a**

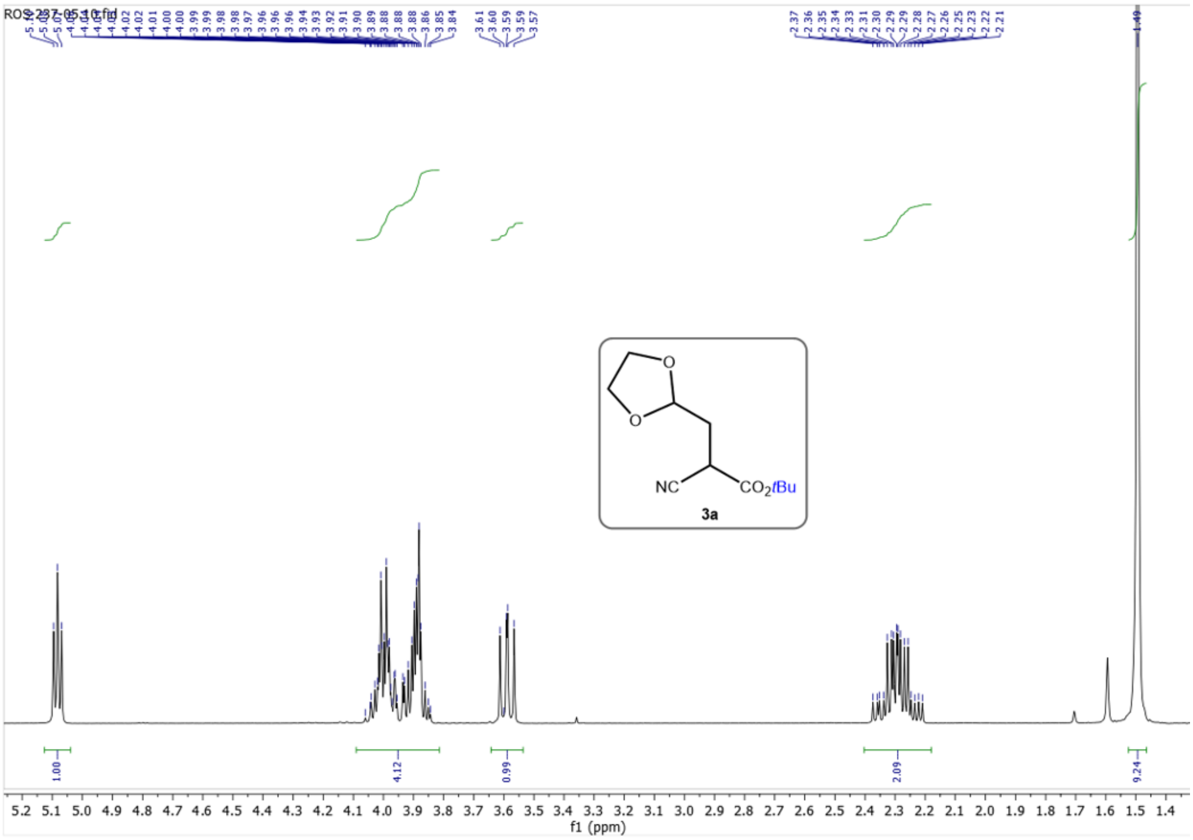

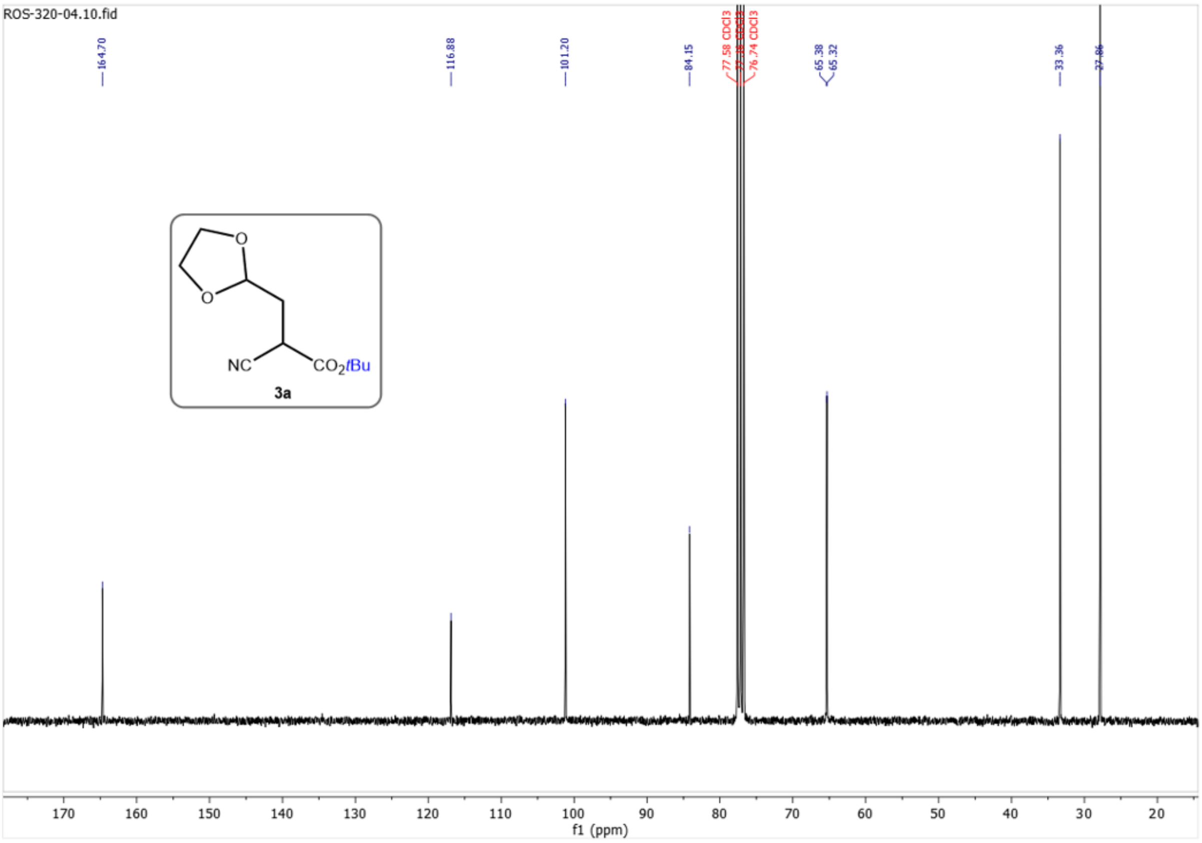

NMR spectra of **3b**

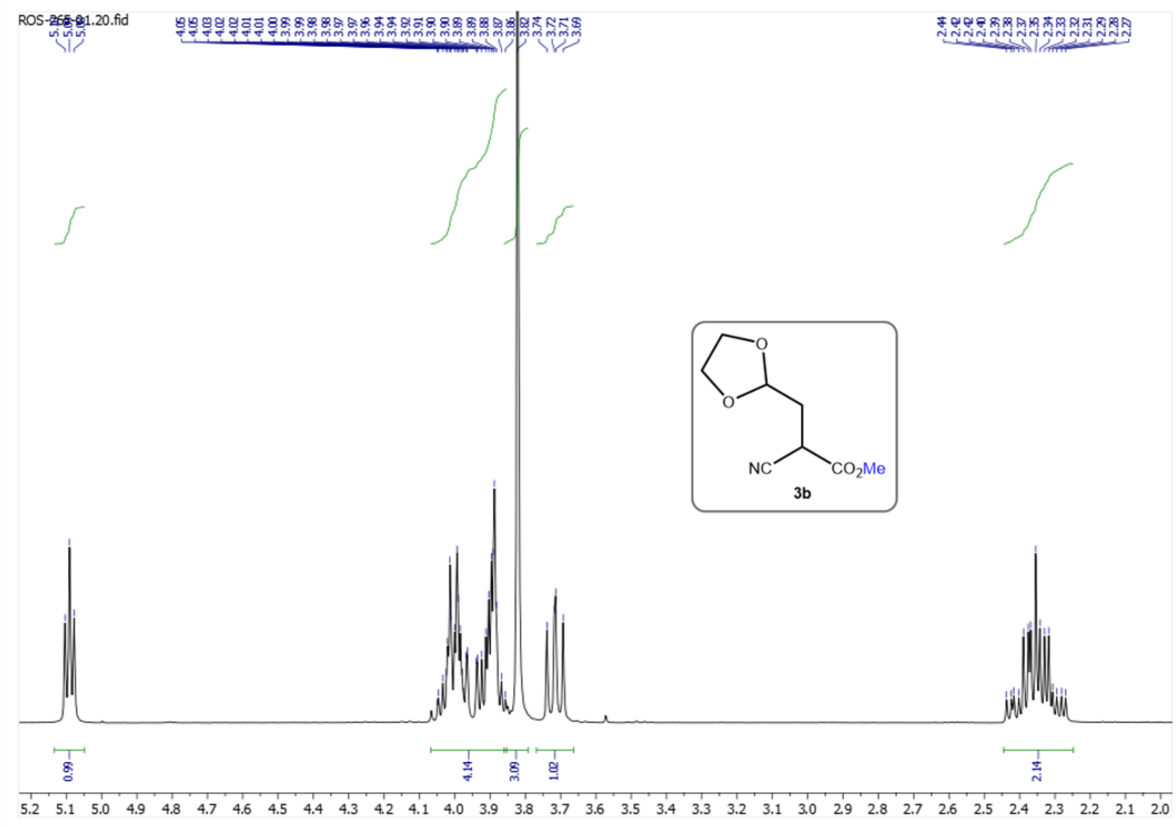

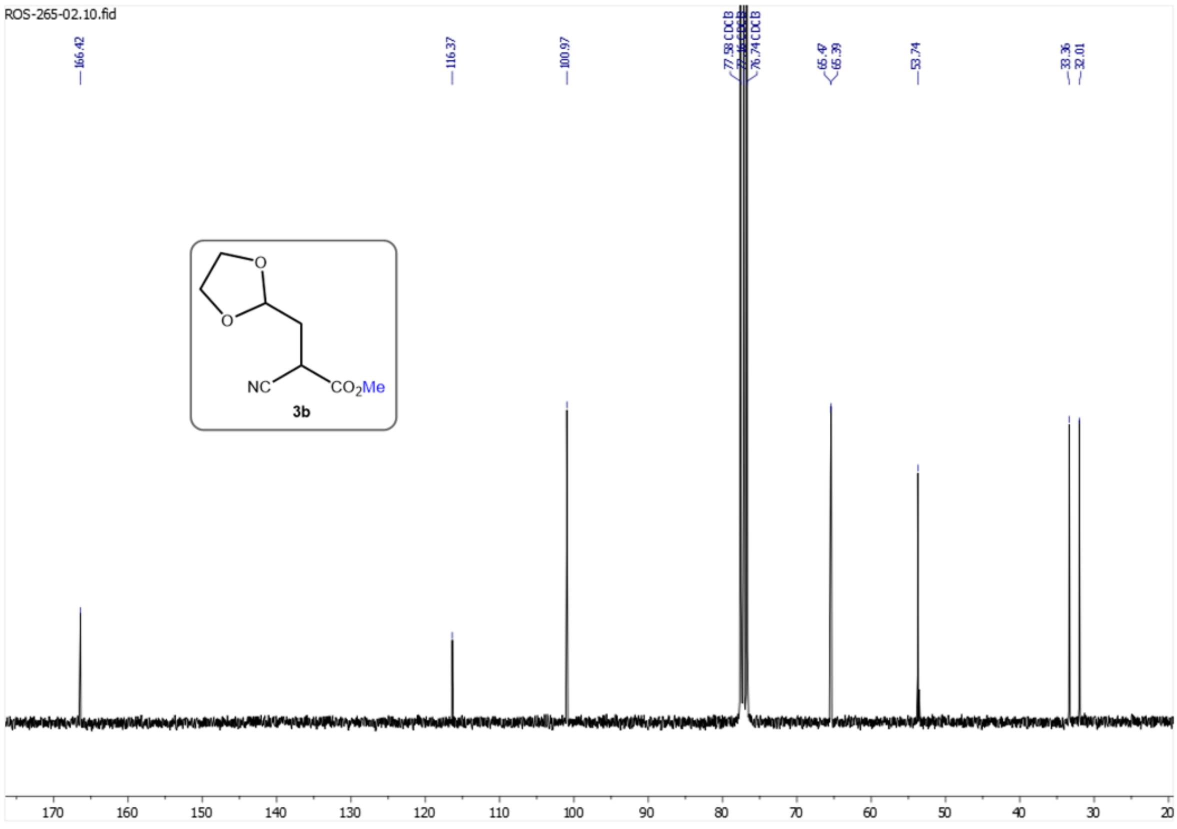

NMR spectra of **3c**

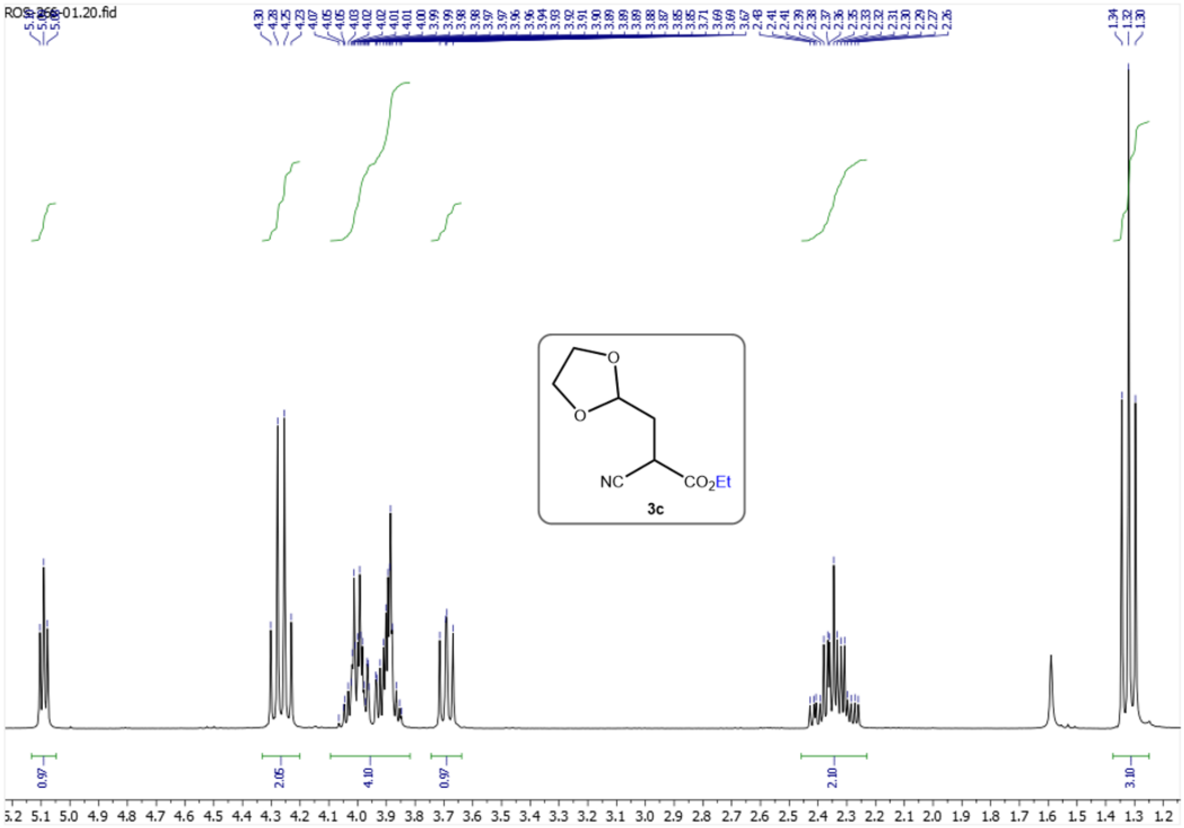

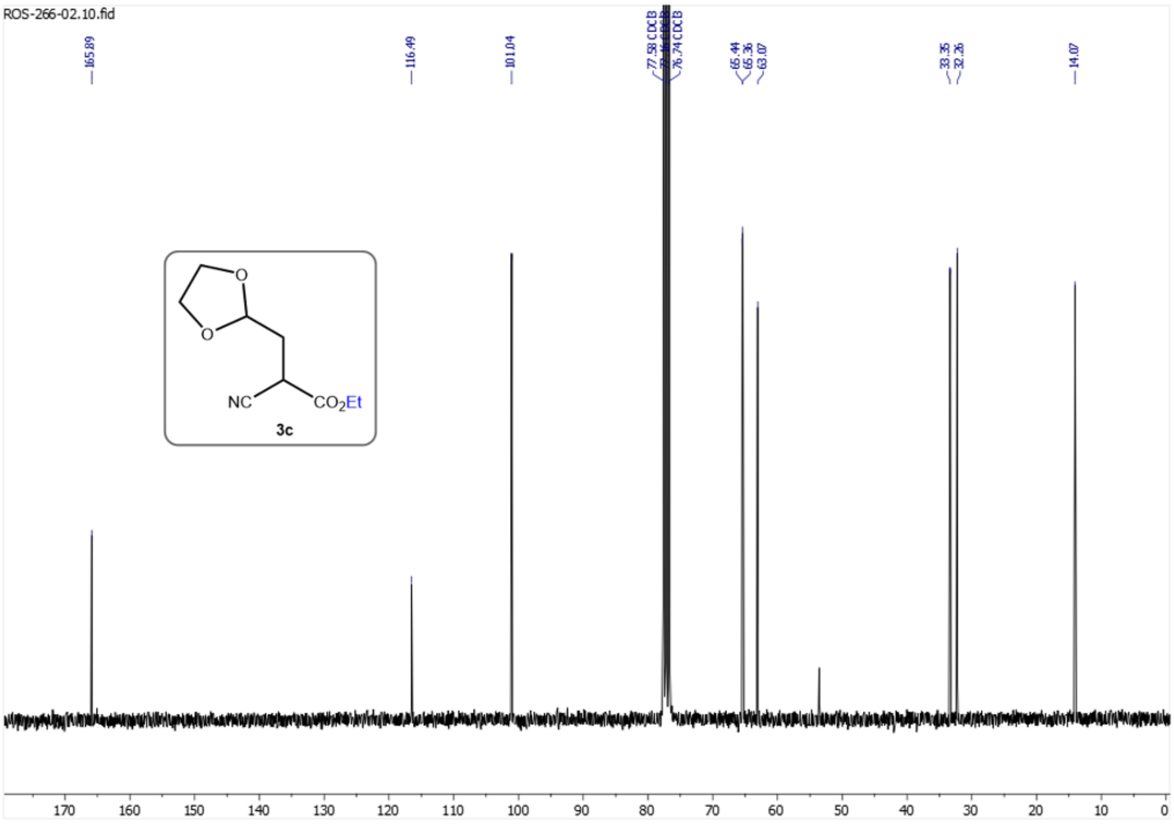

NMR spectra of 5**a**

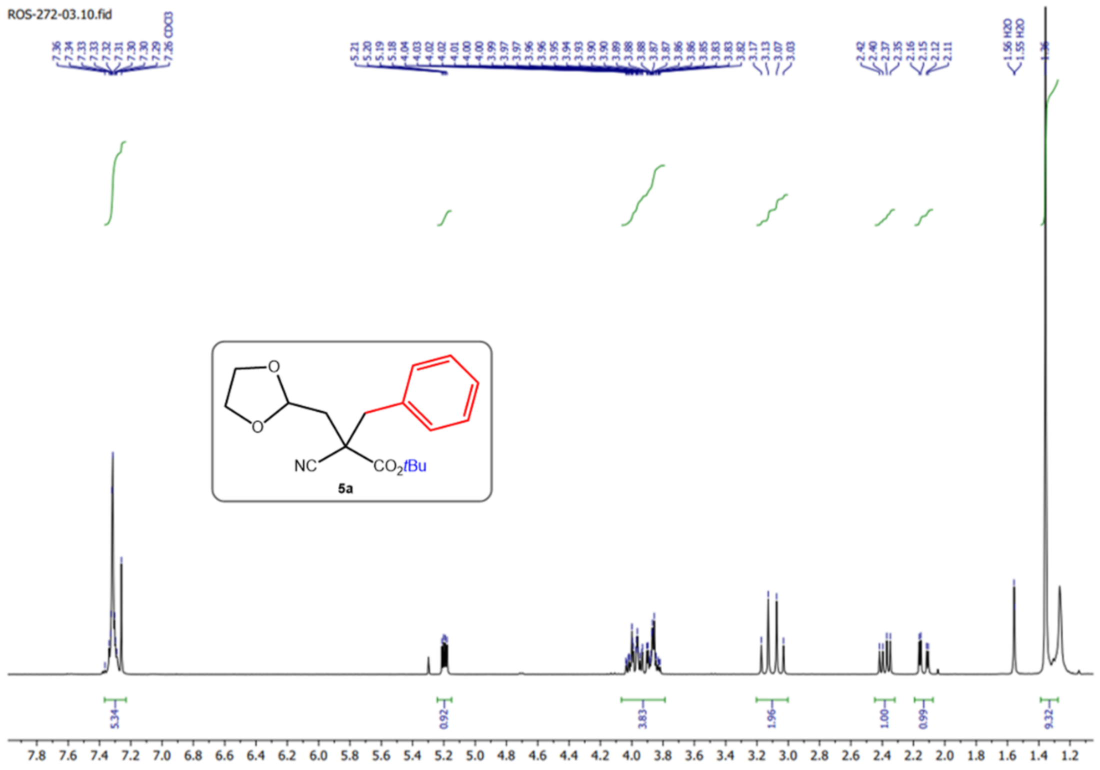

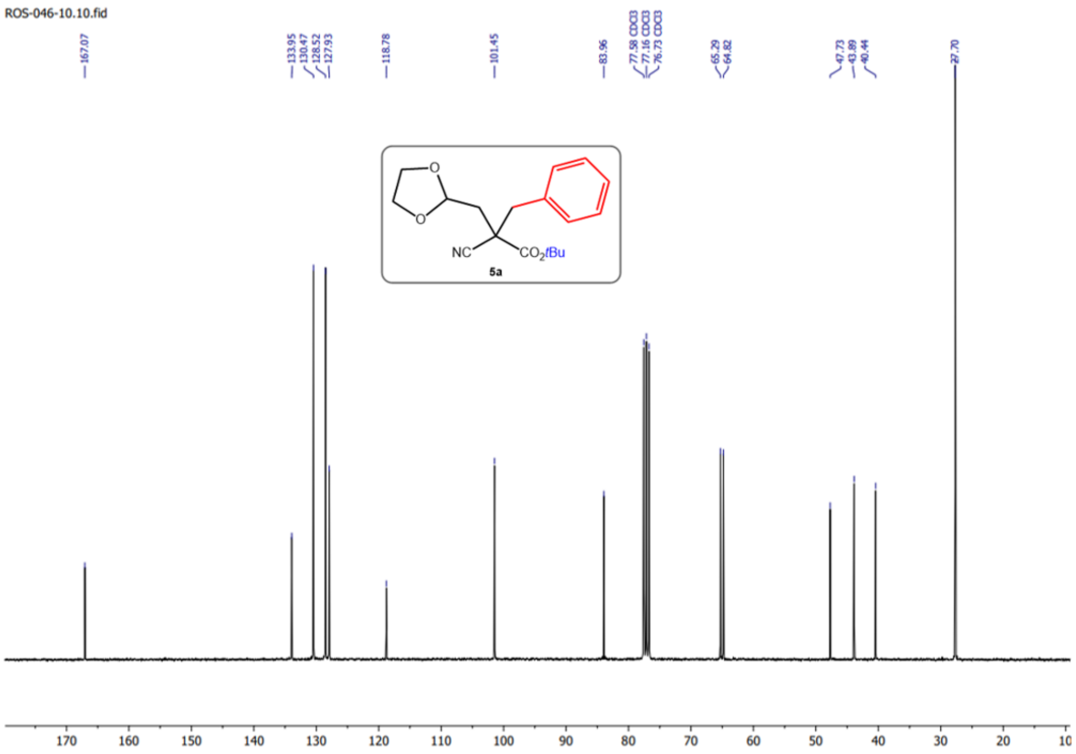

NMR spectra of **5b**

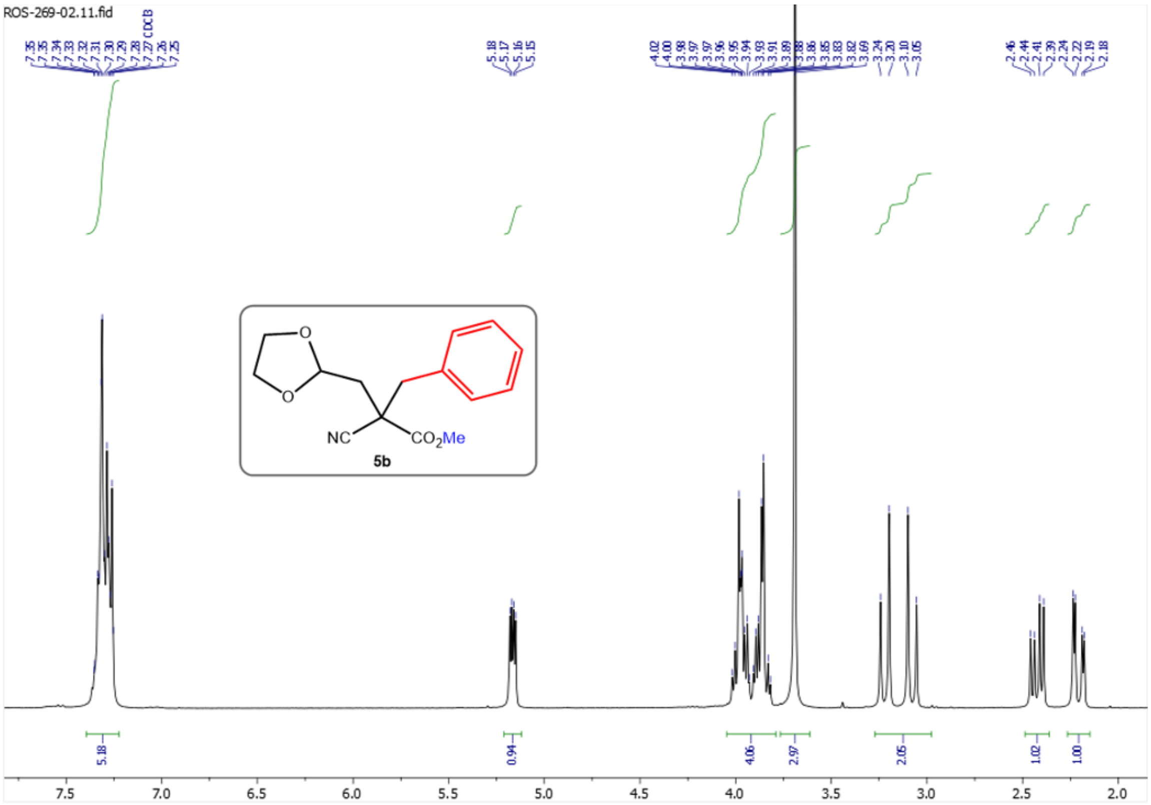

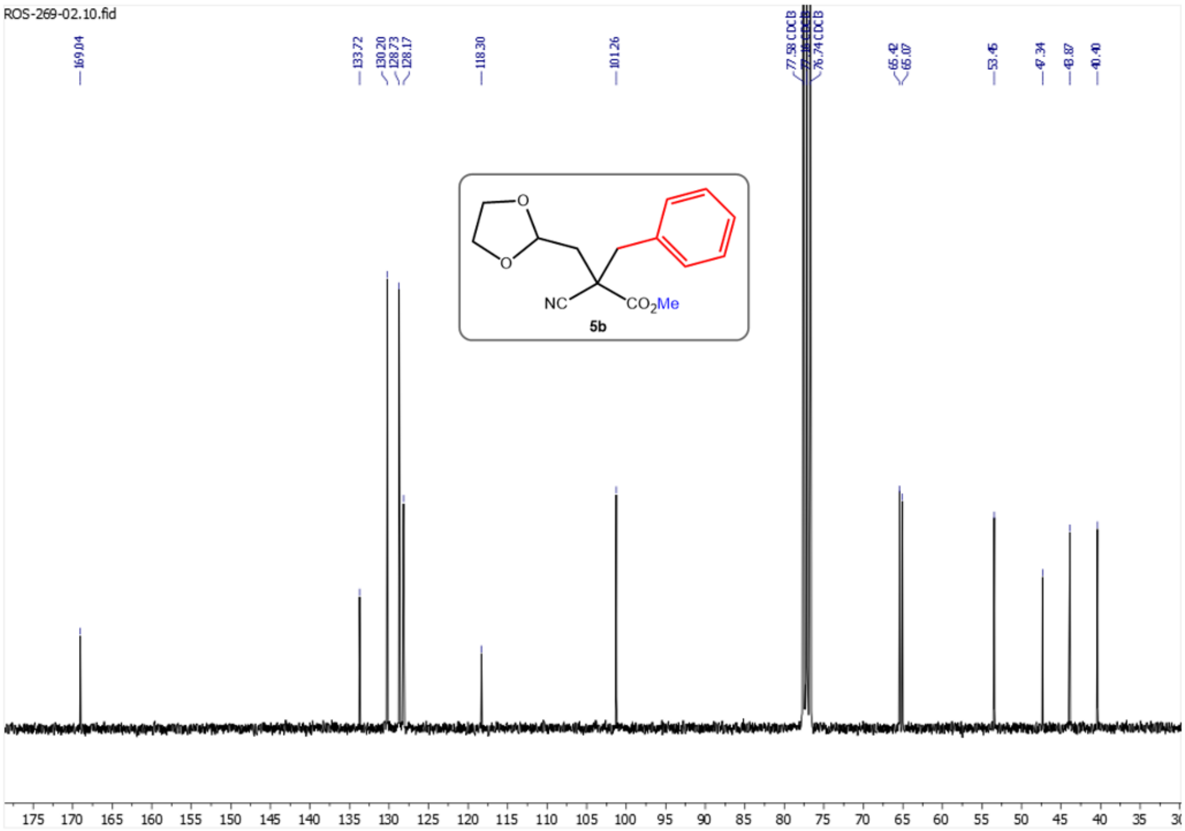

NMR spectra of 5c

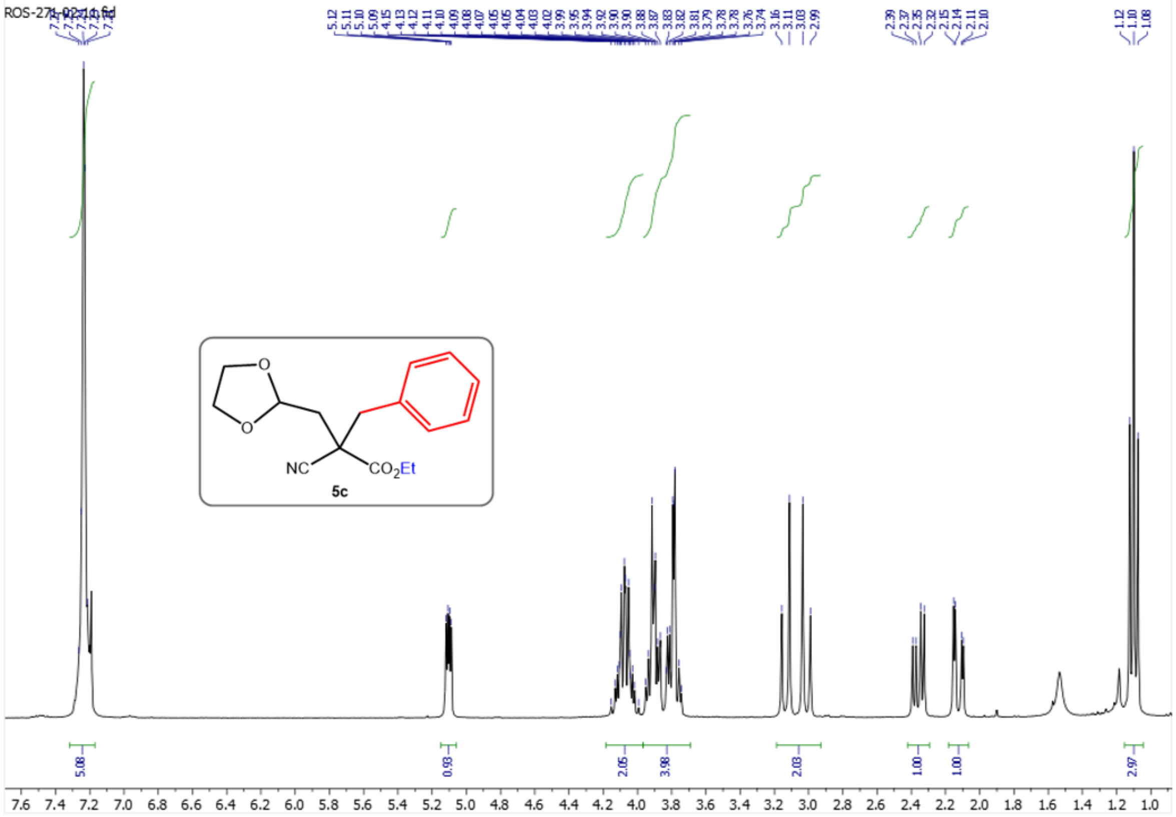

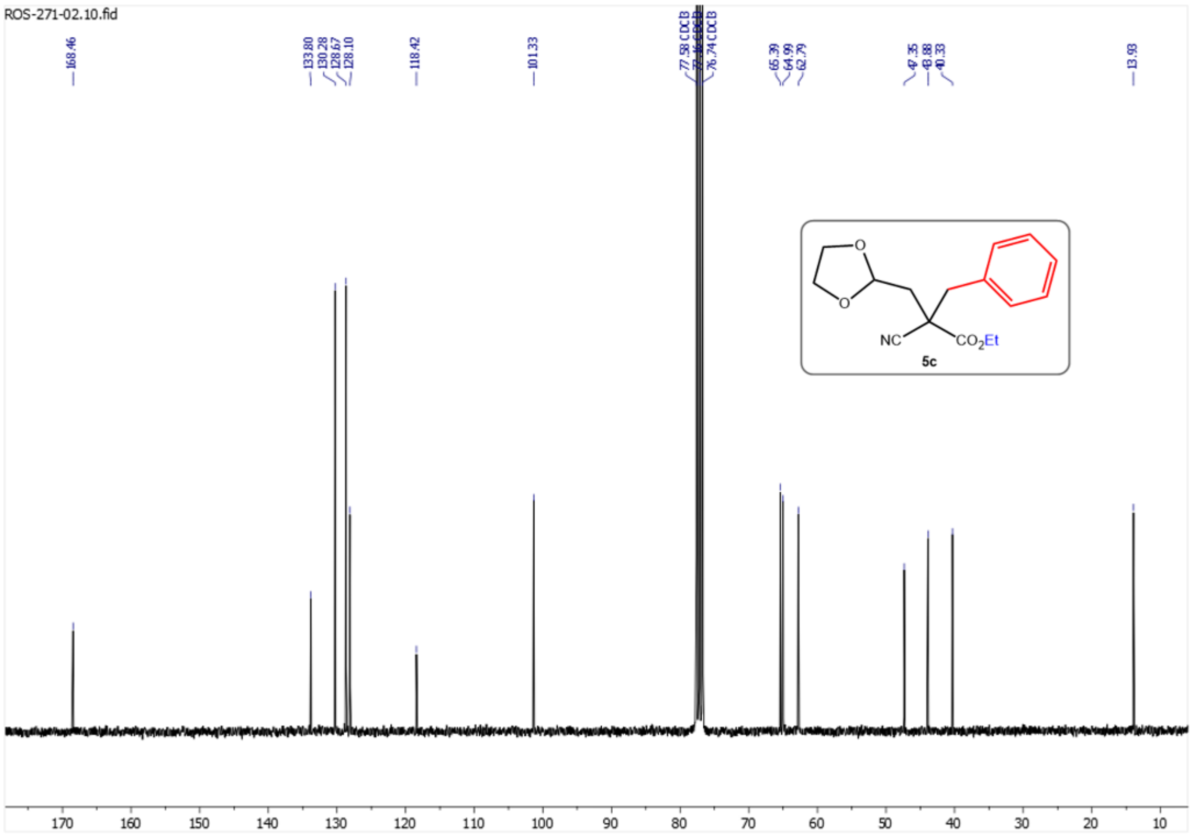

NMR spectra of **5d**

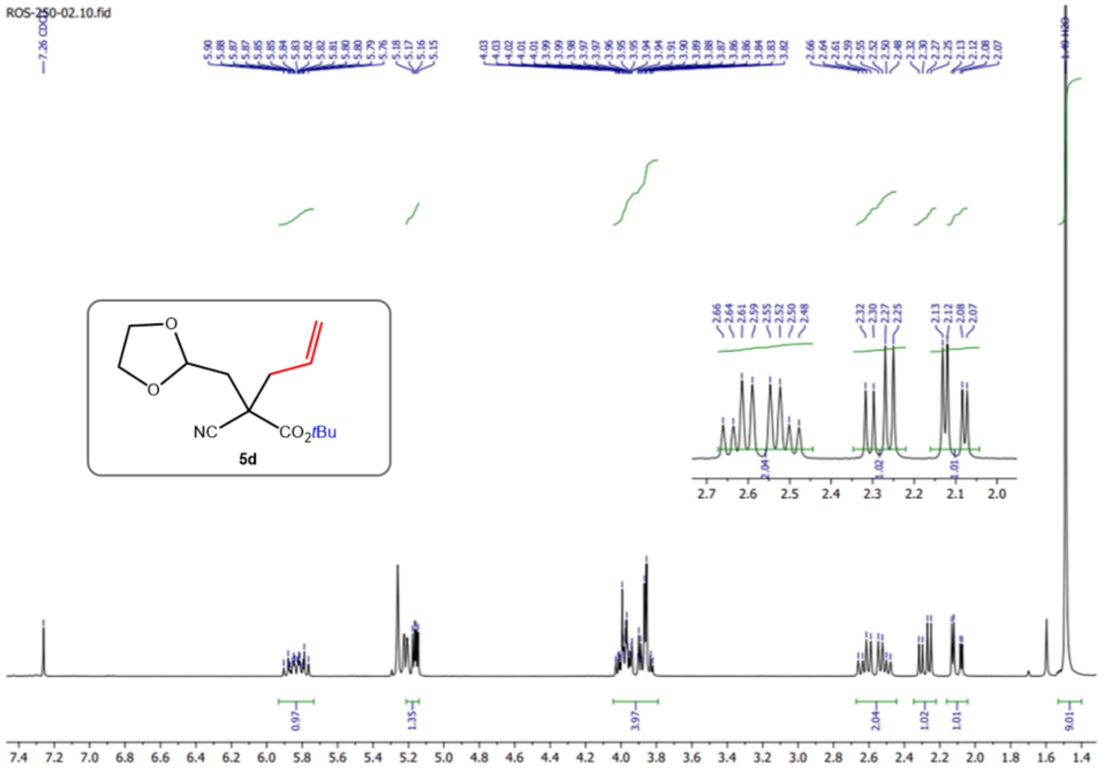

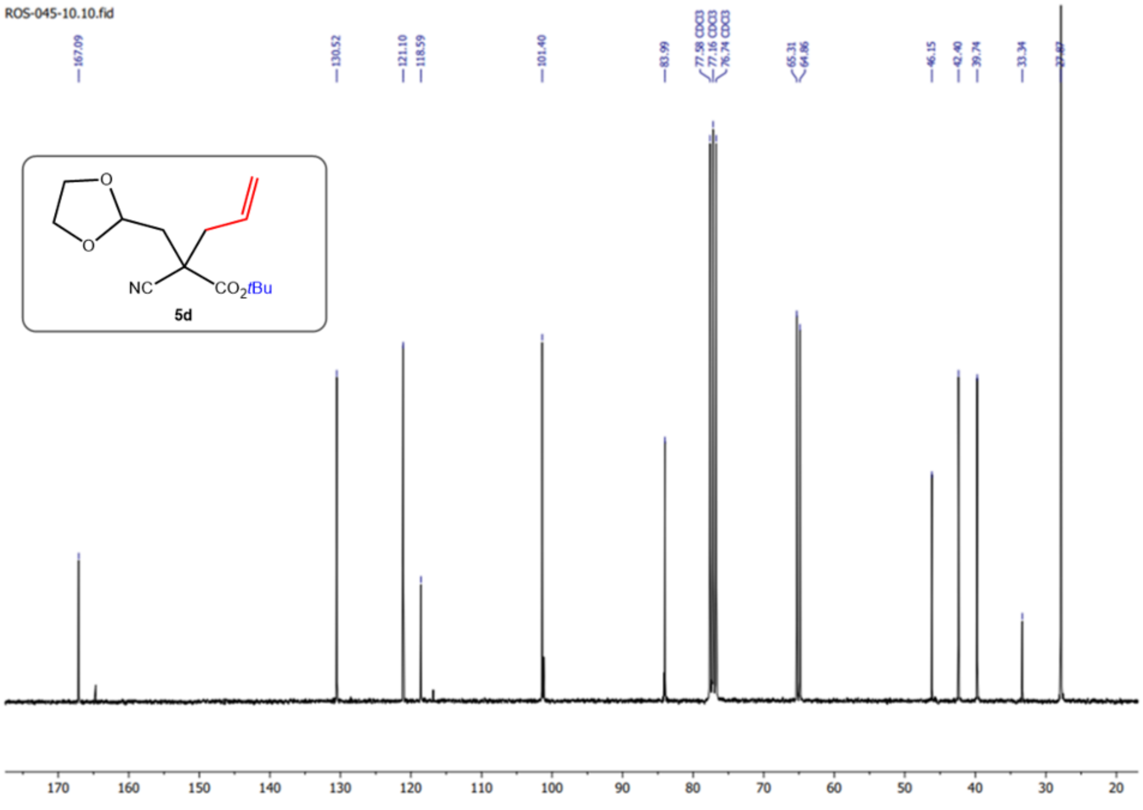

NMR spectra of **5e**

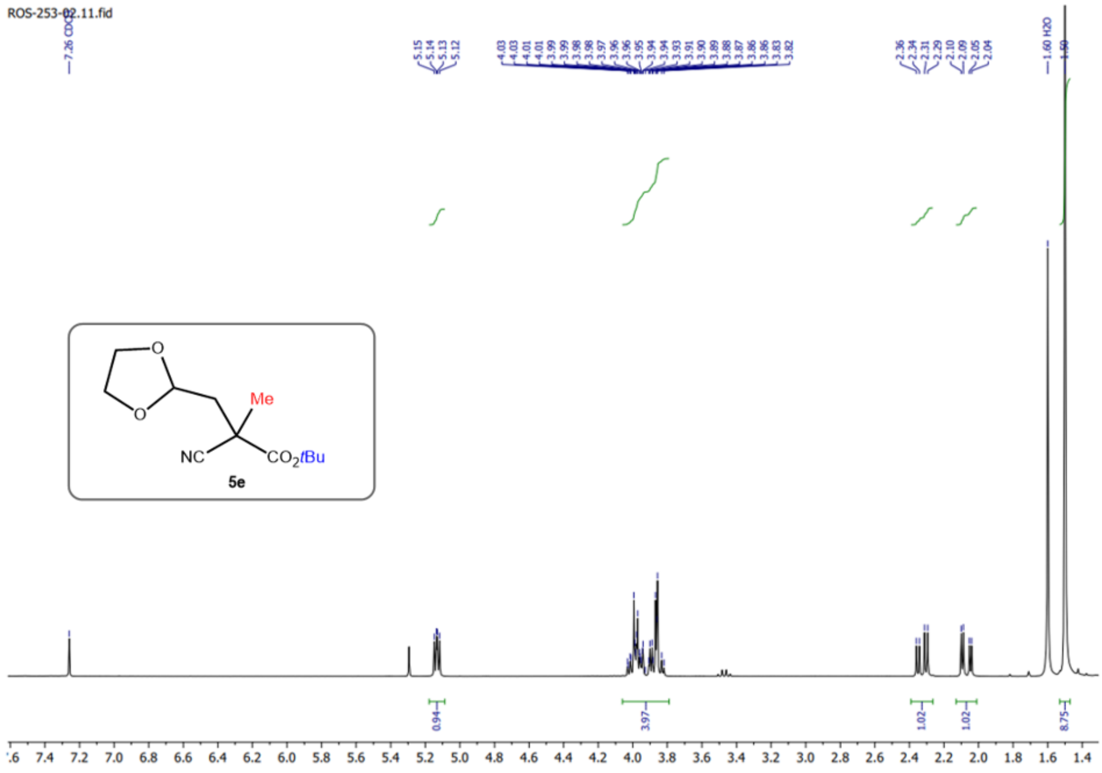

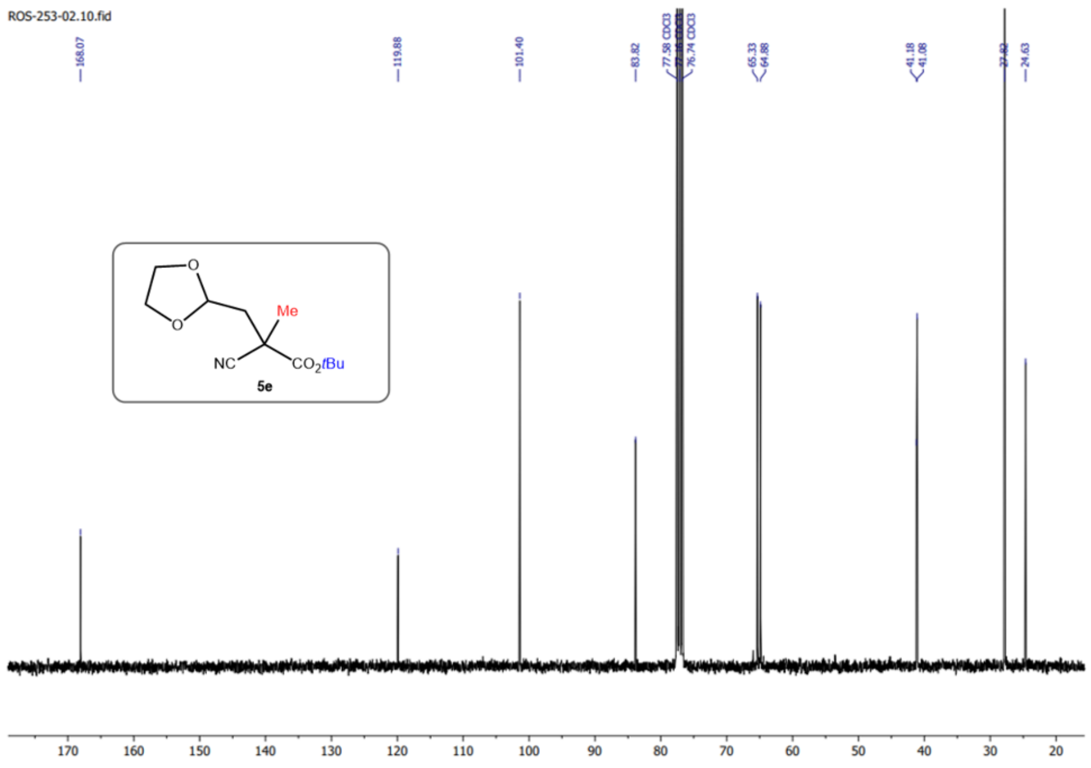

NMR spectra of **5f**

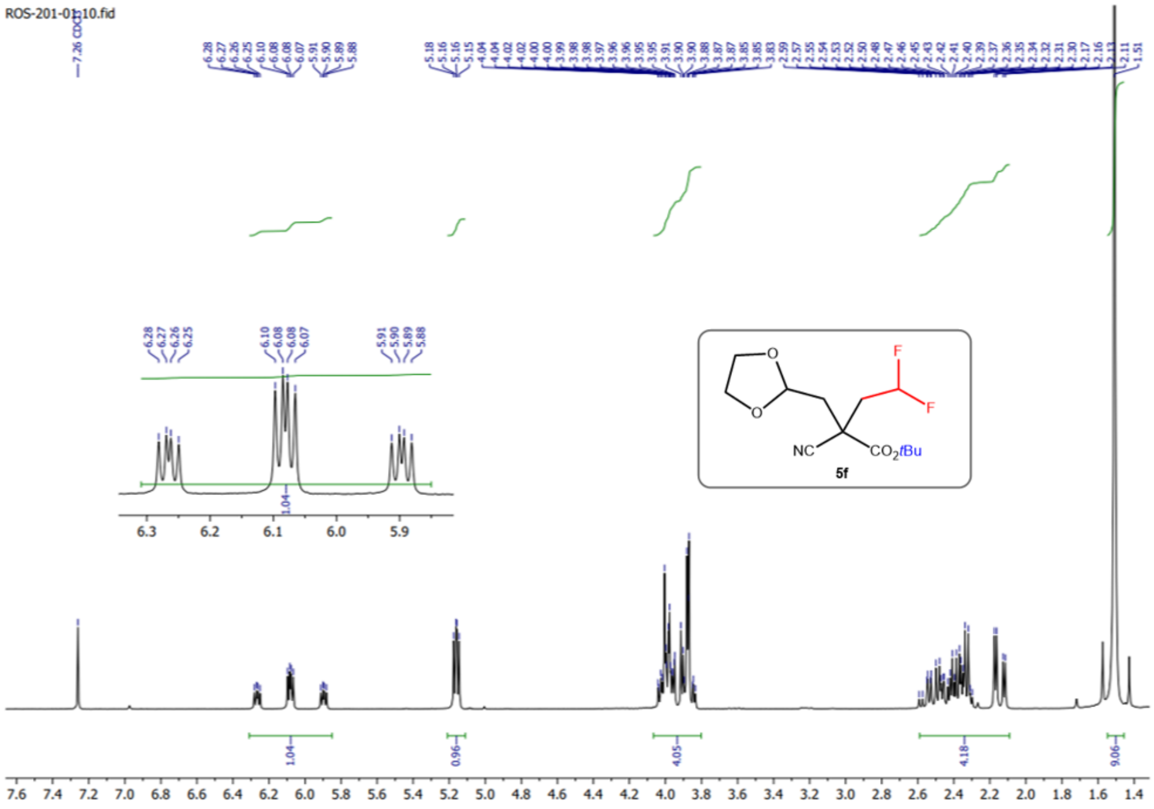

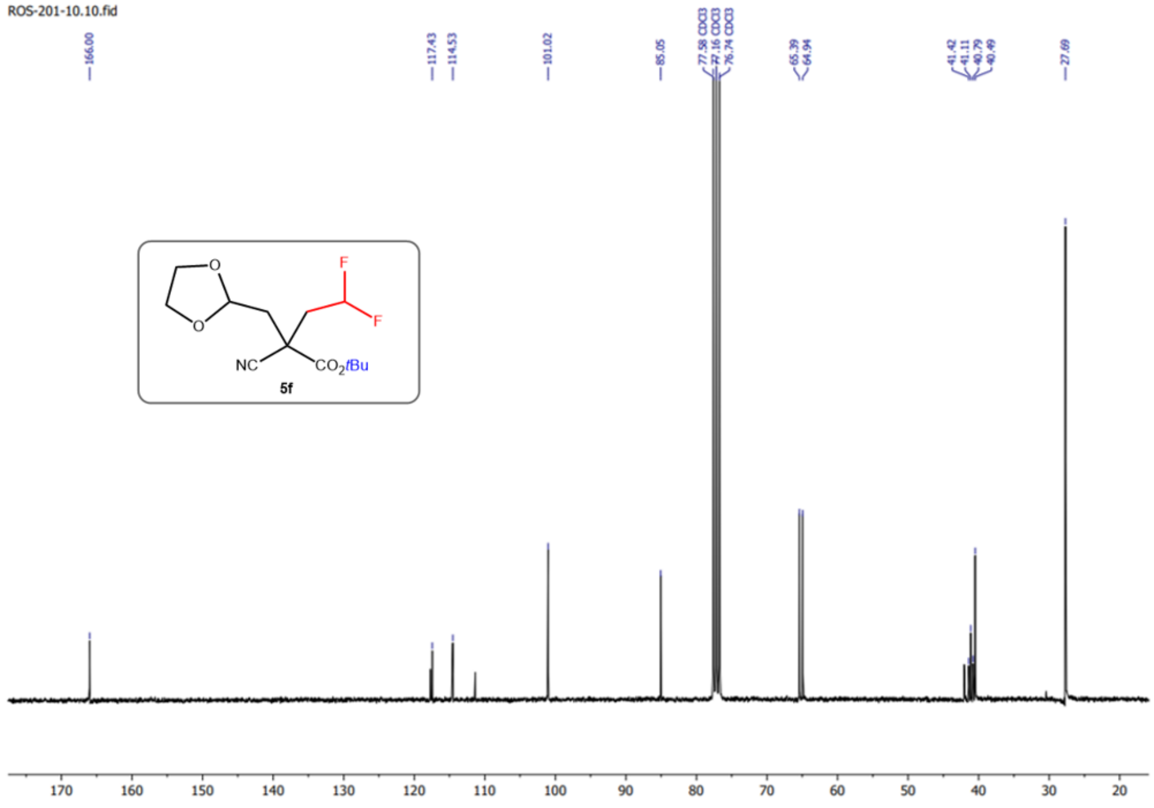

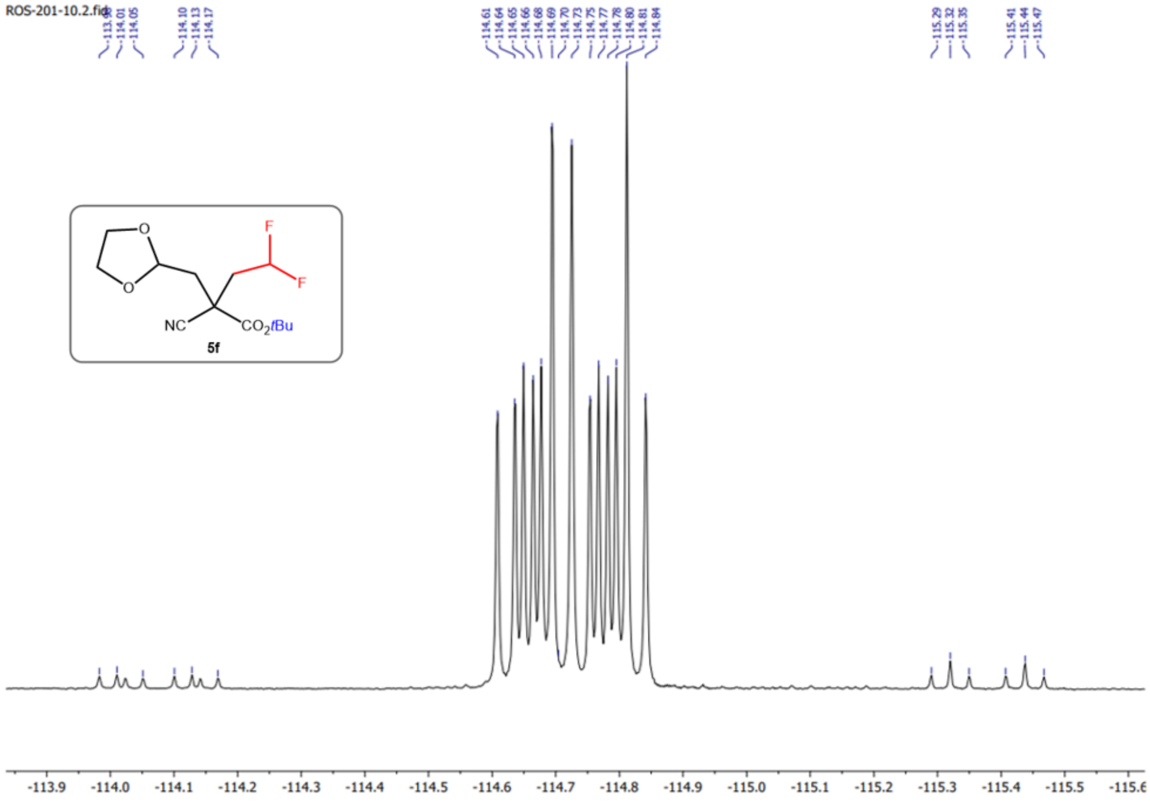

NMR spectra of **5g**

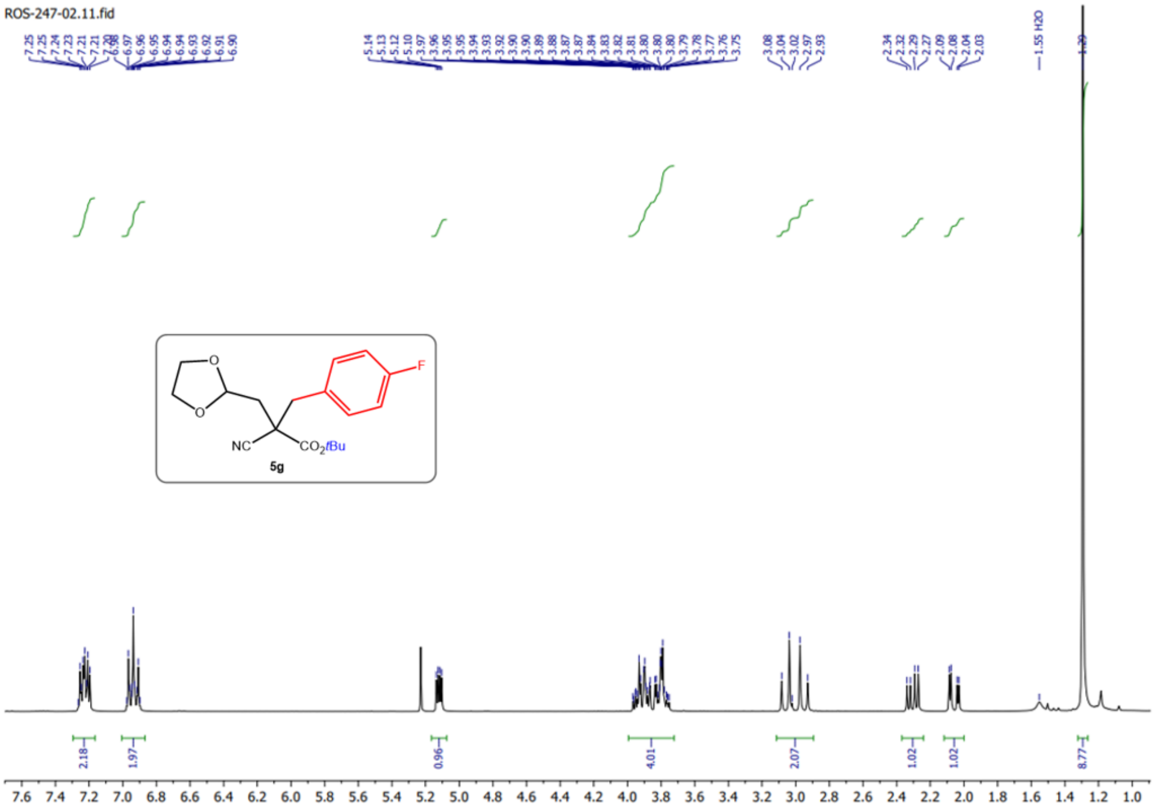

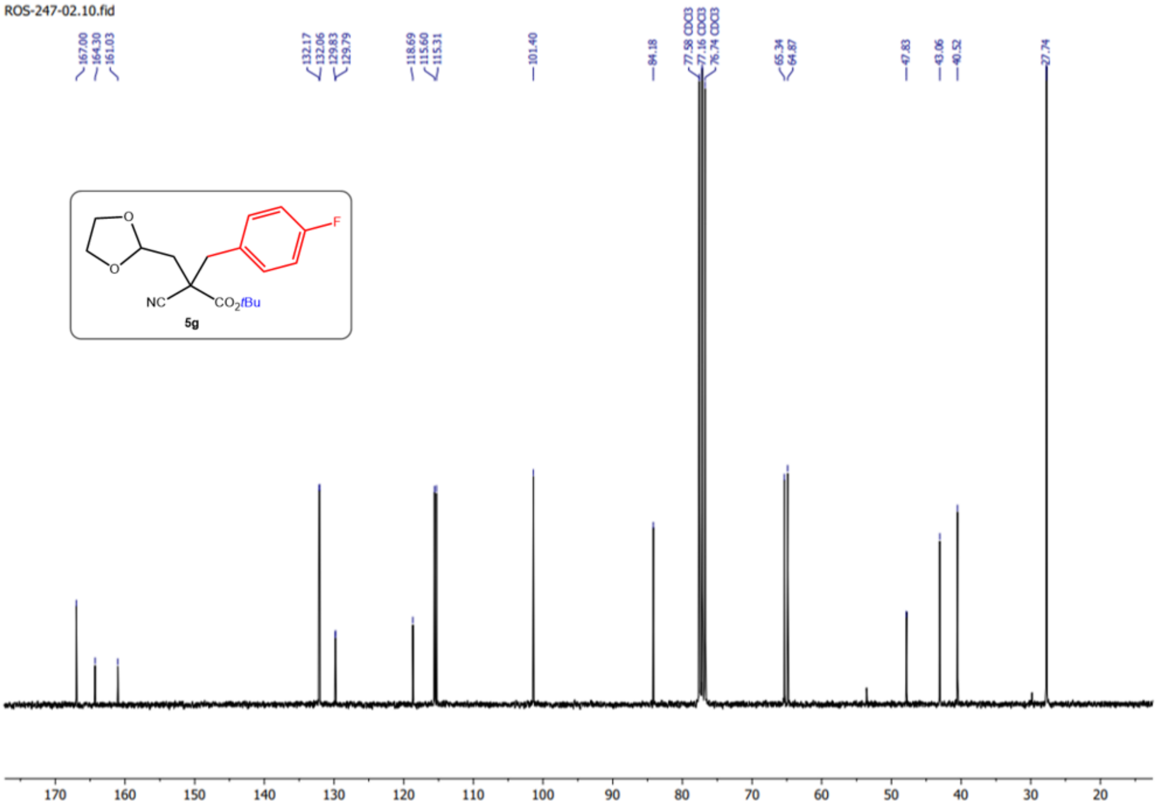
ROS-247-02.10.fid
167.00
164.30
161.03
132.17
132.06
129.83
129.79
118.69
115.60
115.31
101.40
84.18
77.58 CDCl3
77.16 CDCl3
76.74 CDCl3
65.34
64.87
47.83
43.06
40.52
27.74
NC
CO2tBu
F
5g
170
160
150
140
130
120
110
100
90
80
70
60
50
40
30
20

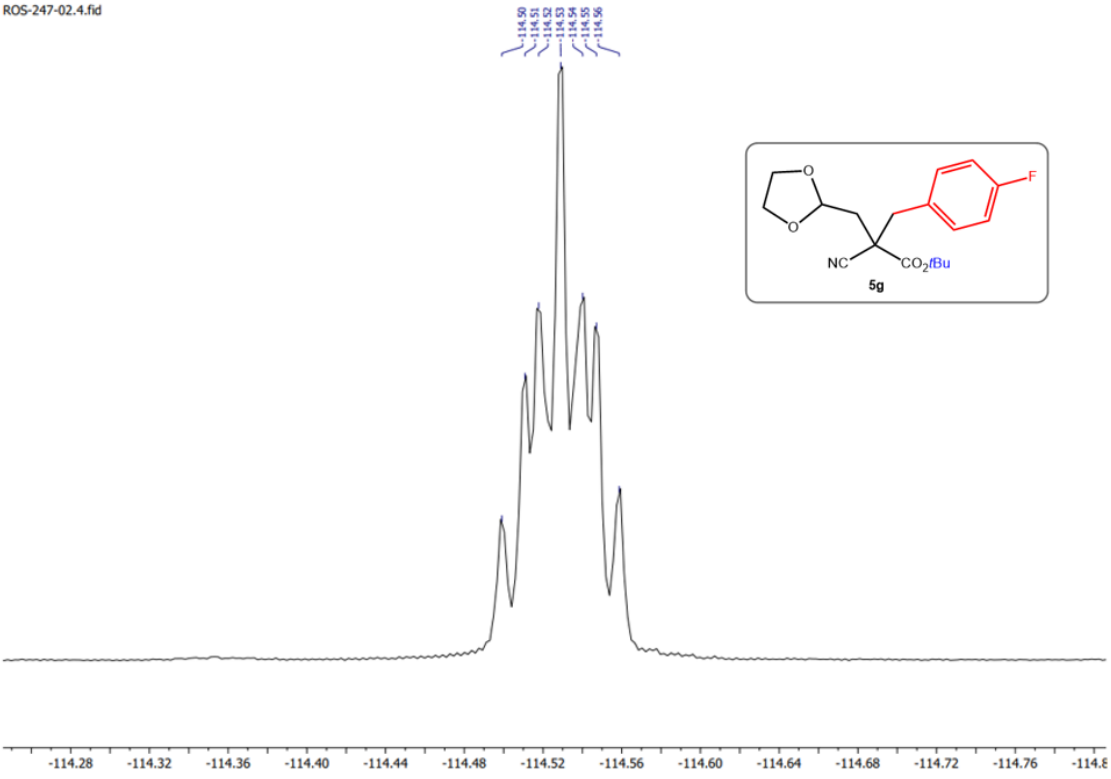
ROS-247-02.4.fid
-114.50
-114.51
-114.52
-114.53
-114.54
-114.55
-114.56
NC
CO2tBu
F
5g
-114.28
-114.32
-114.36
-114.40
-114.44
-114.48
-114.52
-114.56
-114.60
-114.64
-114.68
-114.72
-114.76

NMR spectra of **5h**

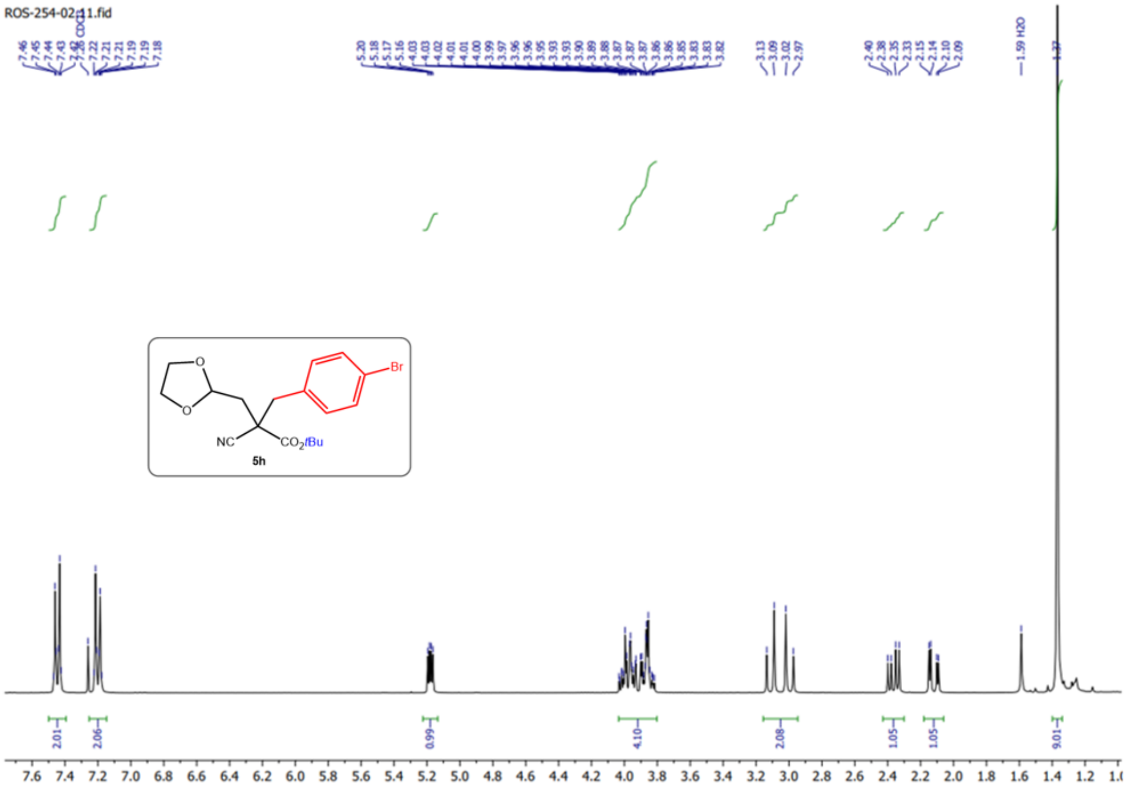

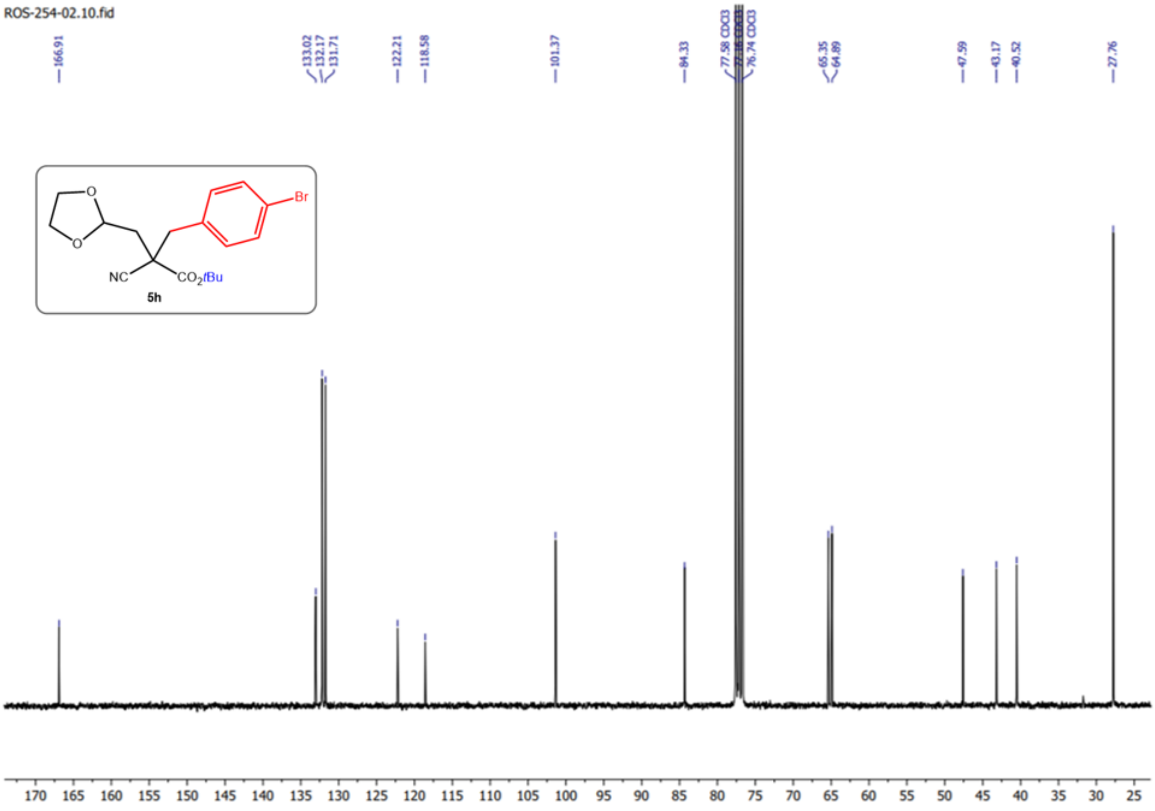

NMR spectra of **5i**

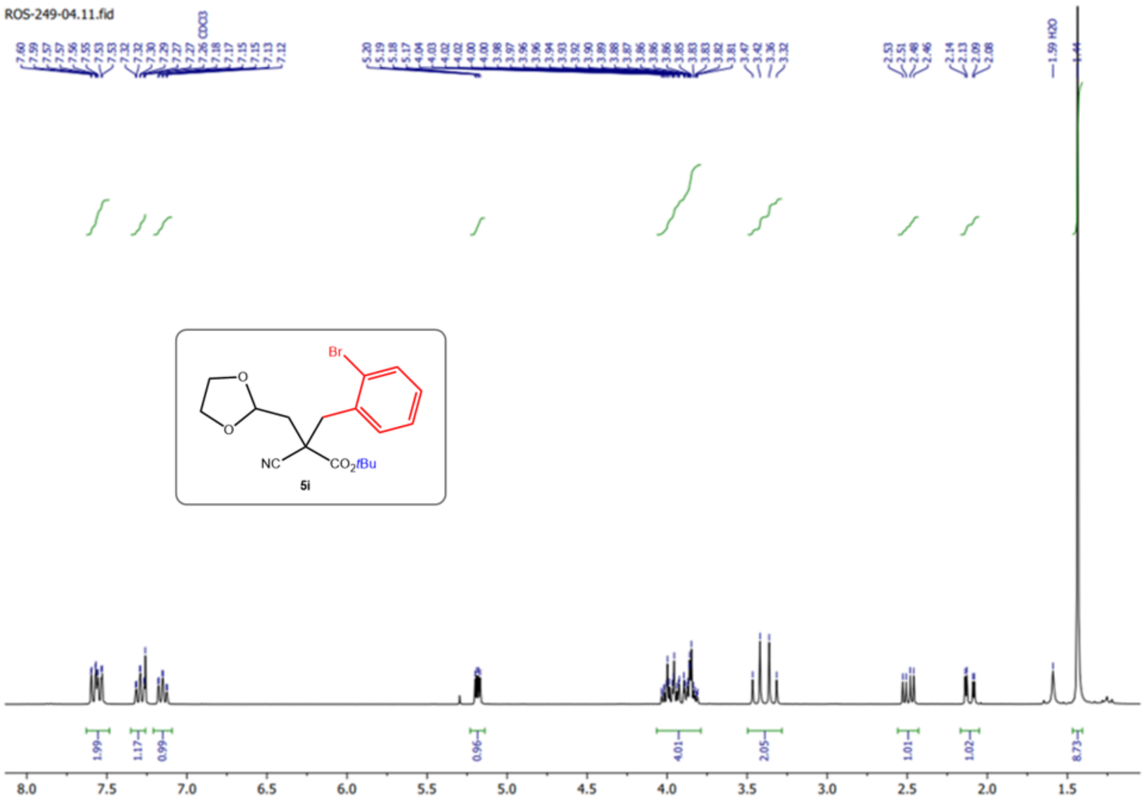

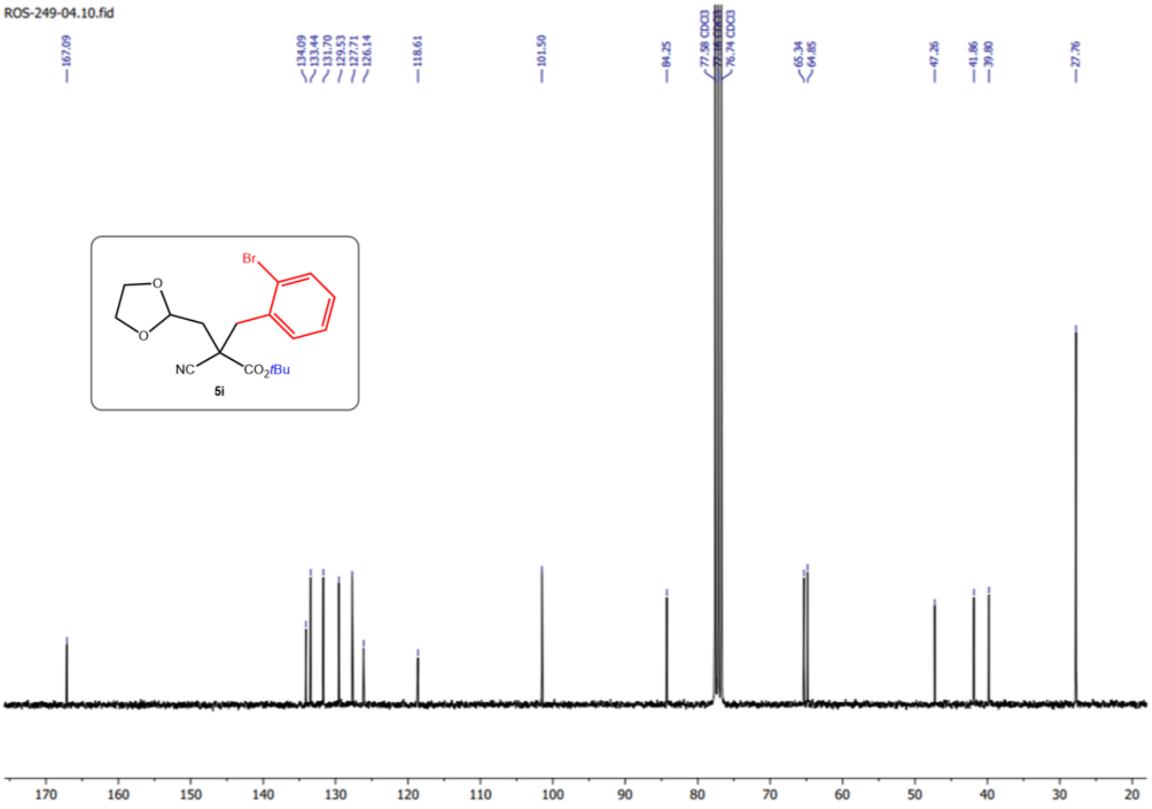

NMR spectra of **5j**

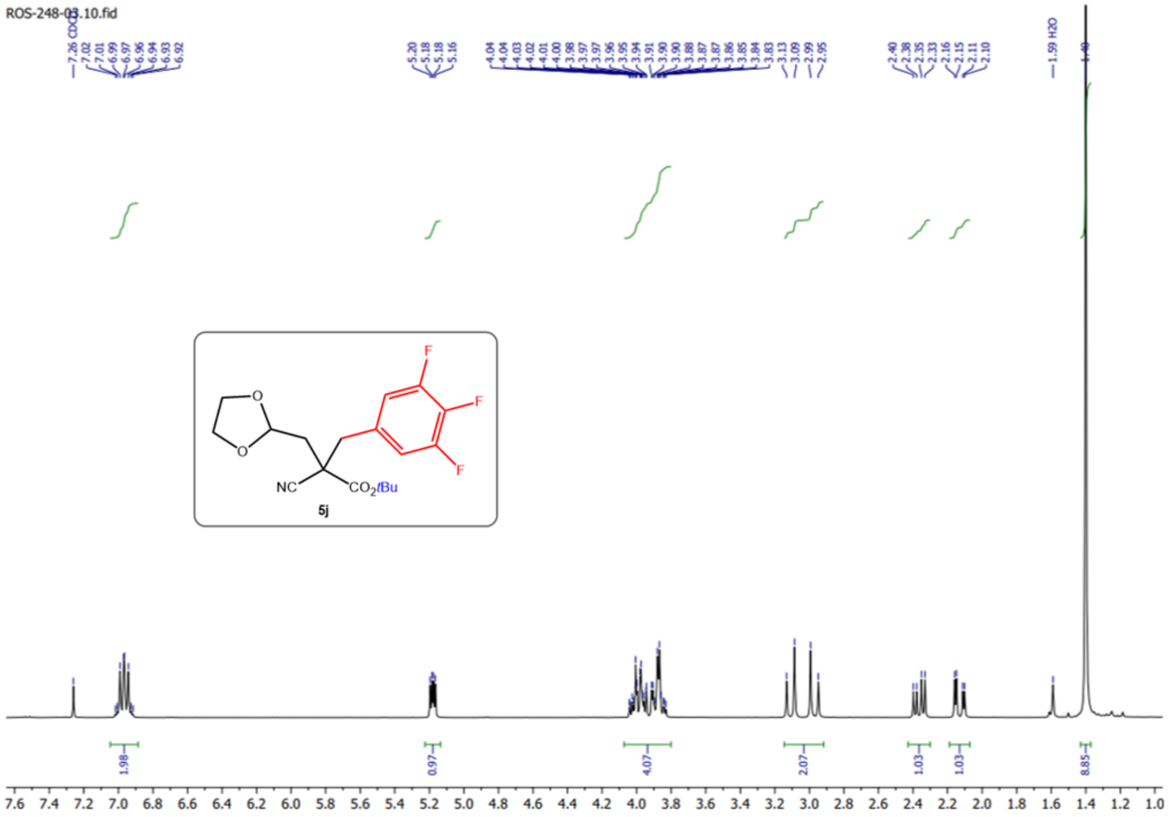

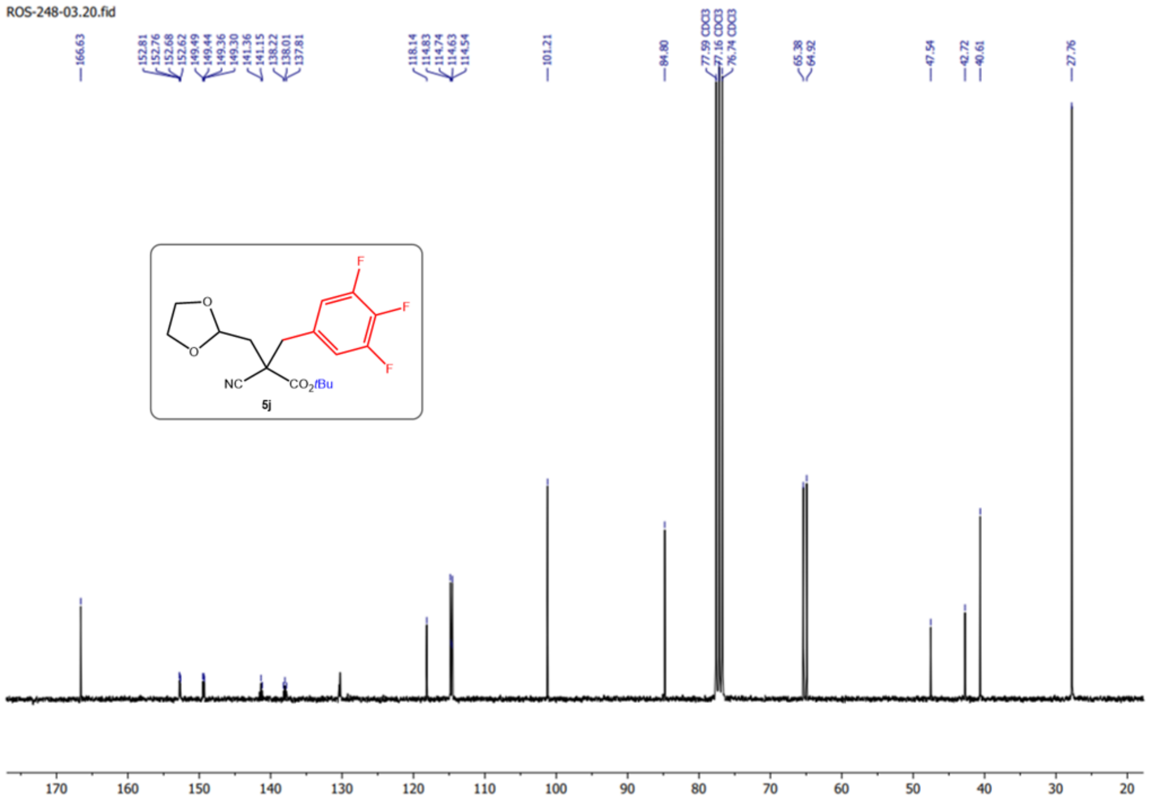

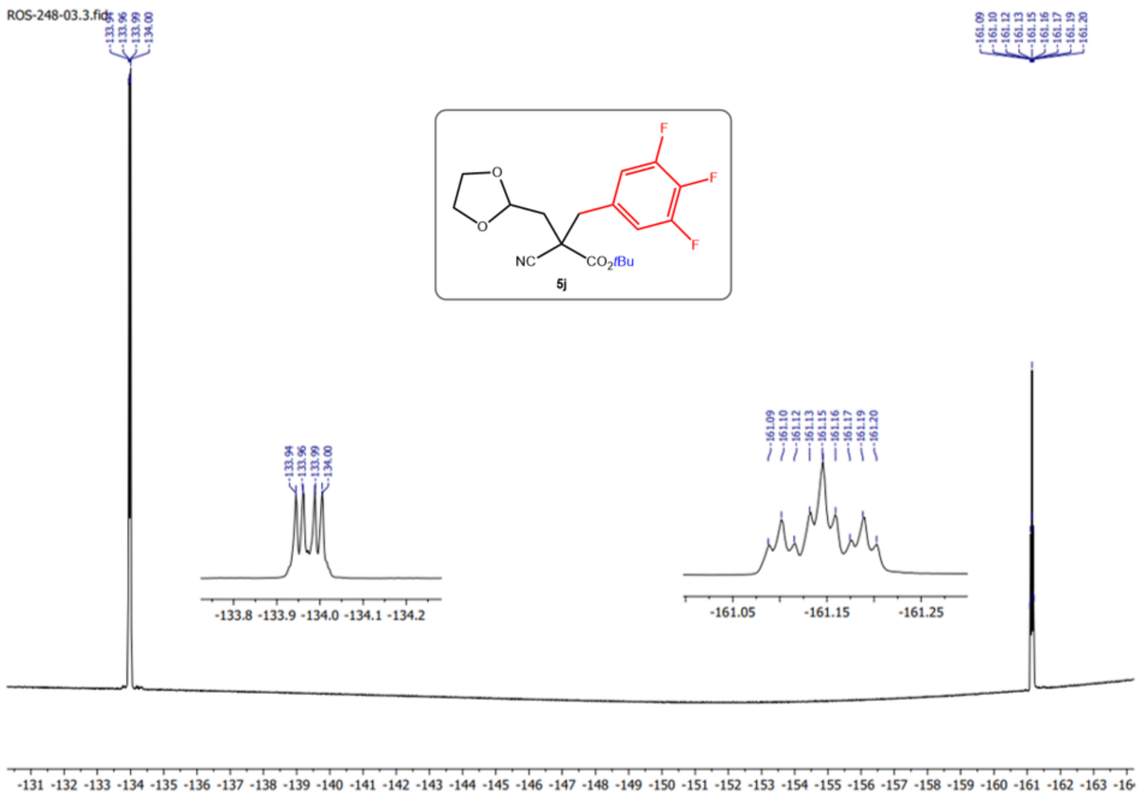

NMR spectra of **5k**

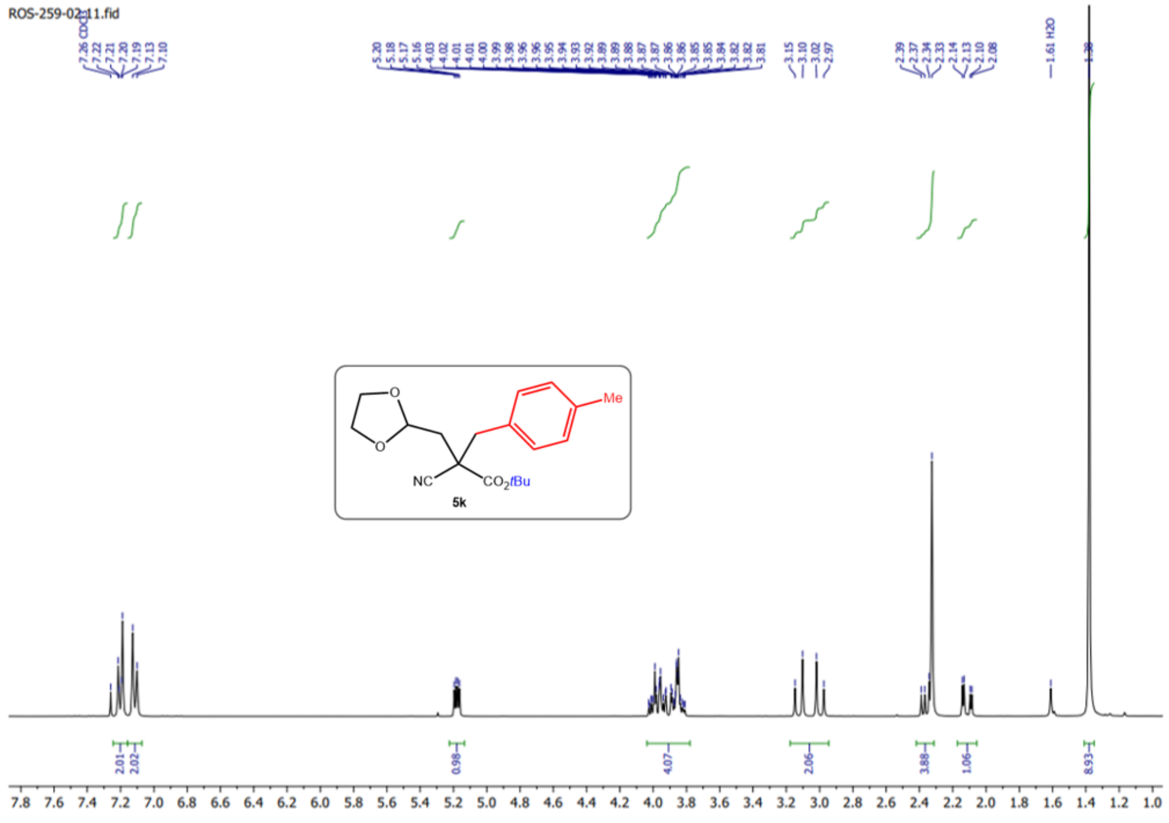

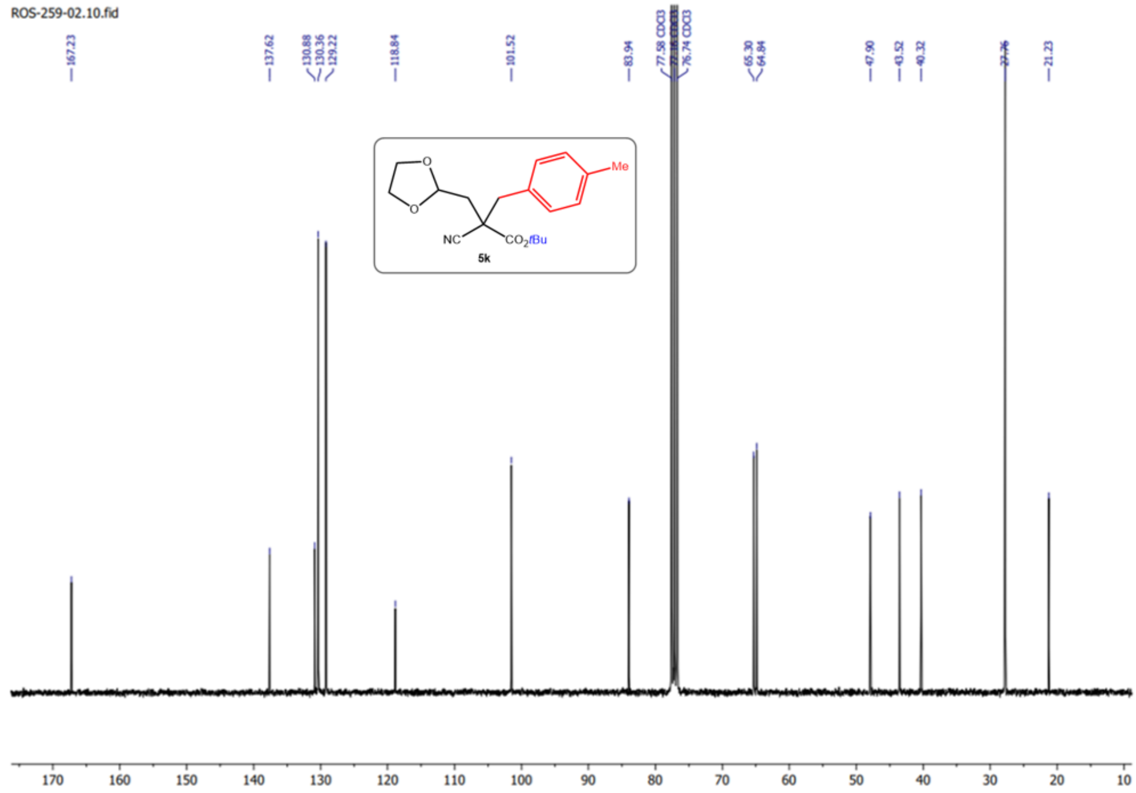

NMR spectra of **5l**

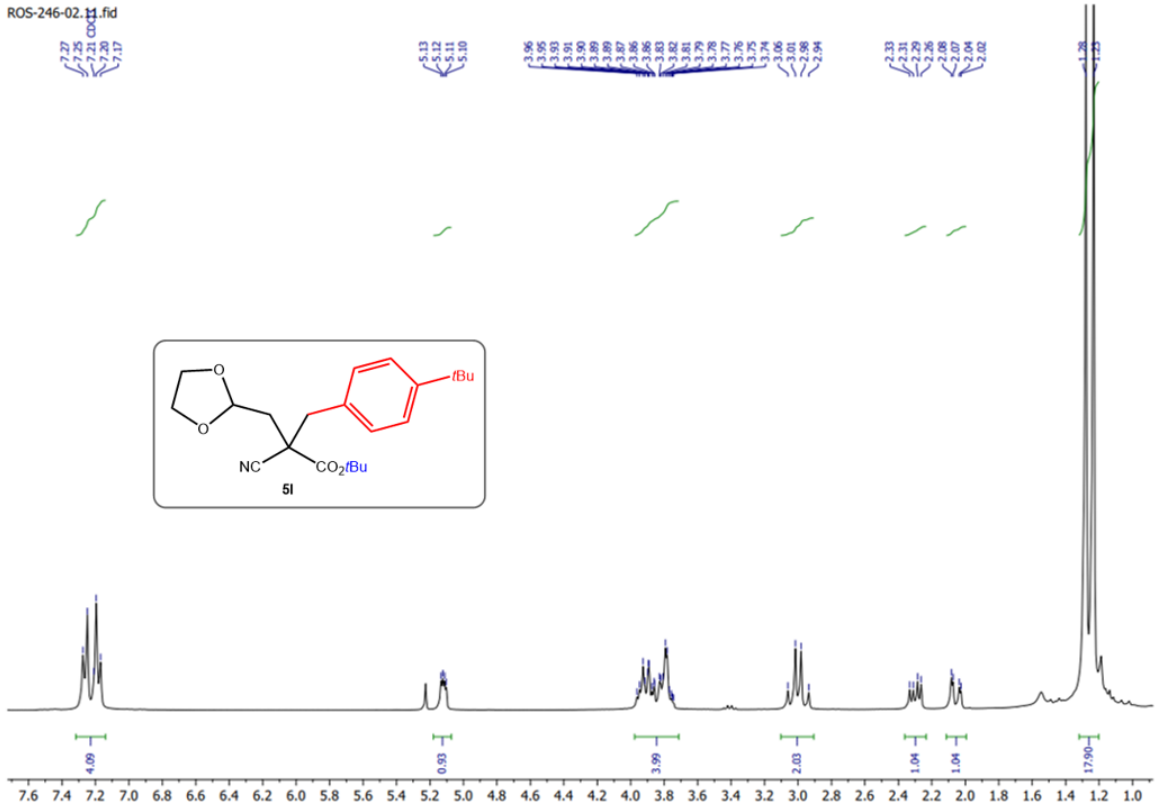

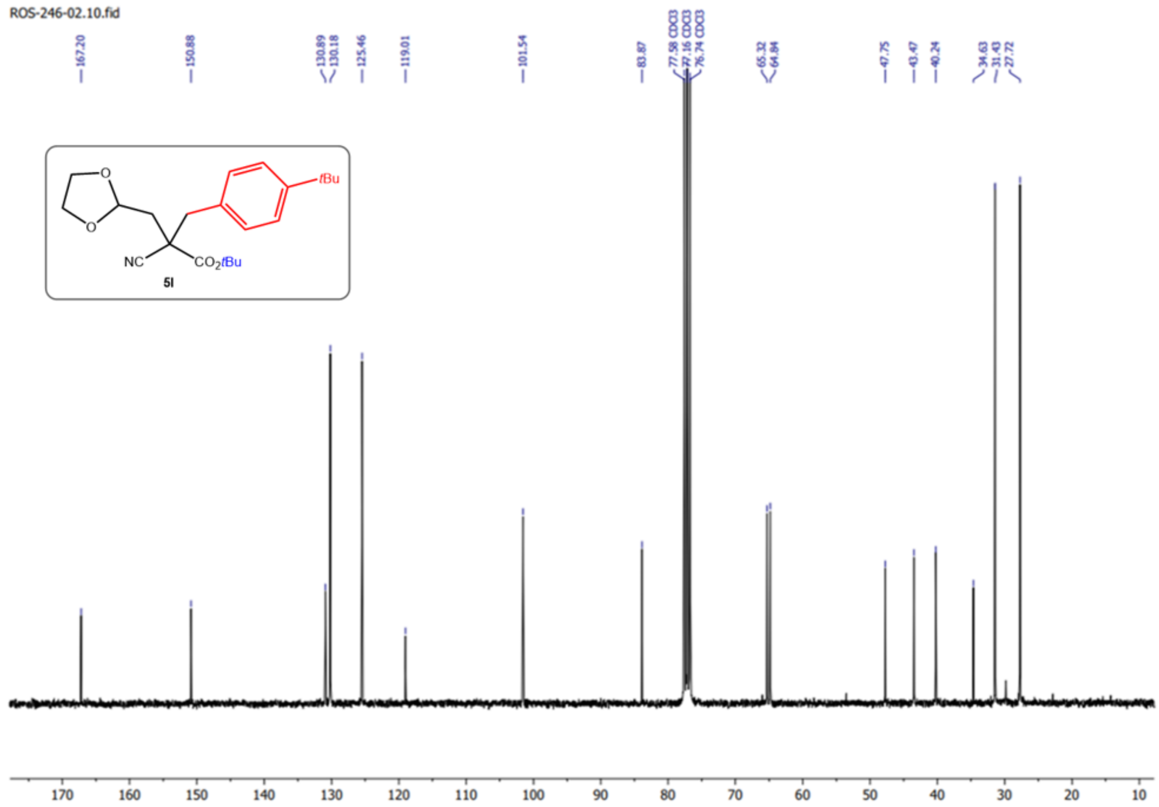

NMR spectra of **5m**

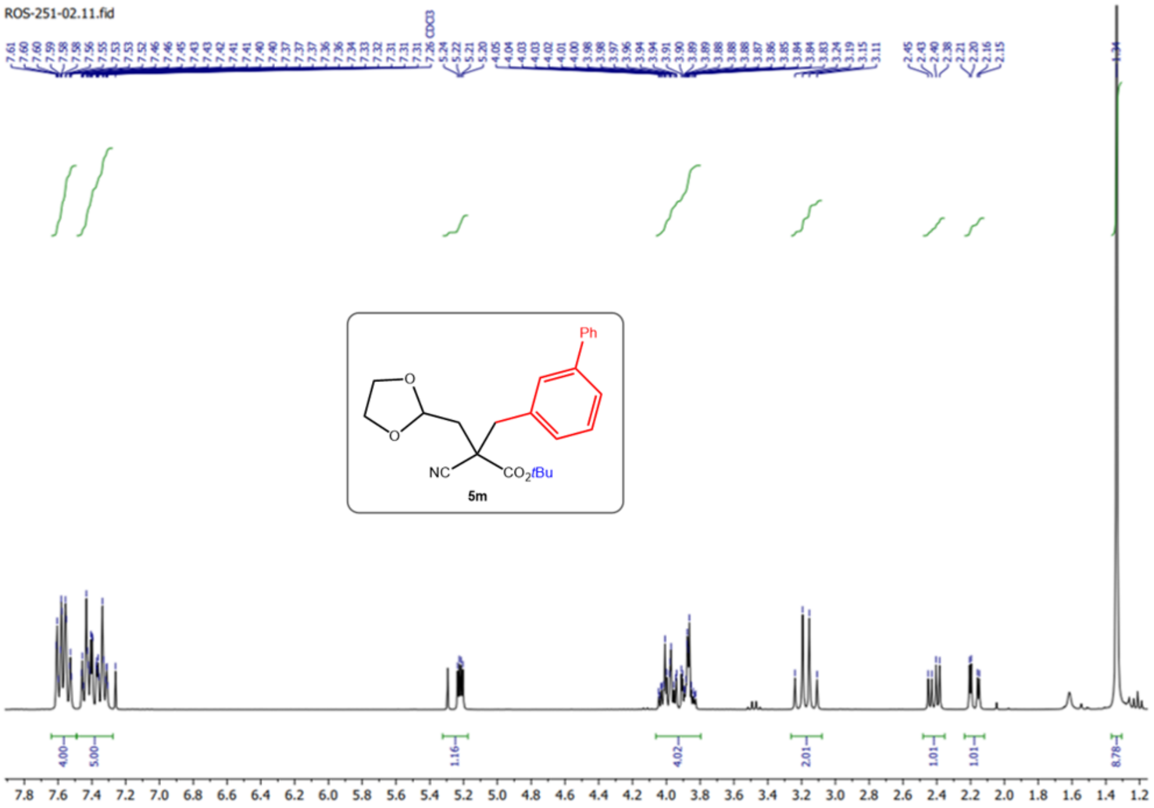

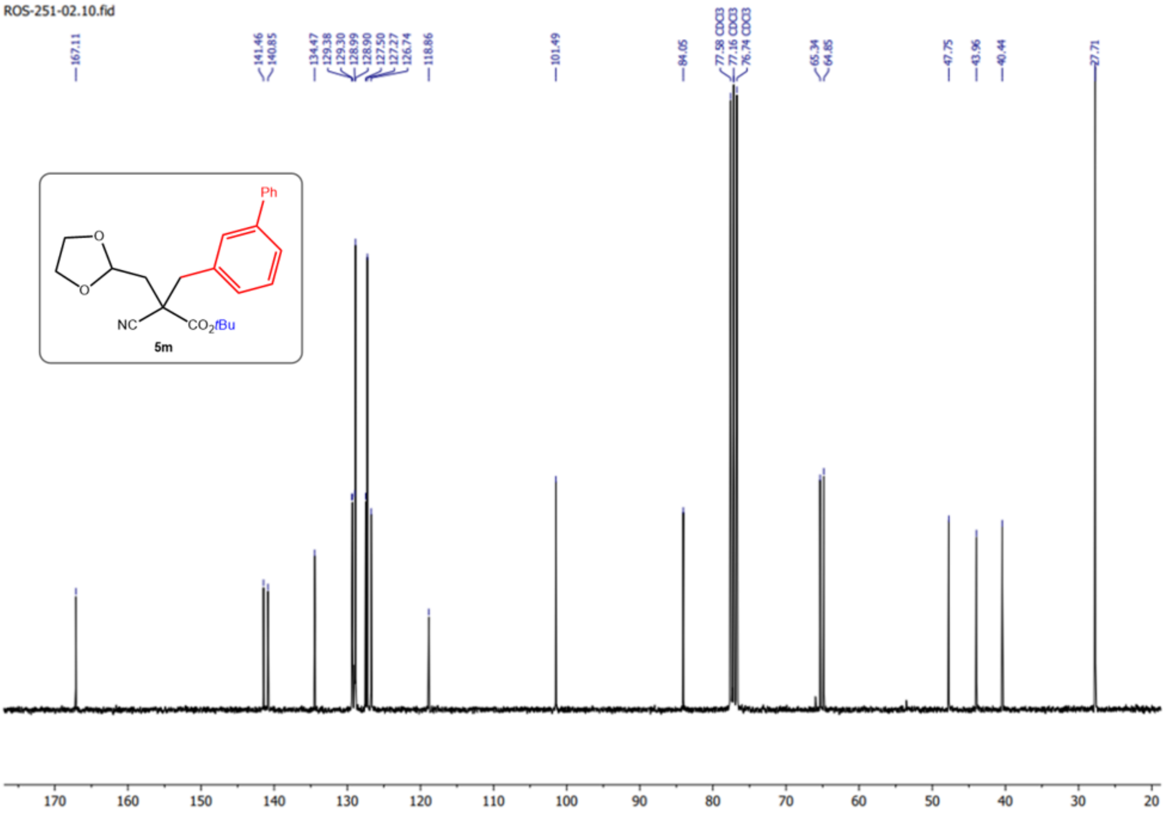

NMR spectra of **5n**

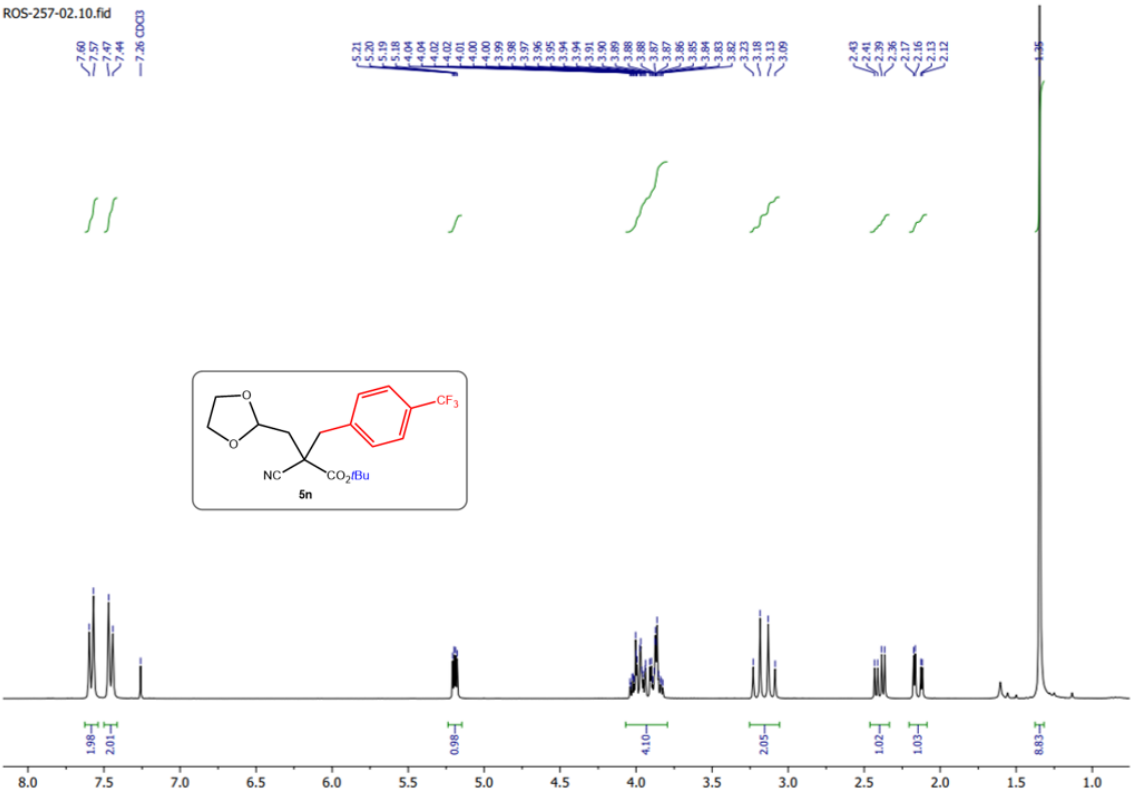

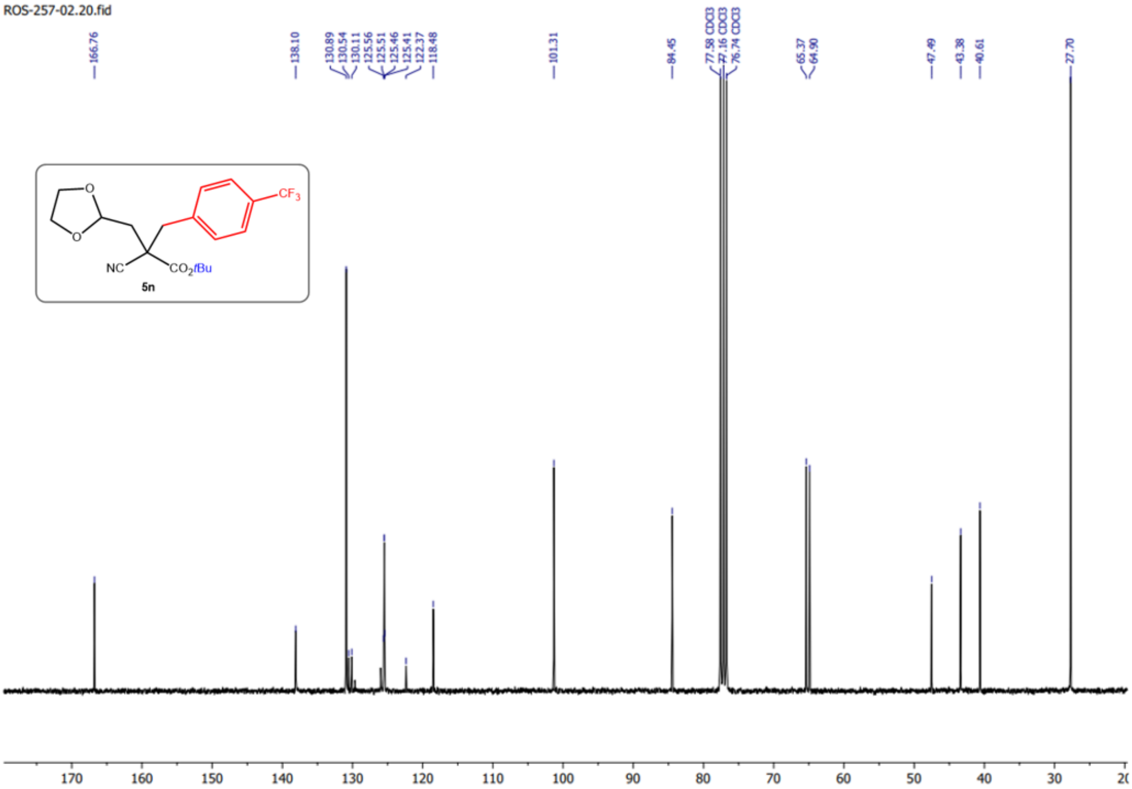
ROS-257-02.20.fid
166.76
138.10
130.89
130.54
130.11
125.56
125.51
125.46
125.41
122.37
118.48
101.31
84.45
77.58 CDCl3
77.16 CDCl3
76.74 CDCl3
65.37
64.90
47.49
43.38
40.61
27.70
NC
CO2tBu
CF3
5n

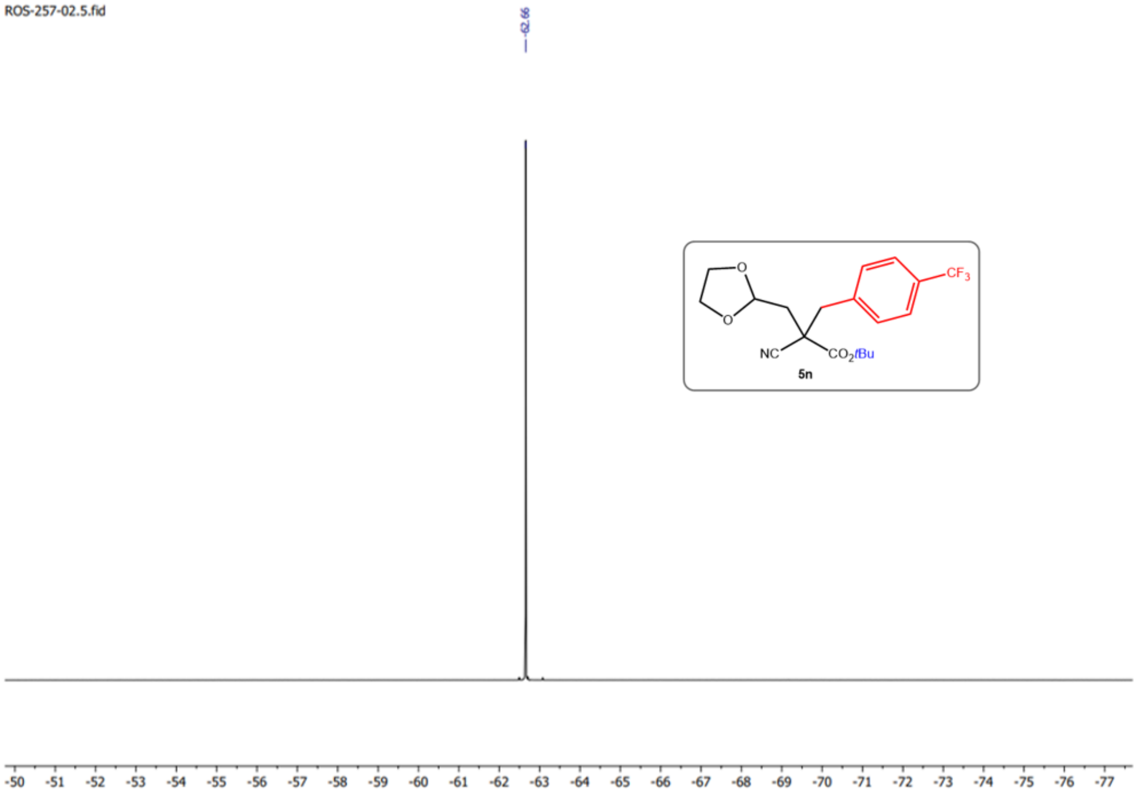
ROS-257-02.5.fid
-62.66
NC
CO2tBu
CF3
5n

NMR spectra of **5o**

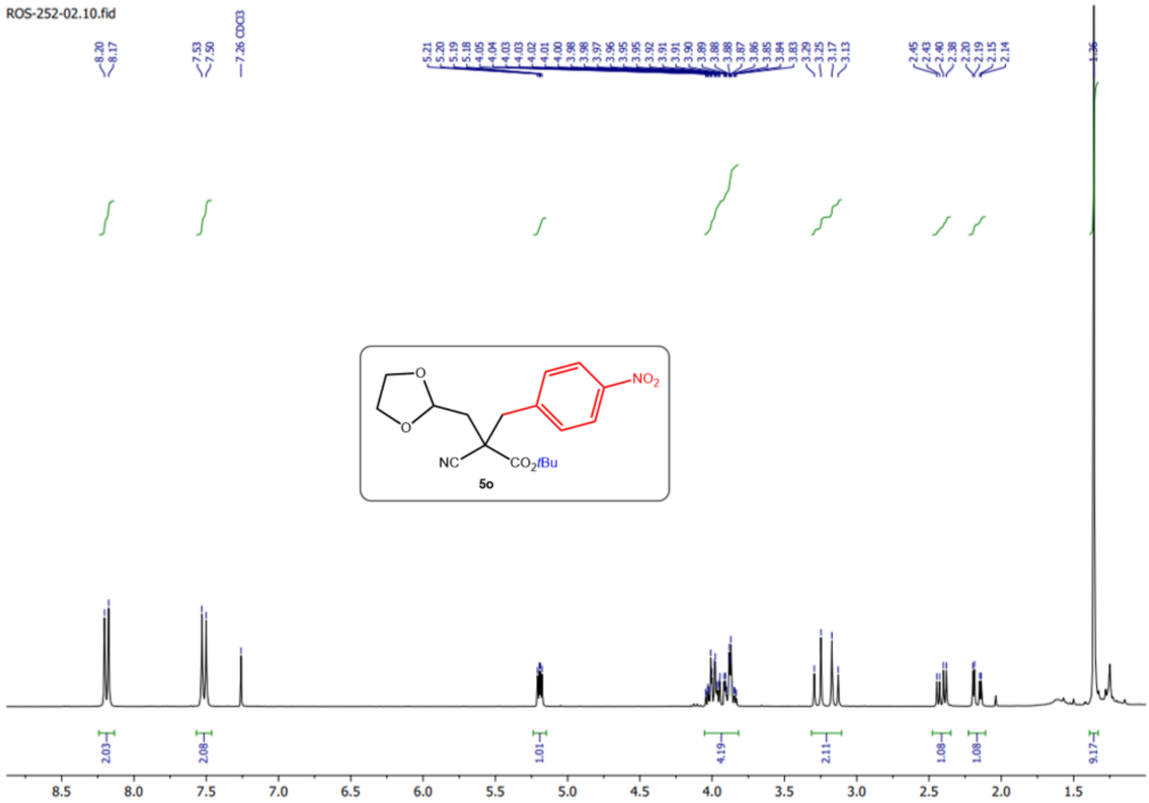

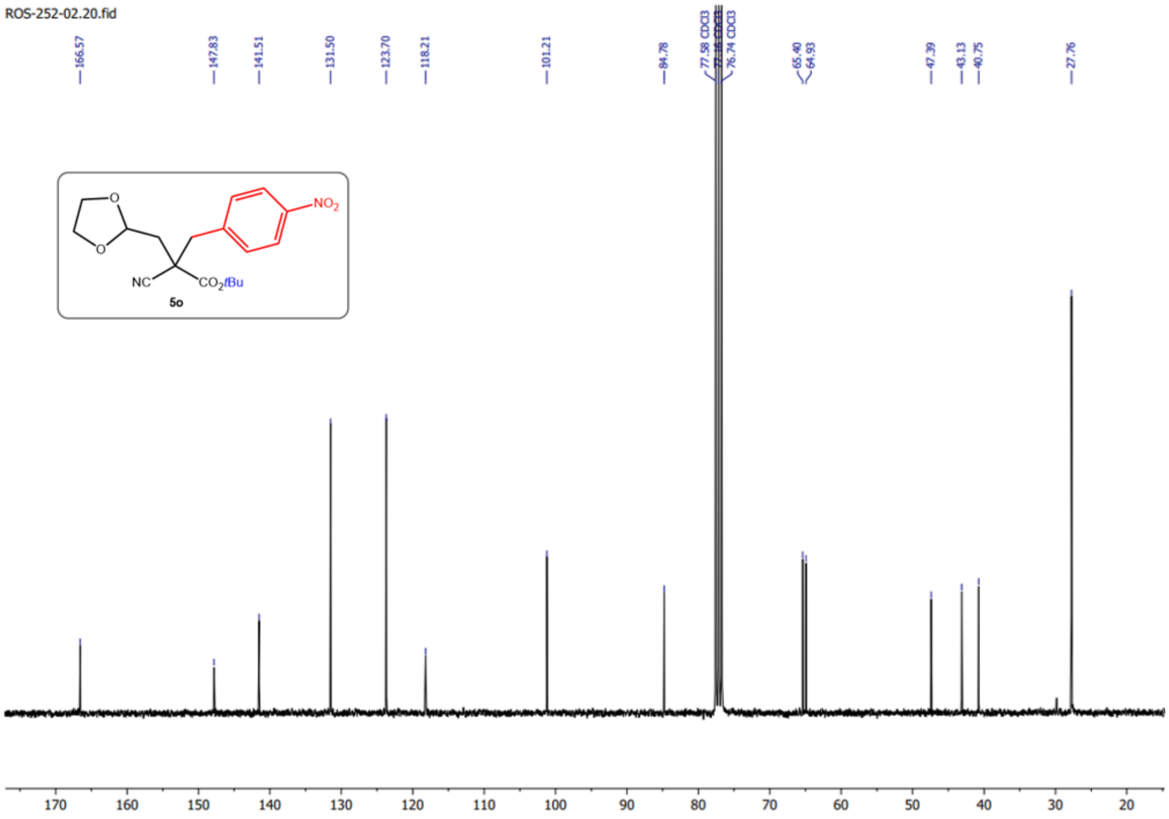

NMR spectra of **5p**

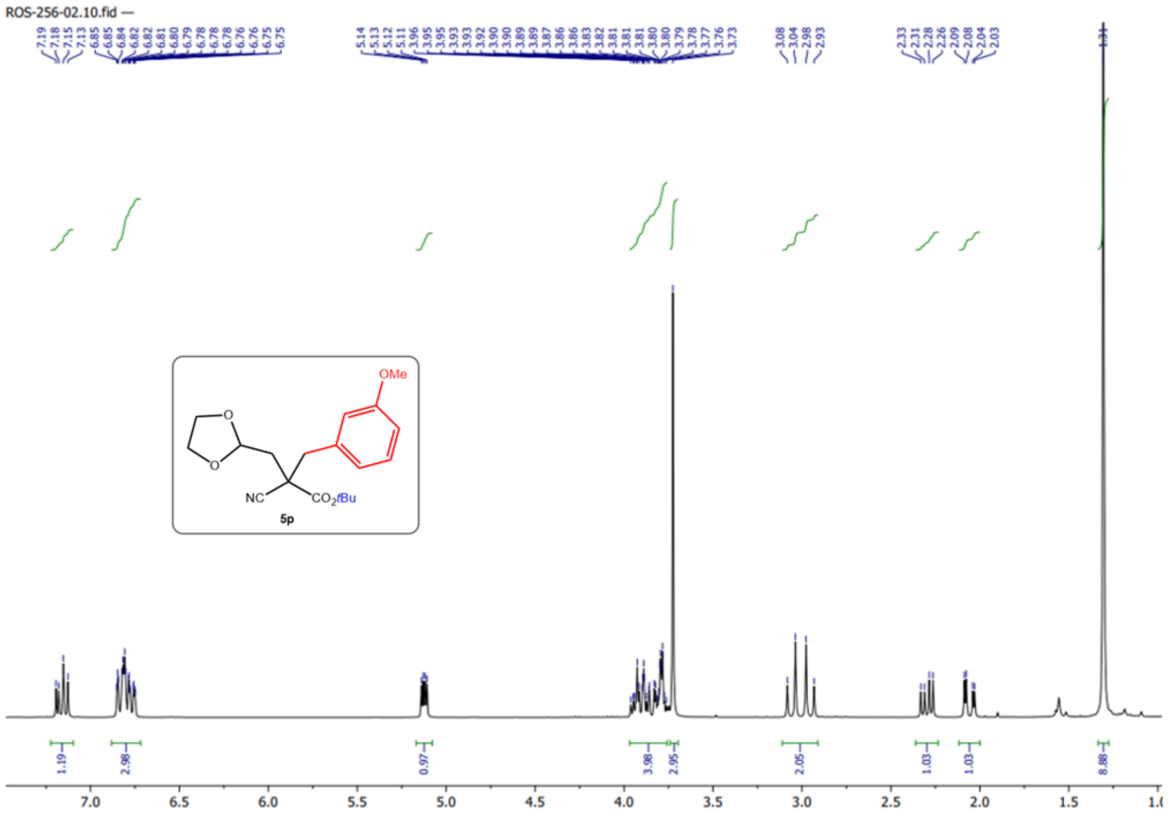

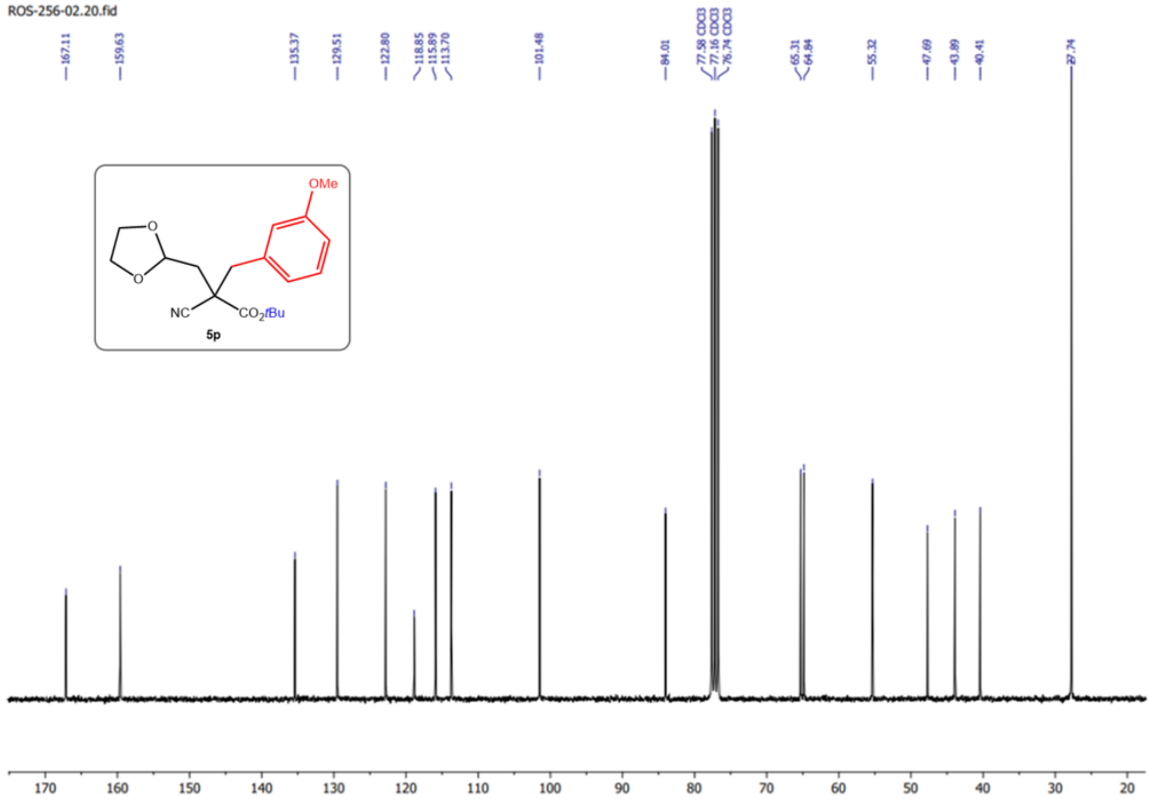

NMR spectra of **5q**

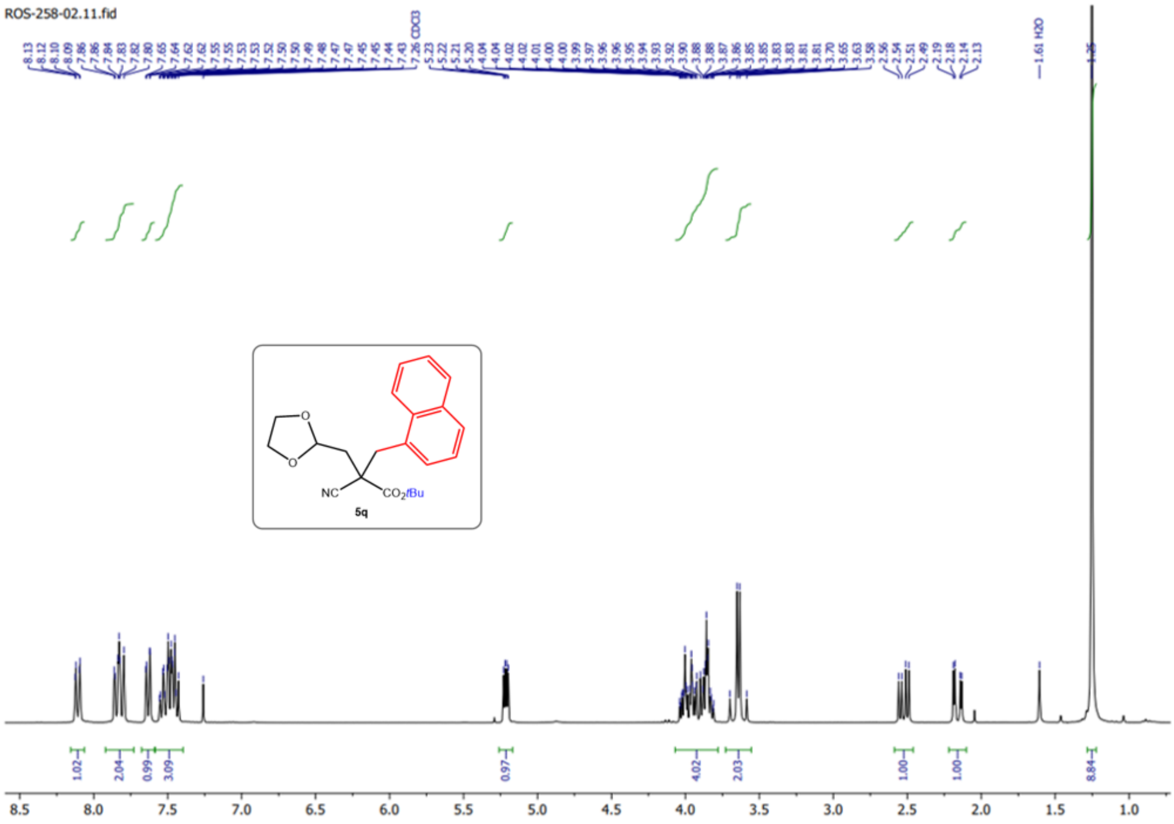

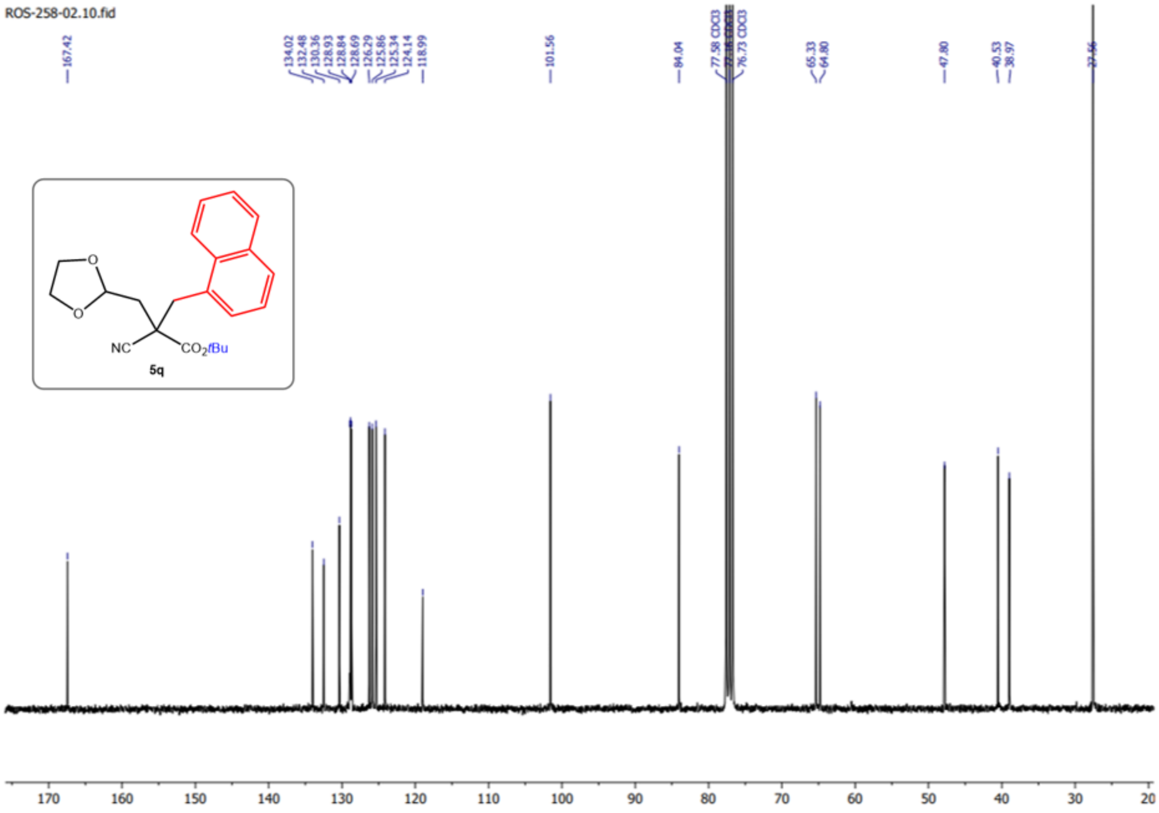

NMR spectra of **6**

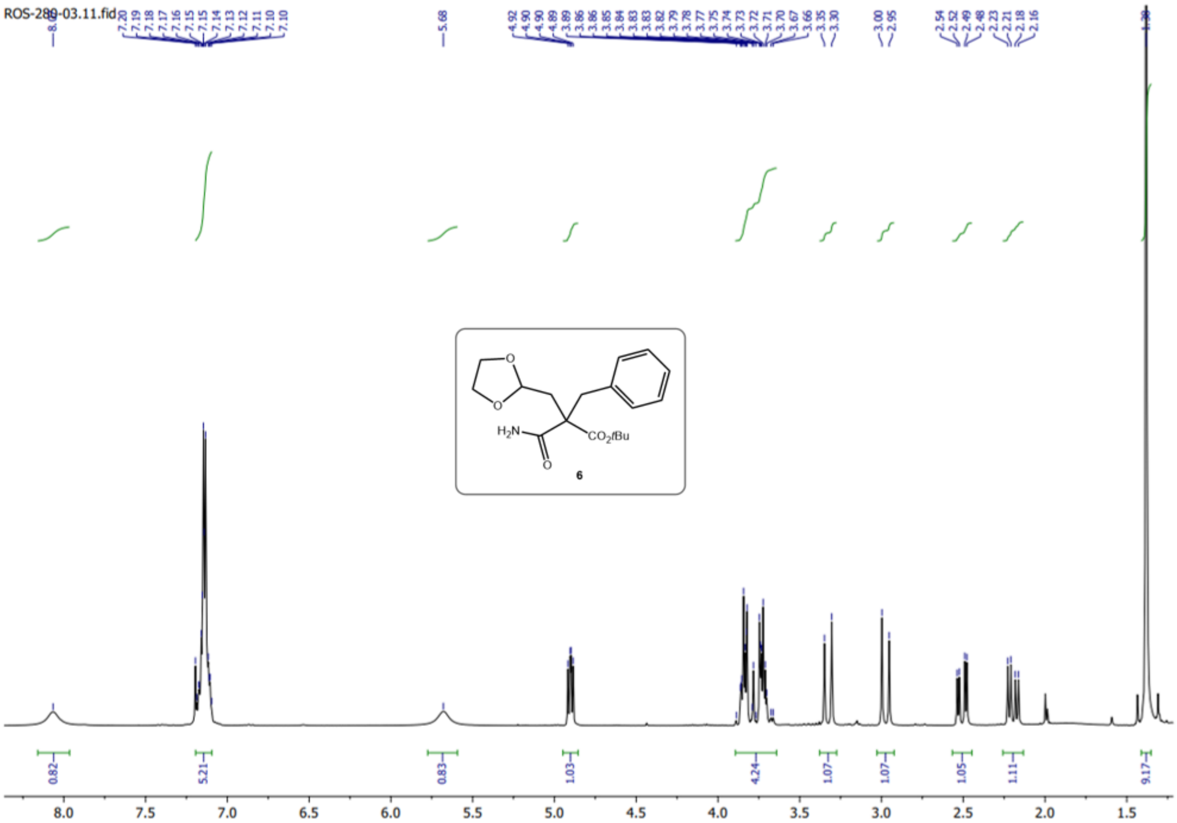

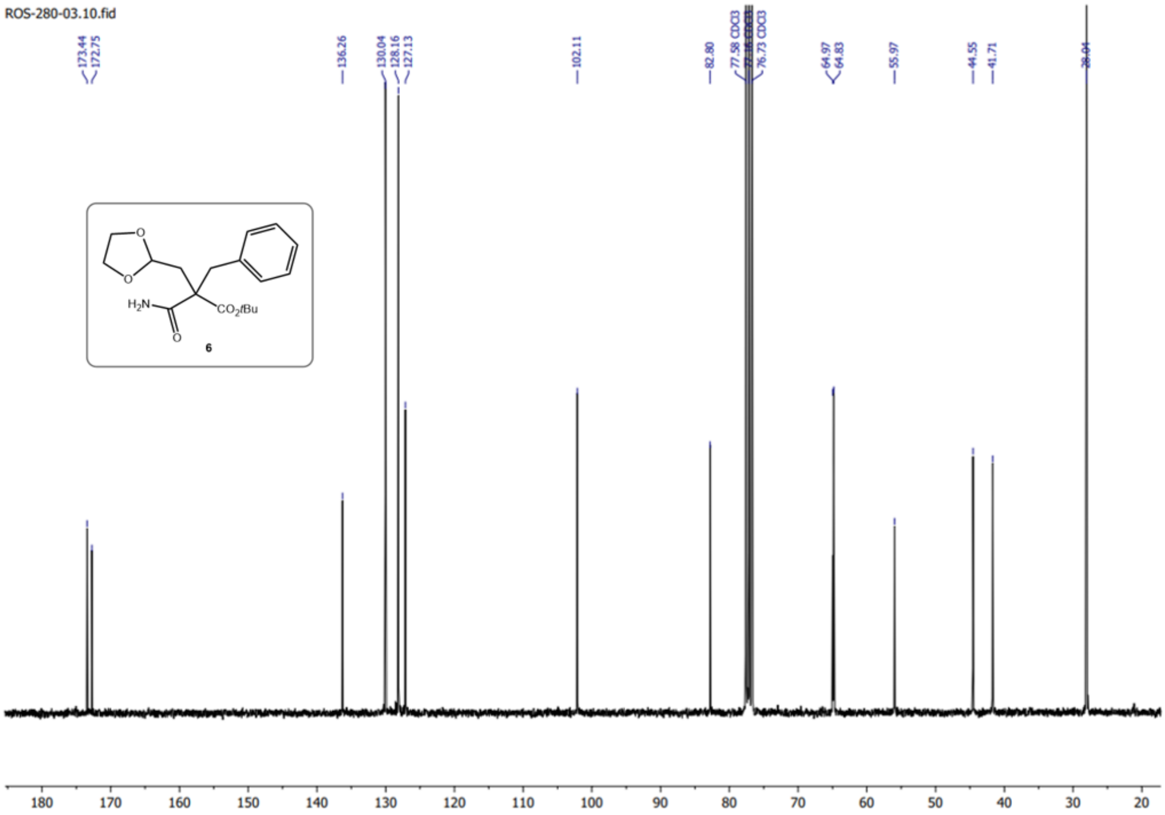

NMR spectra of **7**

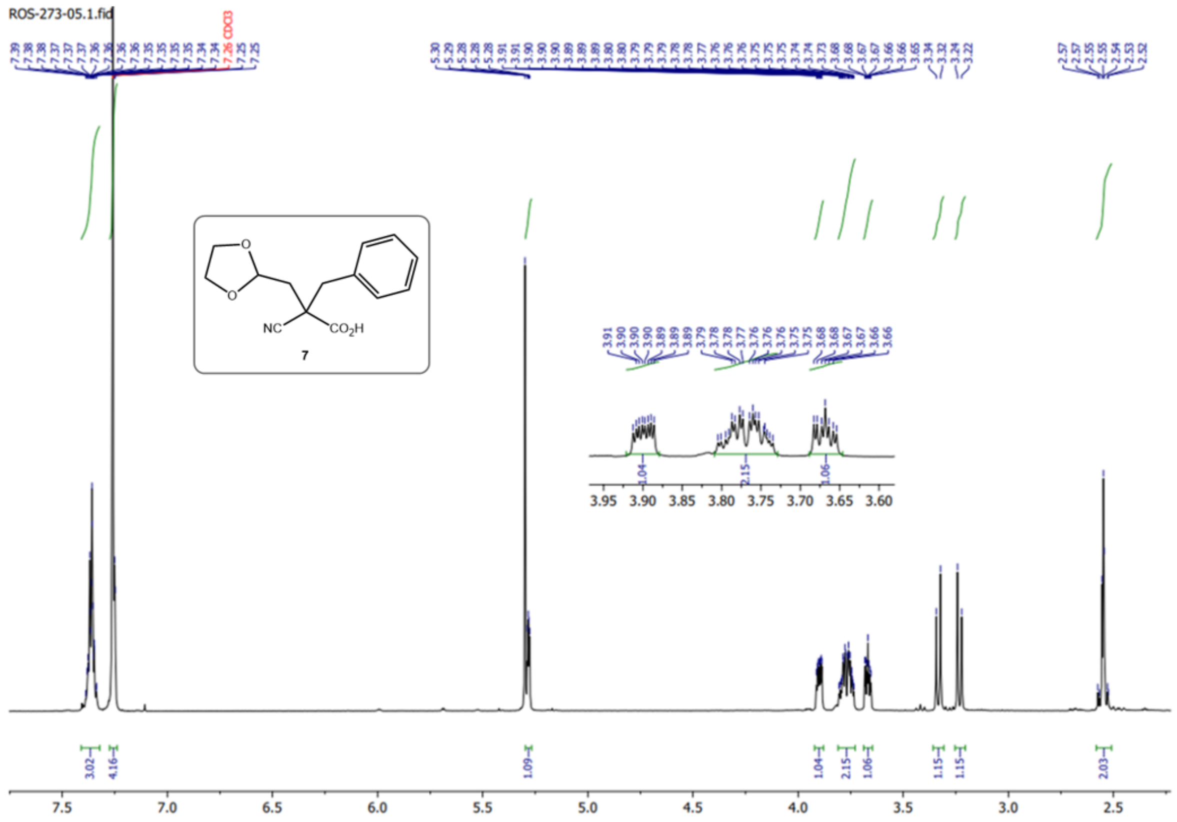

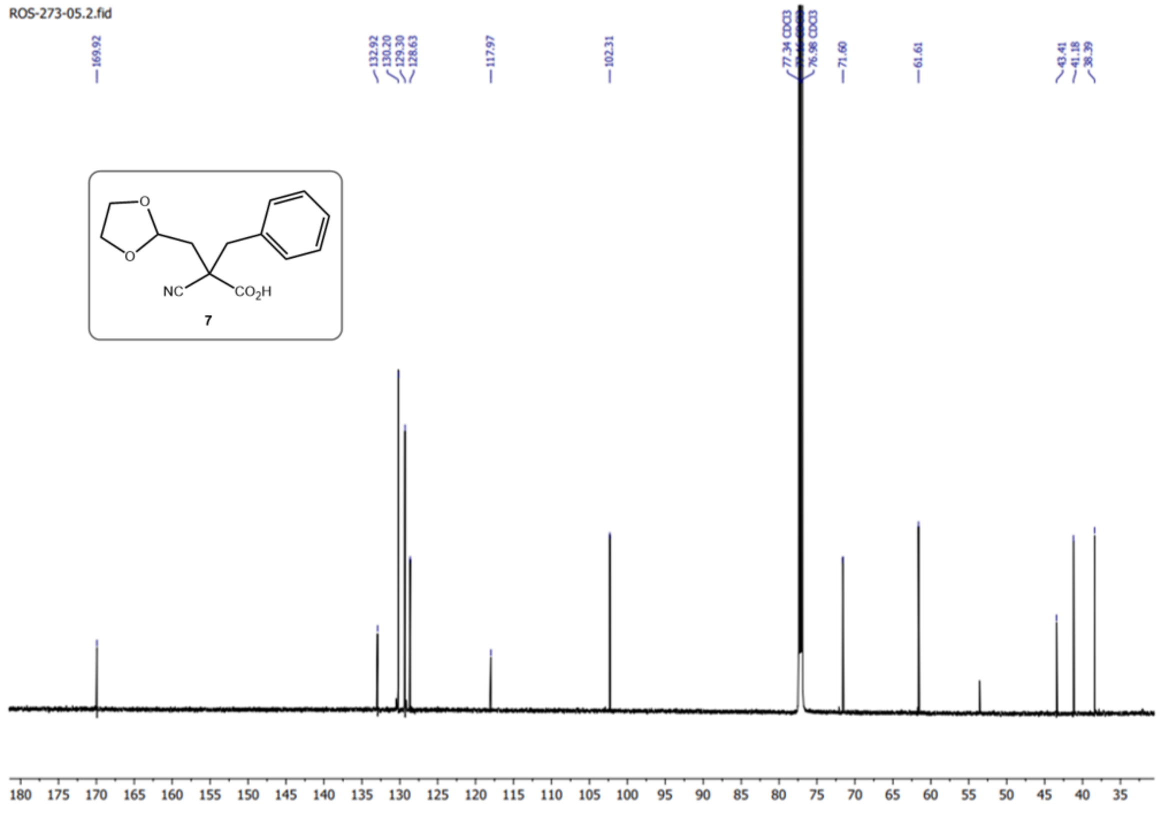

NMR spectra of **8**

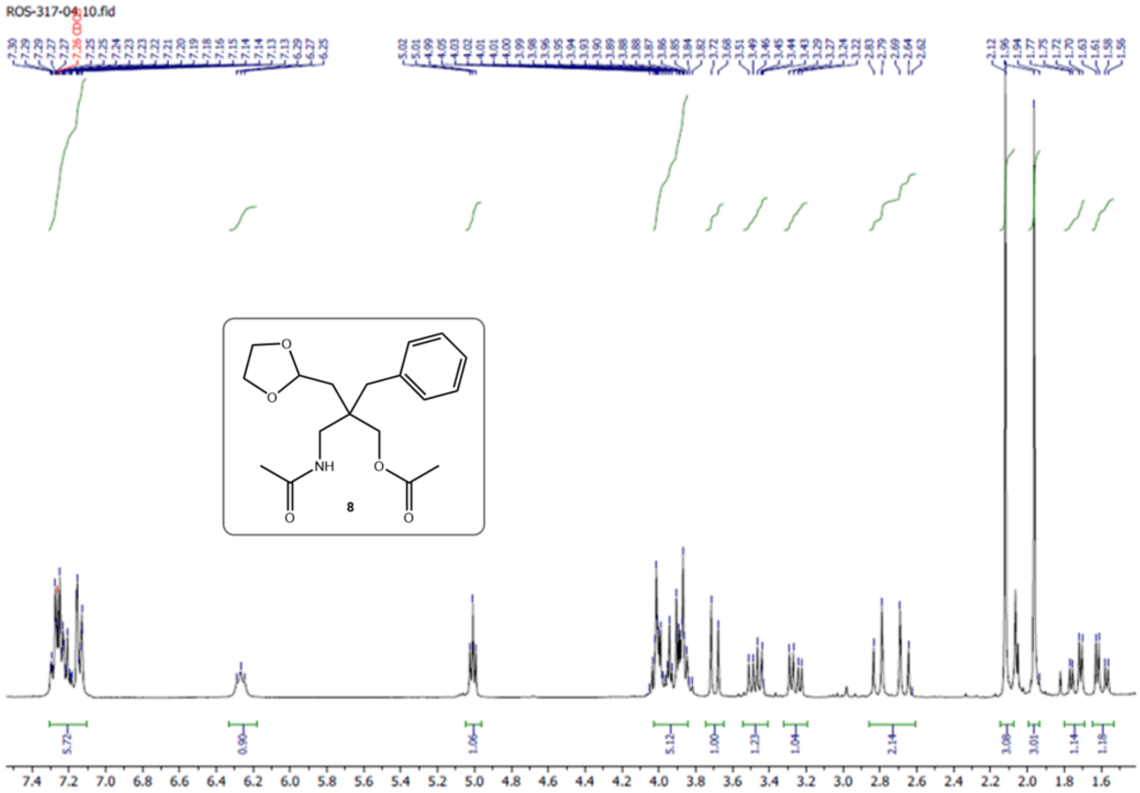

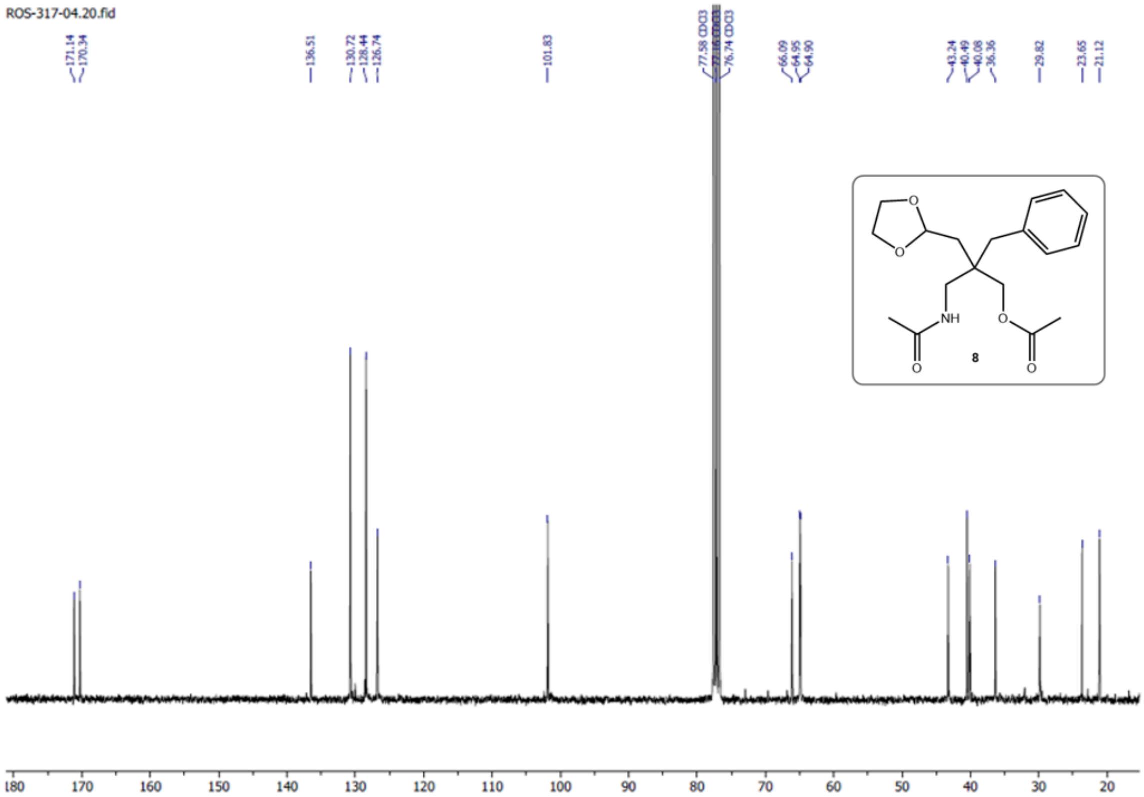

NMR spectra of **9**

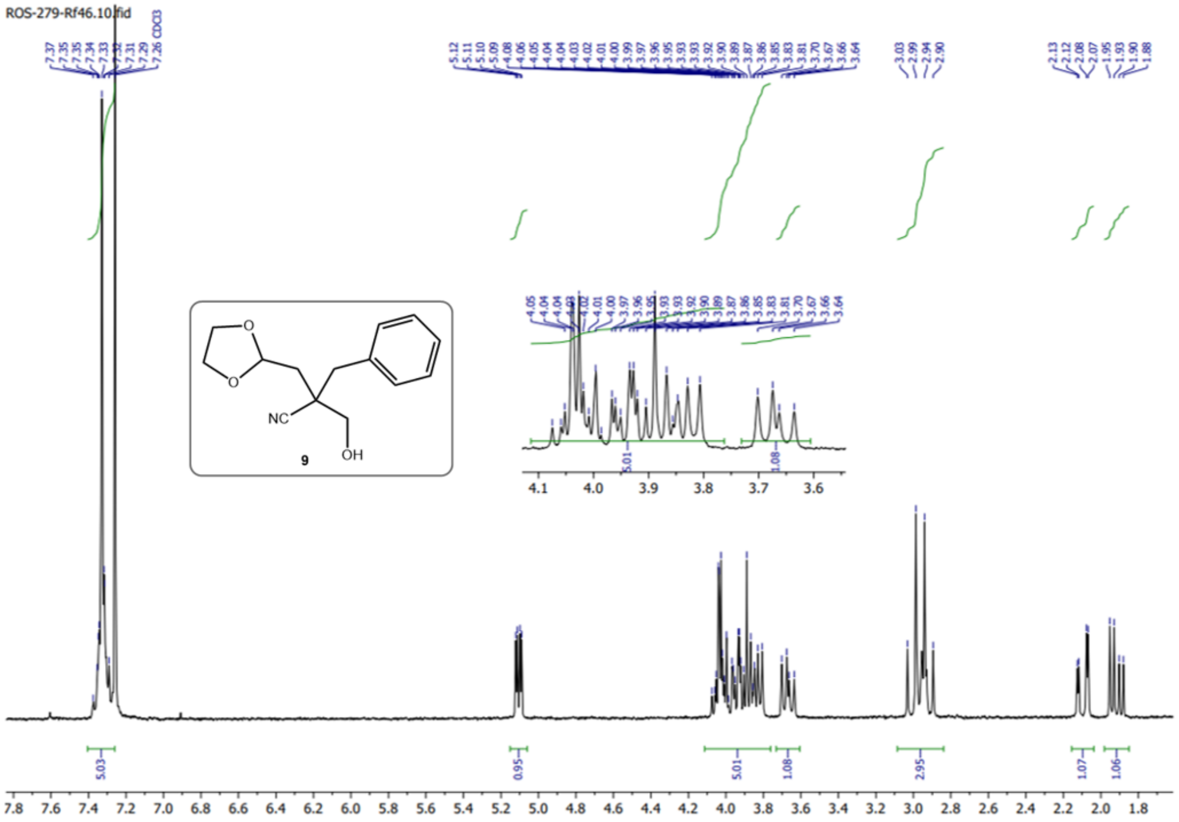

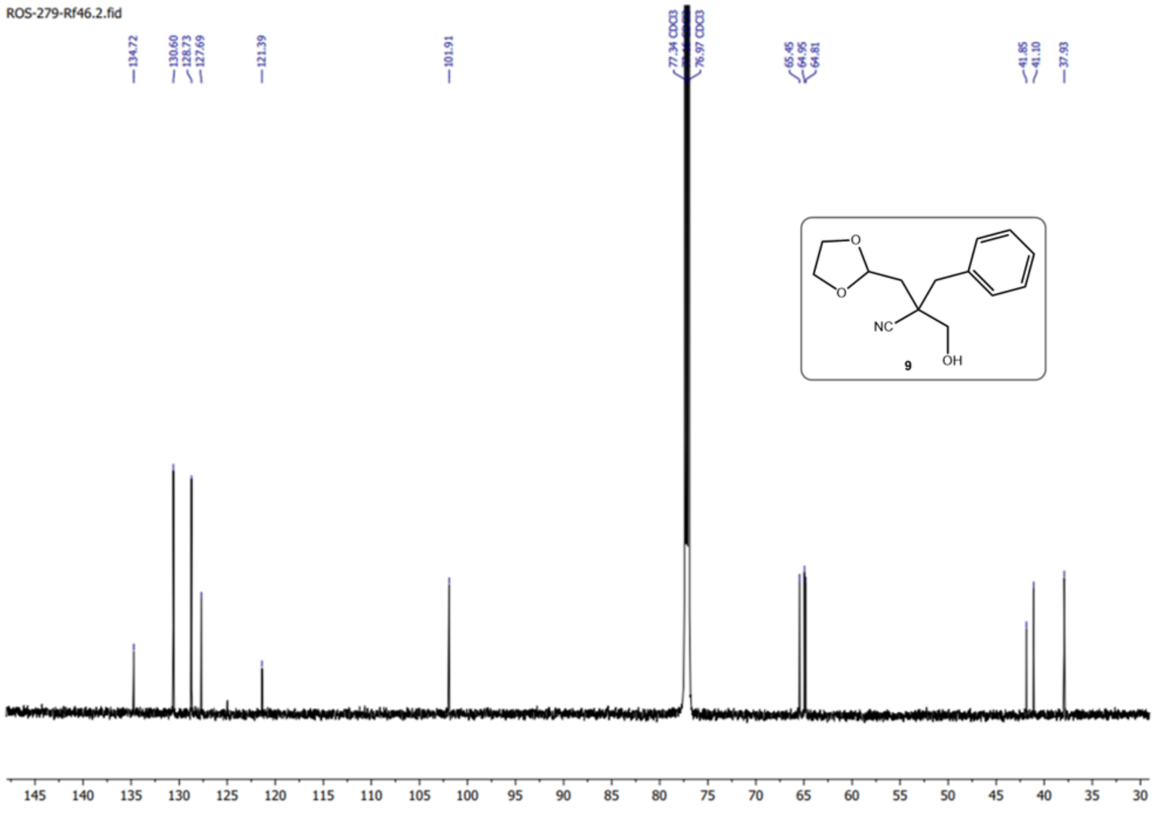

NMR spectra of **10**

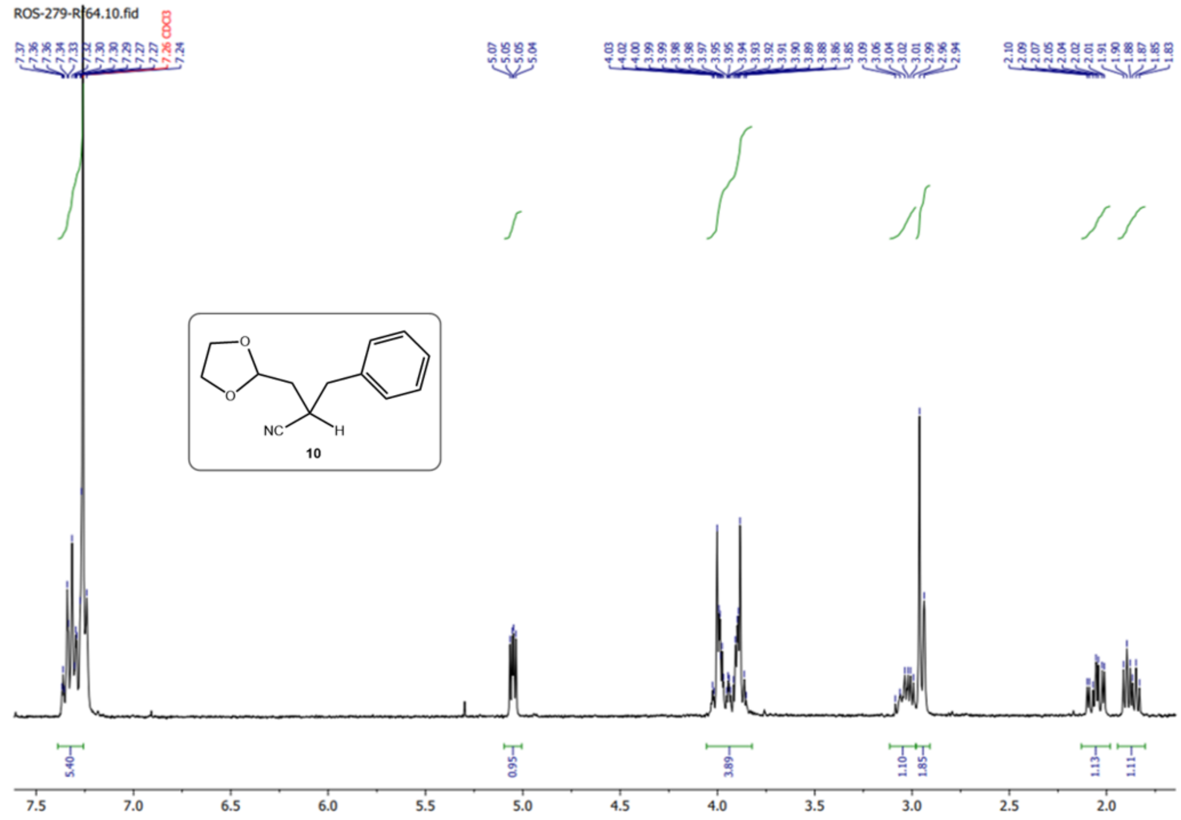

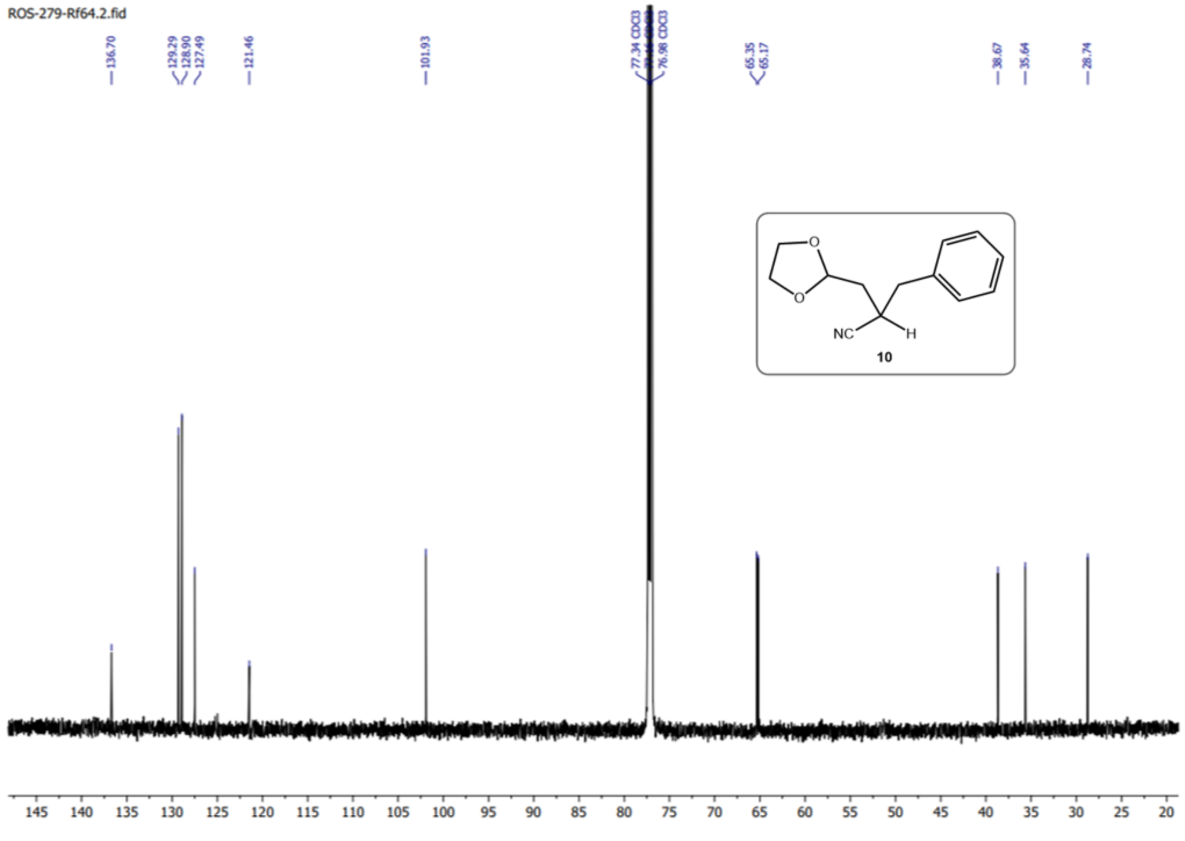

4. HPLC results for the products of the application scope

Chromatogram of racemic product **5a**

Chromatogram

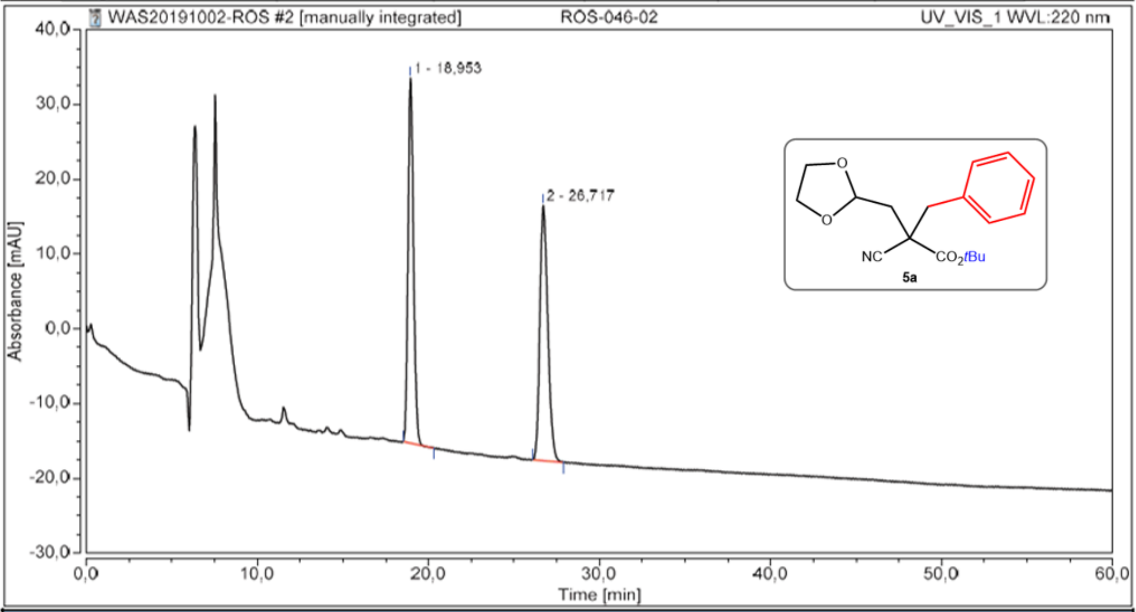

Integration Results

No.	Peak Name	Retention Time min	Area mAU*min	Height mAU	Relative Area %	Relative Height %	Amount n.a.
1		18,953	17,857	48,860	49,40	58,87	n.a.
2		26,717	18,294	34,134	50,60	41,13	n.a.
**Total:**			**36,152**	**82,994**	**100,00**	**100,00**	

Chromatogram of enantioenriched product 5**a**

Chromatogram

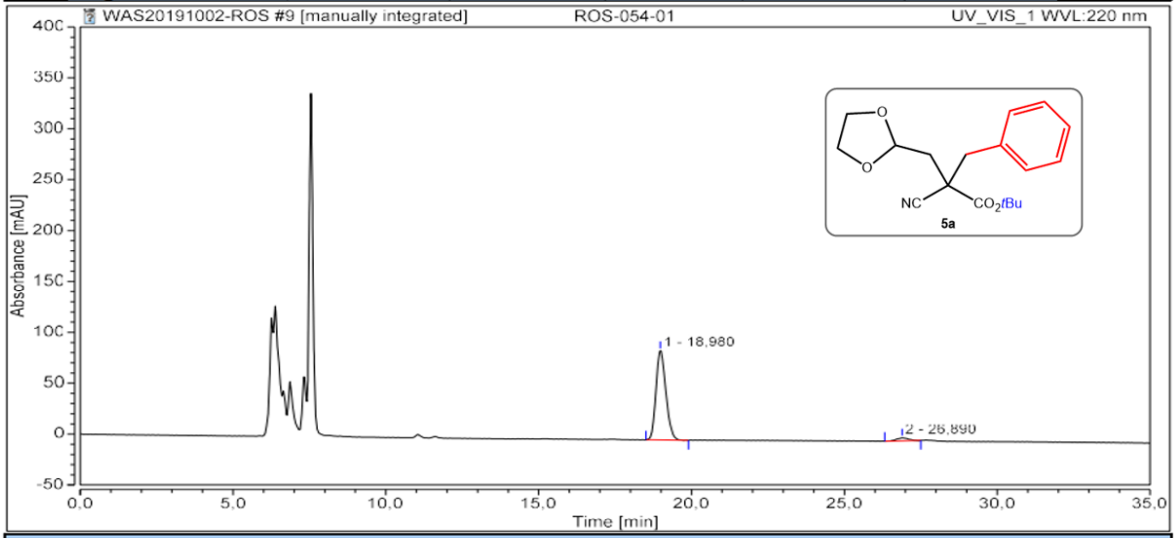

Integration Results

No.	Peak Name	Retention Time min	Area mAU*min	Height mAU	Relative Area %	Relative Height %	Amount n.a.
1		18,980	32,944	87,990	96,31	96,79	n.a.
2		26,890	1,260	2,916	3,69	3,21	n.a.
**Total:**			**34,204**	**90,905**	**100,00**	**100,00**	

Chromatogram of racemic product **5b**

Chromatogram

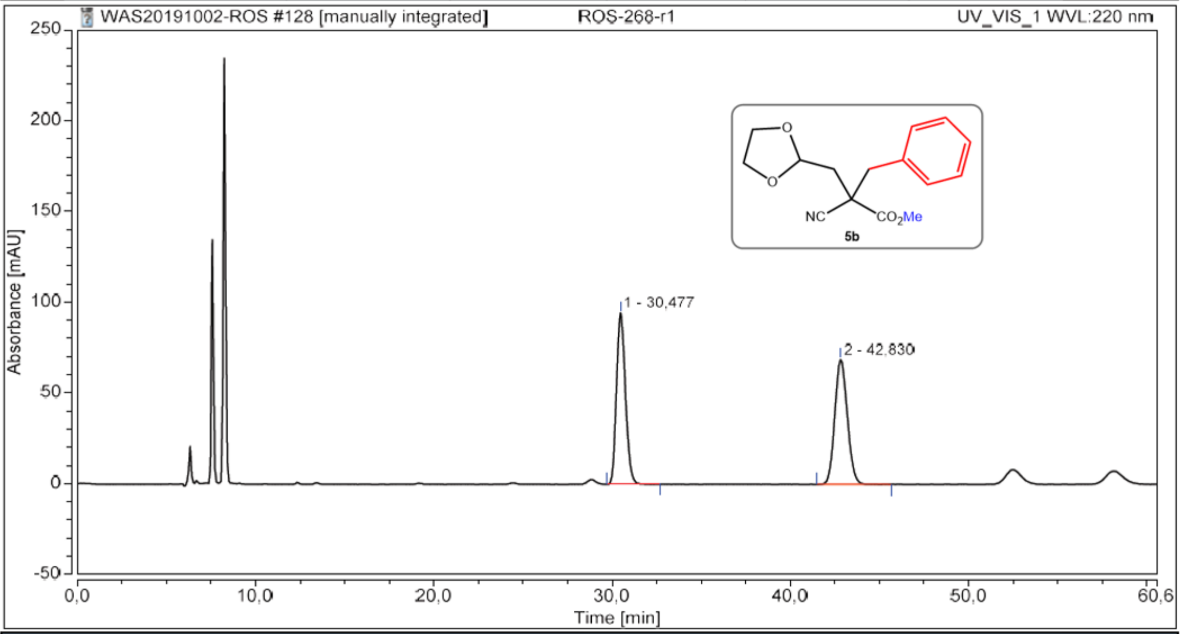

Integration Results

No.	Peak Name	Retention Time min	Area mAU*min	Height mAU	Relative Area %	Relative Height %	Amount n.a.
1		30,477	54,785	94,364	49,49	57,87	n.a.
2		42,830	55,909	68,689	50,51	42,13	n.a.
**Total:**			**110,694**	**163,054**	**100,00**	**100,00**	

Chromatogram of enantioenriched product 5**b**

Chromatogram

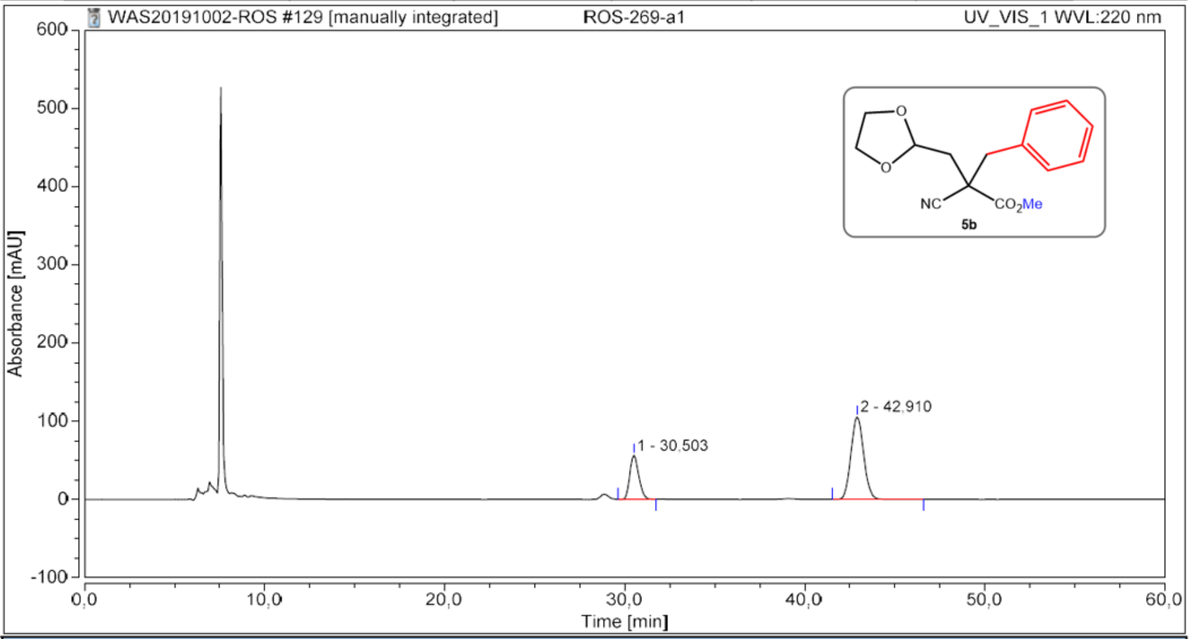

Integration Results

No.	Peak Name	Retention Time min	Area mAU*min	Height mAU	Relative Area %	Relative Height %	Amount n.a.
1		30,503	32,289	56,332	27,06	34,76	n.a.
2		42,910	87,027	105,720	72,94	65,24	n.a.
**Total:**			**119,316**	**162,052**	**100,00**	**100,00**	

Chromatogram of racemic product **5c**

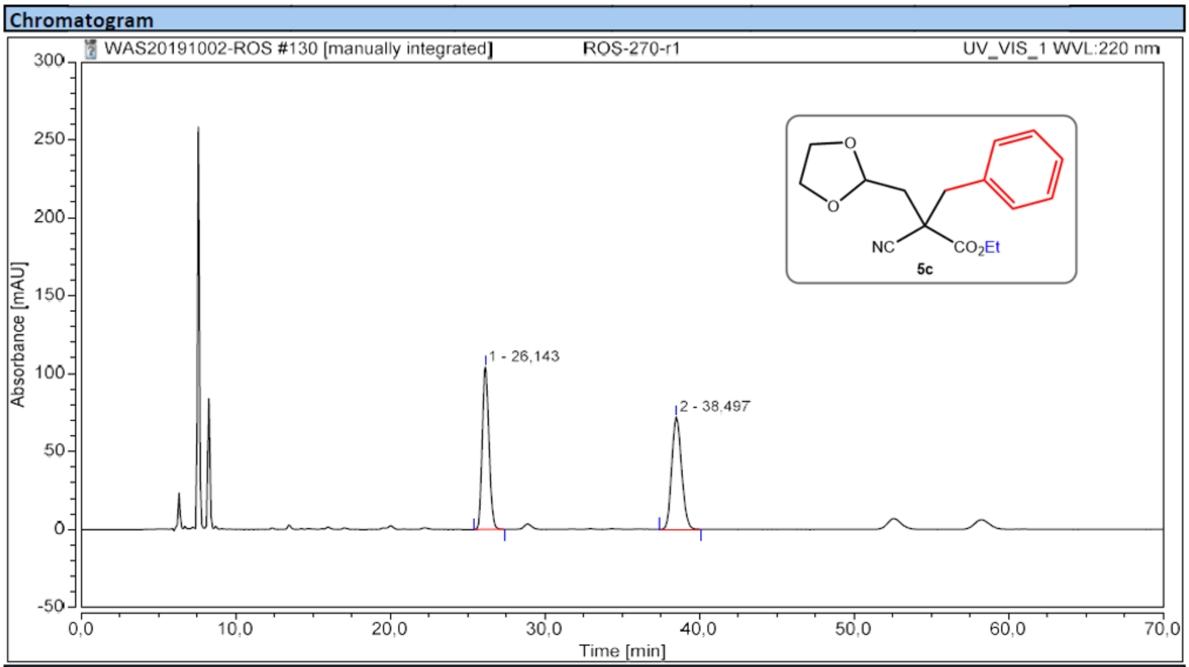

Integration Results

No.	Peak Name	Retention Time min	Area mAU*min	Height mAU	Relative Area %	Relative Height %	Amount n.a.
1		26,143	52,133	104,364	49,58	59,16	n.a.
2		38,497	53,019	72,047	50,42	40,84	n.a.
**Total:**			**105,152**	**176,411**	**100,00**	**100,00**	

Chromatogram of enantioenriched product 5**c**

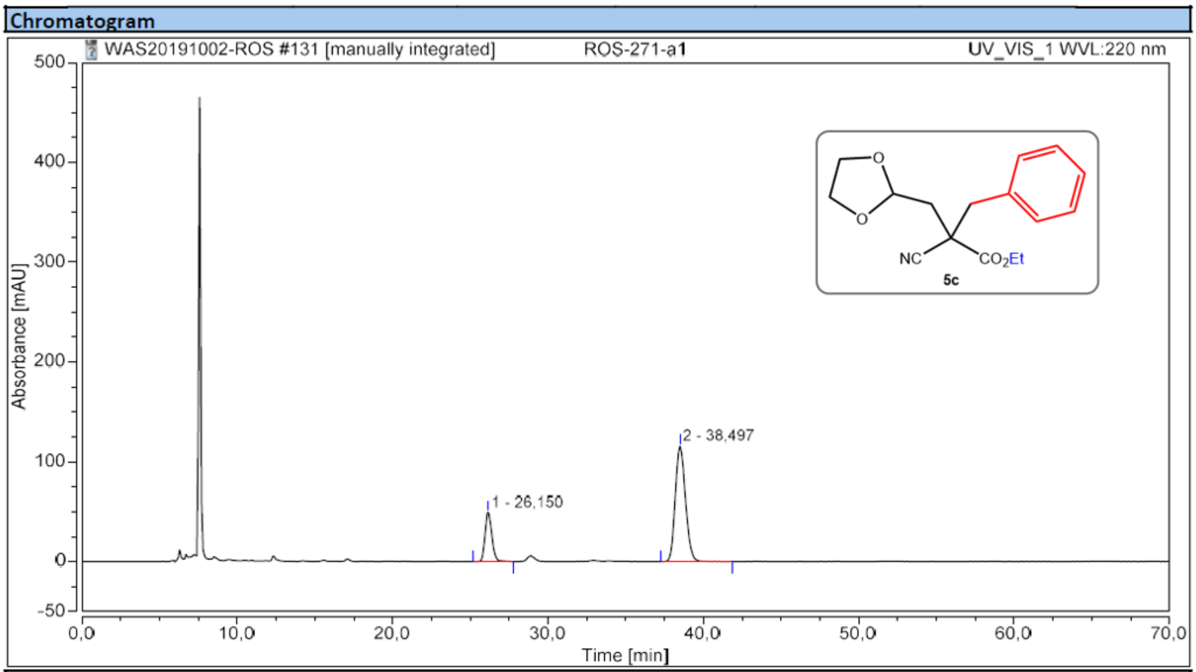

Integration Results

No.	Peak Name	Retention Time min	Area mAU*min	Height mAU	Relative Area %	Relative Height %	Amount n.a.
1		26,150	24,752	49,599	22,07	29,99	n.a.
2		38,497	87,392	115,814	77,93	70,01	n.a.
**Total:**			**112,144**	**165,413**	**100,00**	**100,00**	

Chromatogram of racemic product **5d**

Chromatogram

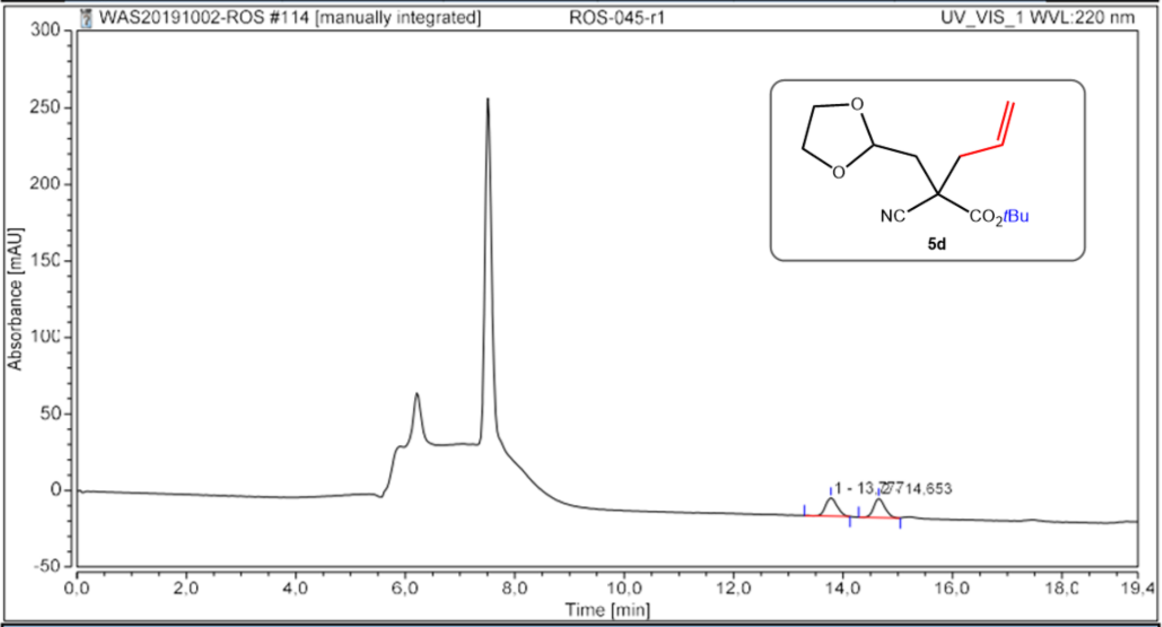

Integration Results

No.	Peak Name	Retention Time min	Area mAU*min	Height mAU	Relative Area %	Relative Height %	Amount n.a.
1		13,777	2,930	11,863	50,40	49,10	n.a.
2		14,653	2,884	12,299	49,60	50,90	n.a.
**Total:**			**5,813**	**24,162**	**100,00**	**100,00**	

Chromatogram of enantioenriched product **5d**

Chromatogram

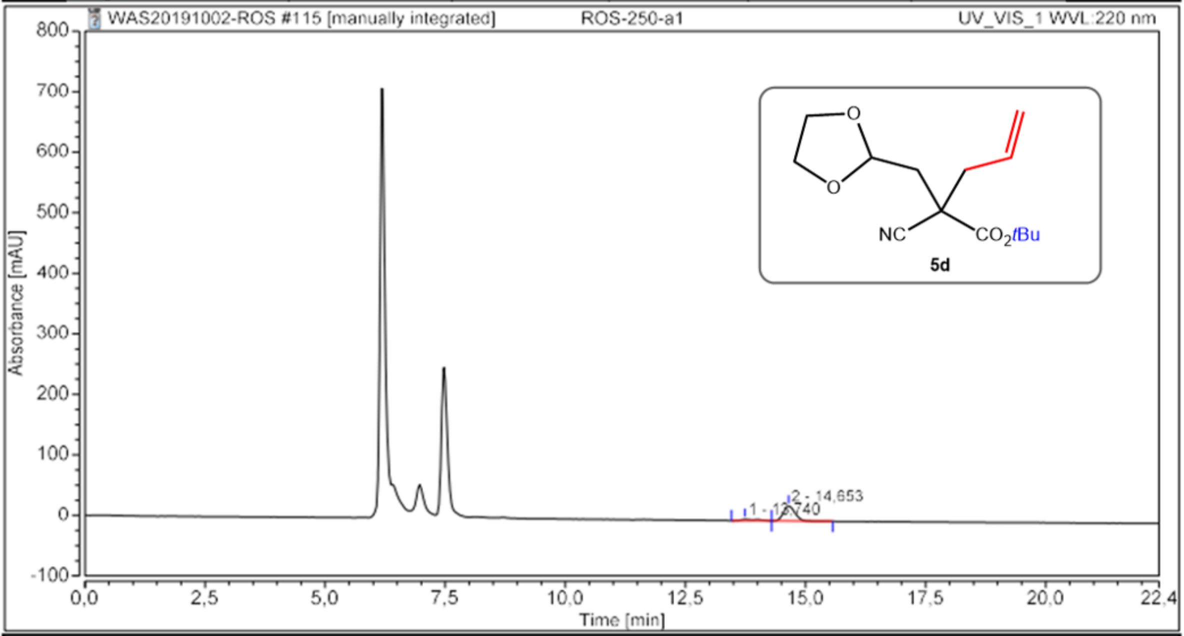

Integration Results

No.	Peak Name	Retention Time min	Area mAU*min	Height mAU	Relative Area %	Relative Height %	Amount n.a.
1		13,740	0,878	2,048	11,48	7,74	n.a.
2		14,653	6,771	24,408	88,52	92,26	n.a.
**Total:**			**7,649**	**26,455**	**100,00**	**100,00**	

Chromatogram of racemic product **5e**

Chromatogram

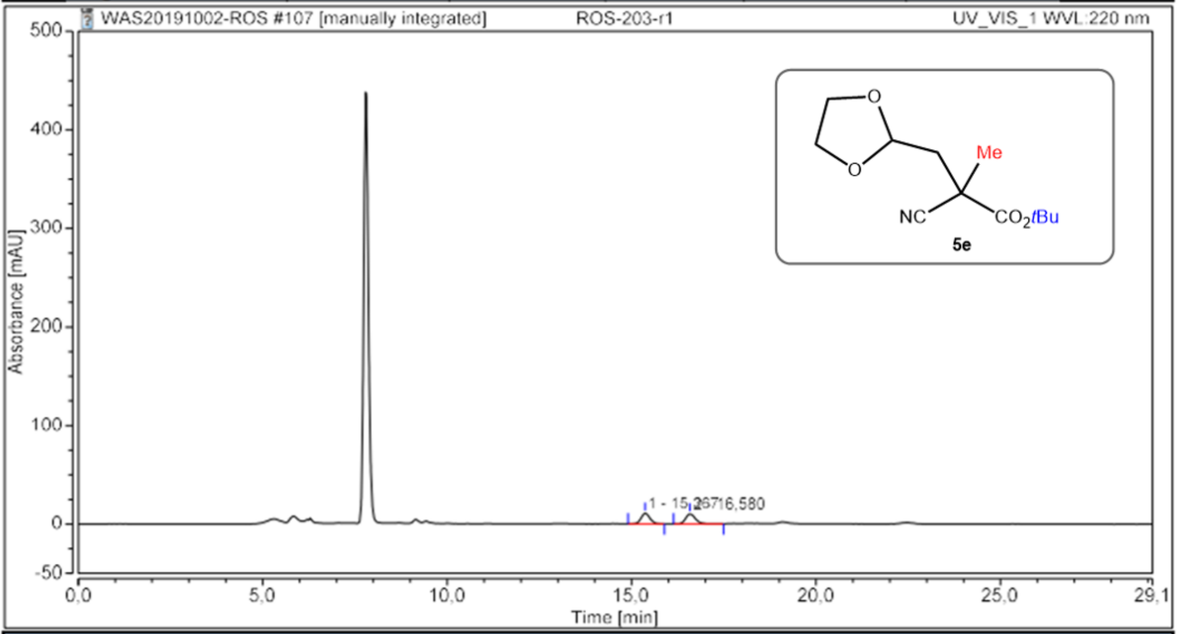

Integration Results

No.	Peak Name	Retention Time min	Area mAU*min	Height mAU	Relative Area %	Relative Height %	Amount n.a.
1		15,367	3,003	10,796	49,72	51,81	n.a.
2		16,580	3,037	10,043	50,28	48,19	n.a.
**Total:**			**6,040**	**20,838**	**100,00**	**100,00**	

Chromatogram of enantioenriched product **5e**

Chromatogram

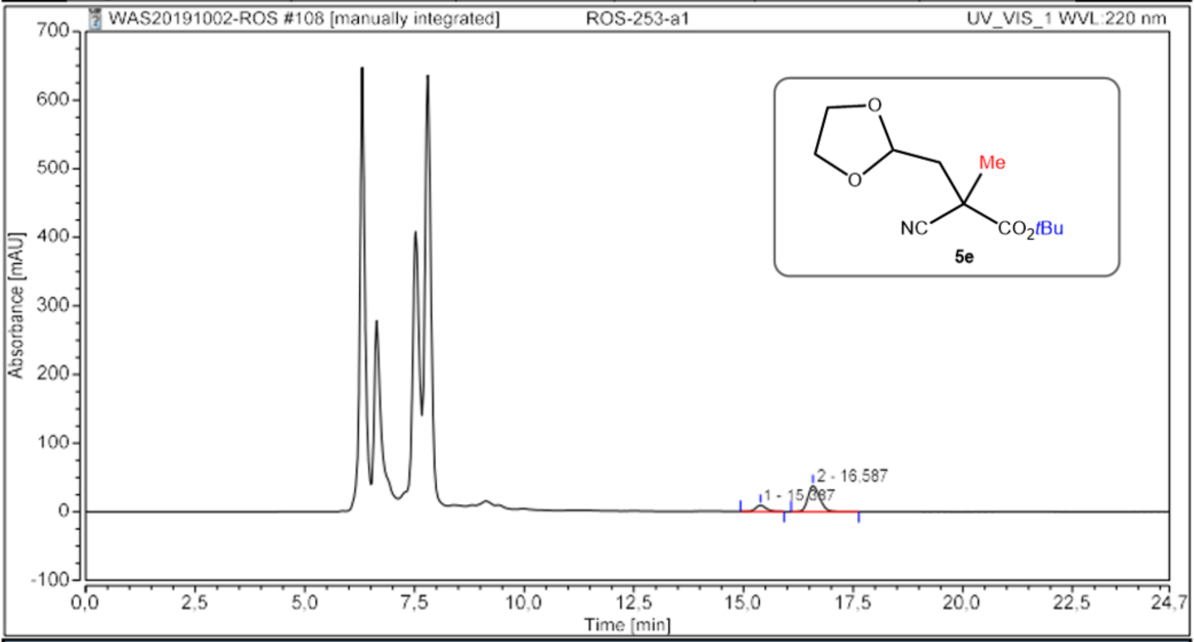

Integration Results

No.	Peak Name	Retention Time min	Area mAU*min	Height mAU	Relative Area %	Relative Height %	Amount n.a.
1		15,387	2,455	8,889	17,56	19,10	n.a.
2		16,587	11,524	37,643	82,44	80,90	n.a.
**Total:**			**13,979**	**46,531**	**100,00**	**100,00**	

Chromatogram of racemic product **5f**

Chromatogram

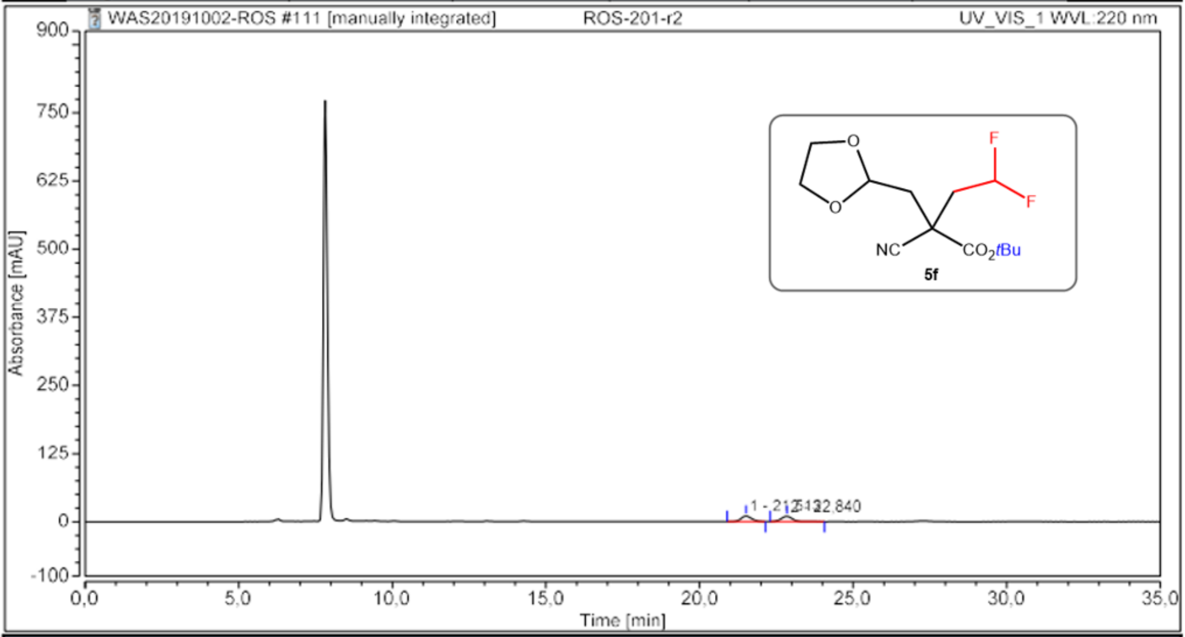

Integration Results

No.	Peak Name	Retention Time min	Area mAU*min	Height mAU	Relative Area %	Relative Height %	Amount n.a.
1		21,513	4,029	10,140	50,33	51,11	n.a.
2		22,840	3,976	9,701	49,67	48,89	n.a.
**Total:**			**8,004**	**19,842**	**100,00**	**100,00**	

Chromatogram of enantioenriched product **5f**

Chromatogram

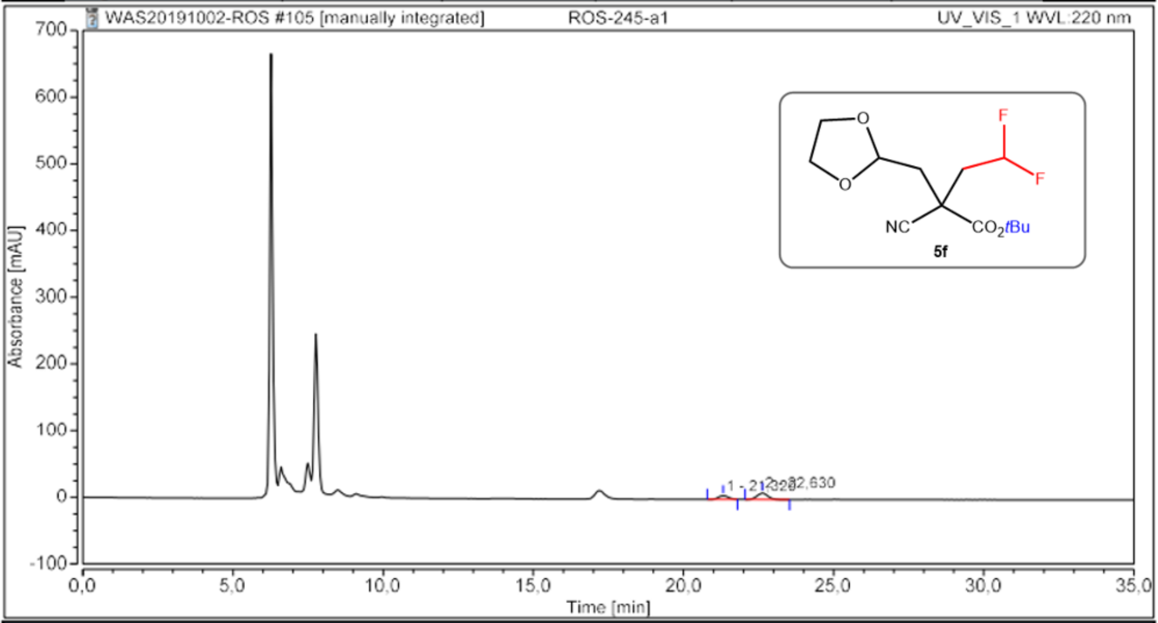

Integration Results

No.	Peak Name	Retention Time min	Area mAU*min	Height mAU	Relative Area %	Relative Height %	Amount n.a.
1		21,320	2,095	5,363	34,82	36,13	n.a.
2		22,630	3,921	9,478	65,18	63,87	n.a.
**Total:**			**6,016**	**14,841**	**100,00**	**100,00**	

Chromatogram of racemic product **5g**

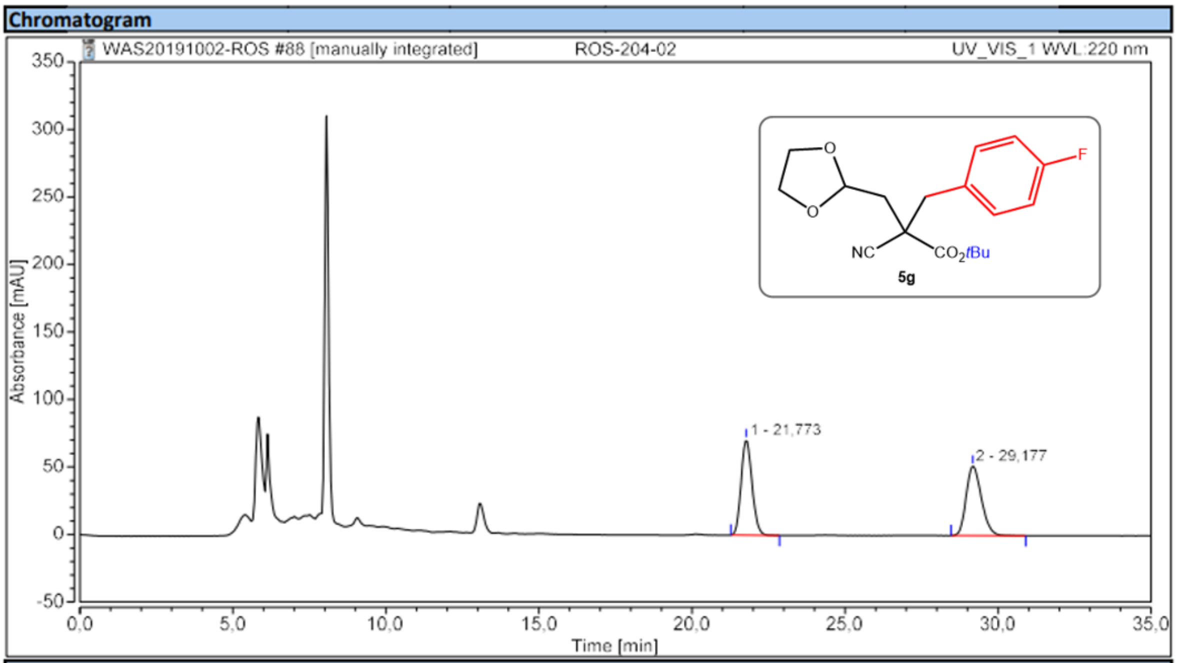

Integration Results							
No.	Peak Name	Retention Time min	Area mAU*min	Height mAU	Relative Area %	Relative Height %	Amount n.a.
1		21,773	29,156	70,580	49,51	57,80	n.a.
2		29,177	29,729	51,535	50,49	42,20	n.a.
**Total:**			**58,885**	**122,115**	**100,00**	**100,00**	

Chromatogram of enantioenriched product **5g**

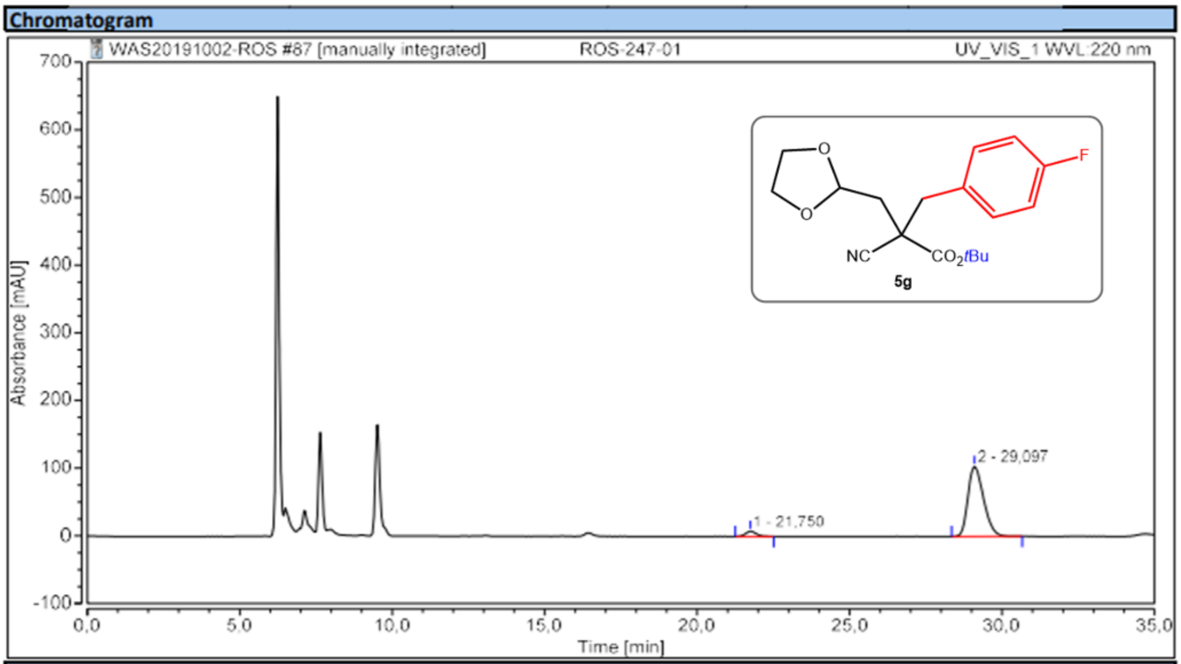

Integration Results							
No.	Peak Name	Retention Time min	Area mAU*min	Height mAU	Relative Area %	Relative Height %	Amount n.a.
1		21,750	3,063	7,588	4,77	6,85	n.a.
2		29,097	61,181	103,232	95,23	93,15	n.a.
**Total:**			**64,244**	**110,820**	**100,00**	**100,00**	

Chromatogram of racemic product **5h**

Chromatogram

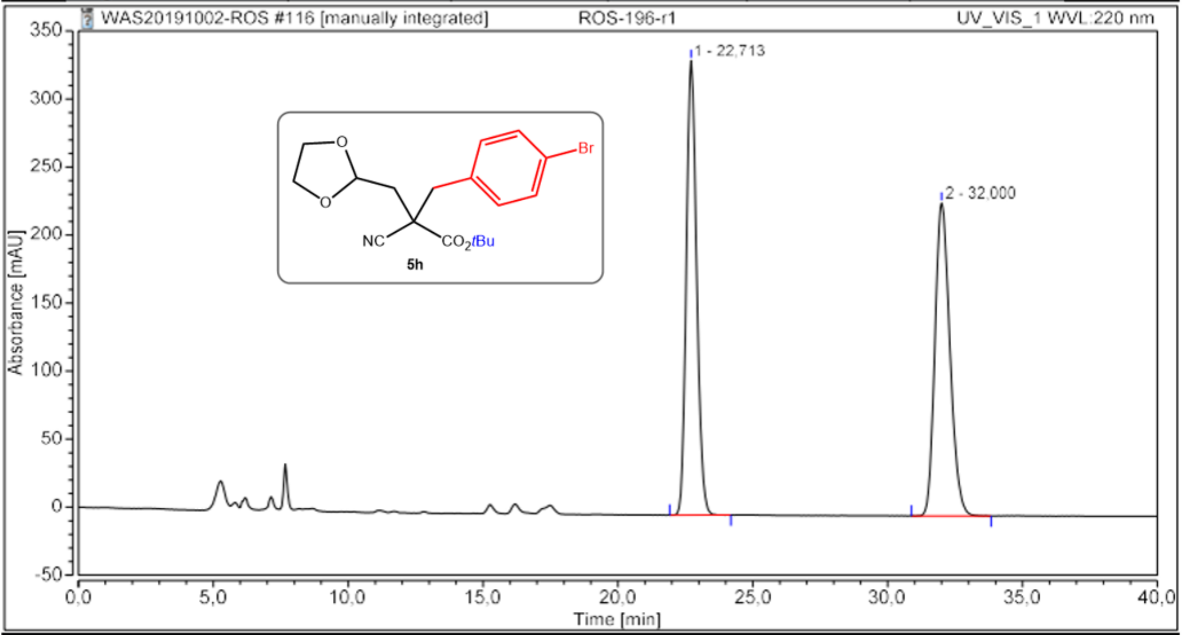

Integration Results

No.	Peak Name	Retention Time min	Area mAU*min	Height mAU	Relative Area %	Relative Height %	Amount n.a.
1		22,713	147,884	334,134	49,94	59,23	n.a.
2		32,000	148,243	229,972	50,06	40,77	n.a.
**Total:**			**296,127**	**564,106**	**100,00**	**100,00**	

Chromatogram of enantioenriched product **5h**

Chromatogram

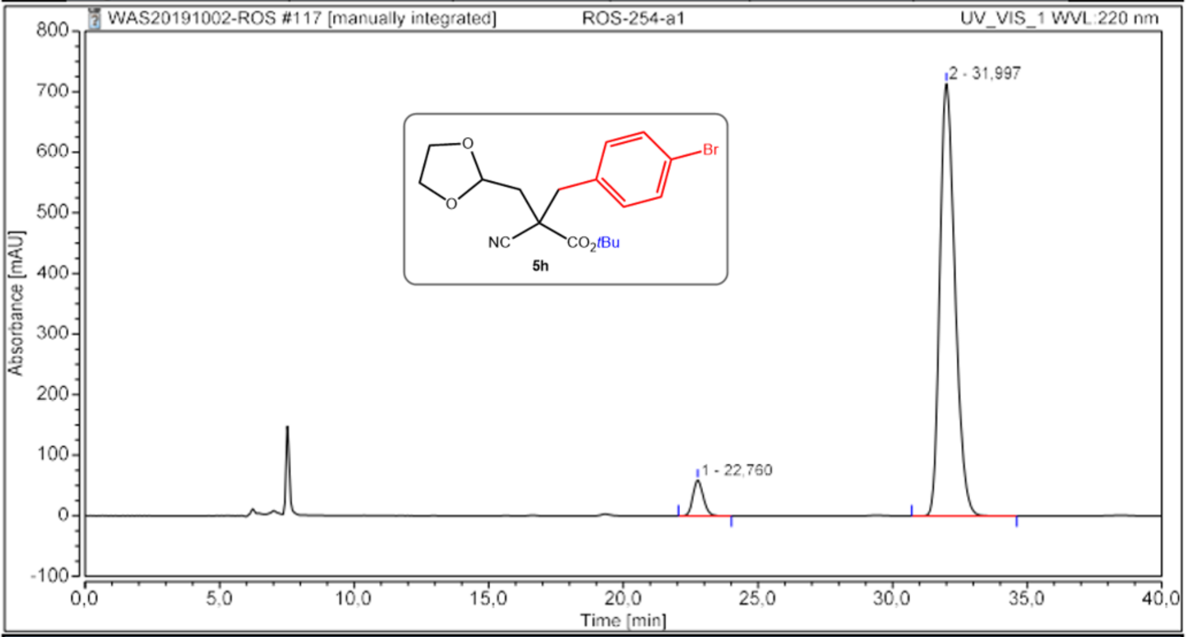

Integration Results

No.	Peak Name	Retention Time min	Area mAU*min	Height mAU	Relative Area %	Relative Height %	Amount n.a.
1		22,760	26,226	59,183	5,32	7,65	n.a.
2		31,997	467,121	714,053	94,68	92,35	n.a.
**Total:**			**493,346**	**773,236**	**100,00**	**100,00**	

Chromatogram of racemic product **5i**

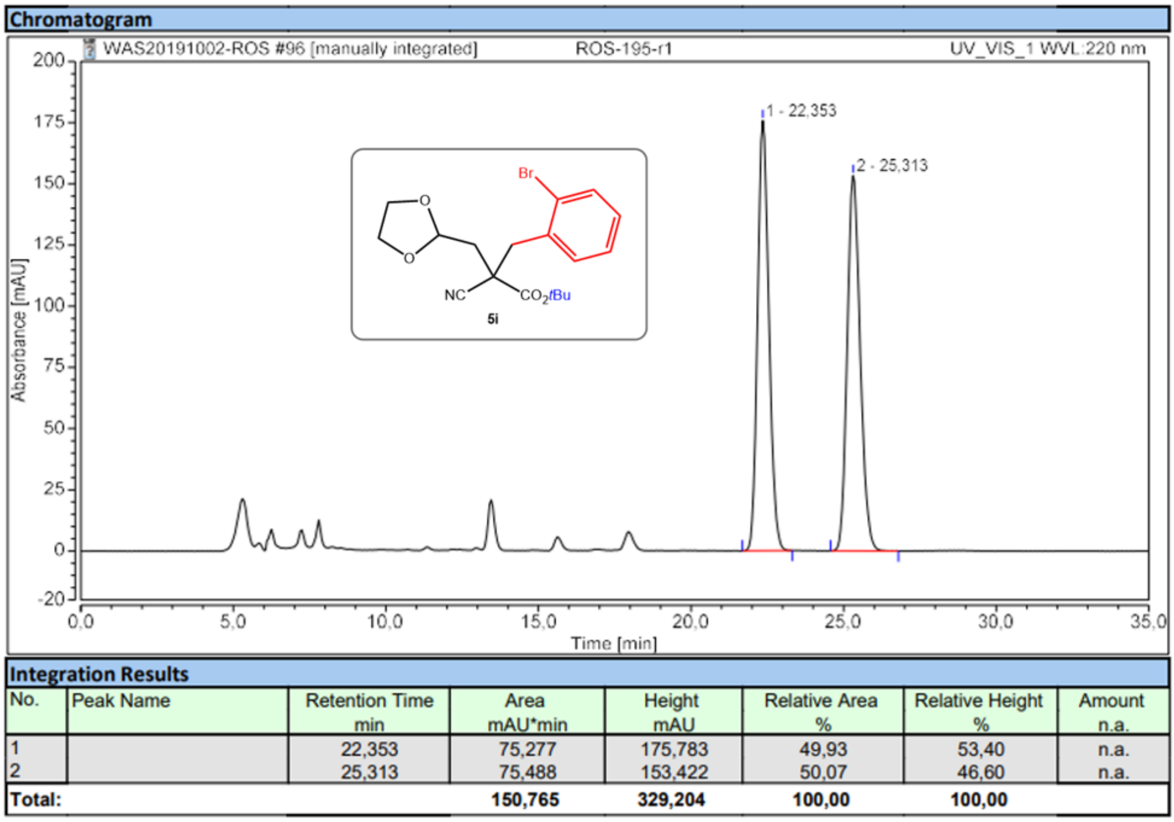

Integration Results

No.	Peak Name	Retention Time min	Area mAU*min	Height mAU	Relative Area %	Relative Height %	Amount n.a.
1		22,353	75,277	175,783	49,93	53,40	n.a.
2		25,313	75,488	153,422	50,07	46,60	n.a.
**Total:**			**150,765**	**329,204**	**100,00**	**100,00**	

Chromatogram of enantioenriched product **5i**

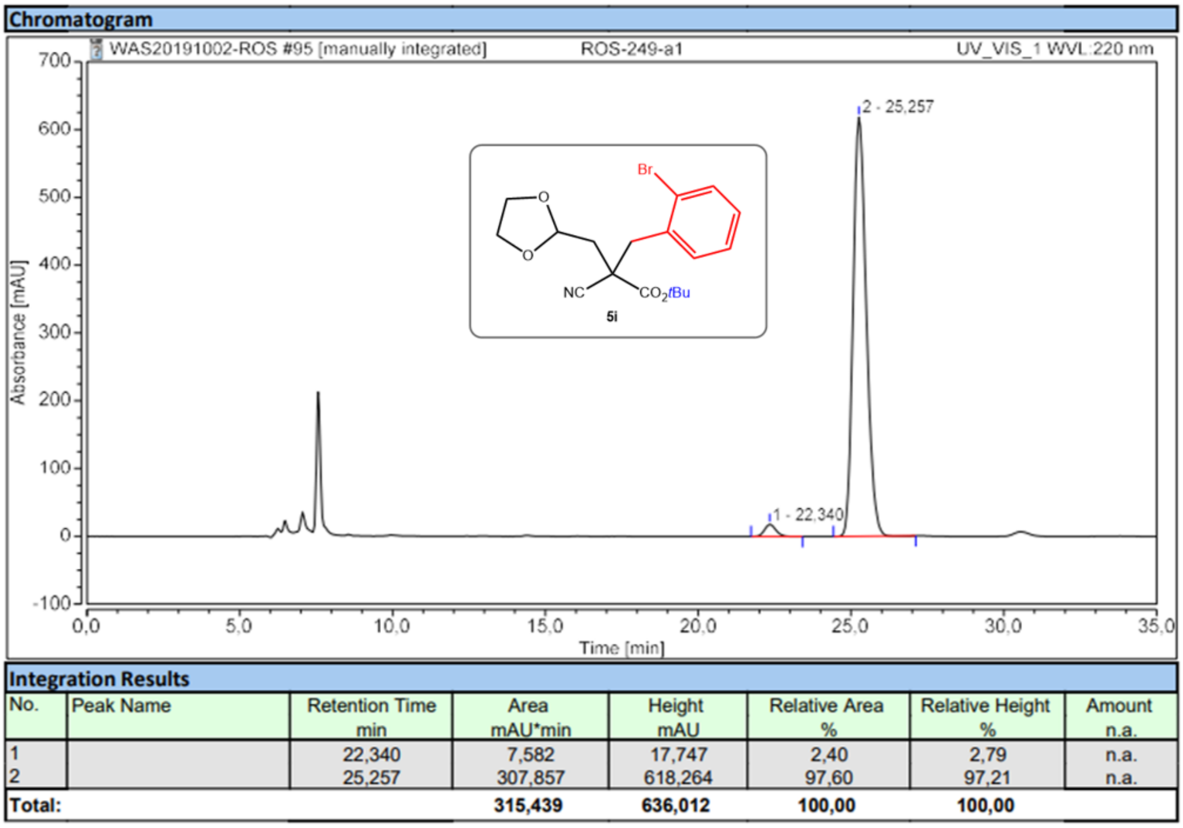

Integration Results

No.	Peak Name	Retention Time min	Area mAU*min	Height mAU	Relative Area %	Relative Height %	Amount n.a.
1		22,340	7,582	17,747	2,40	2,79	n.a.
2		25,257	307,857	618,264	97,60	97,21	n.a.
**Total:**			**315,439**	**636,012**	**100,00**	**100,00**	

Chromatogram of racemic product **5j**

Chromatogram

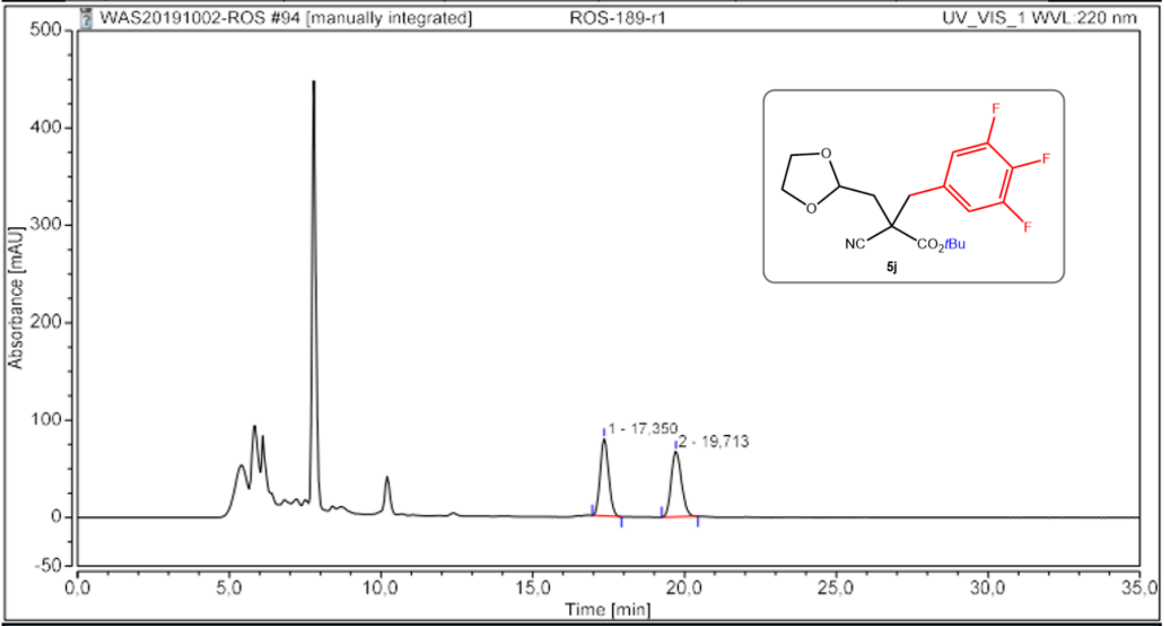

Integration Results

No.	Peak Name	Retention Time min	Area mAU*min	Height mAU	Relative Area %	Relative Height %	Amount n.a.
1		17,350	25,690	79,202	49,44	54,06	n.a.
2		19,713	26,272	67,305	50,56	45,94	n.a.
**Total:**			**51,962**	**146,508**	**100,00**	**100,00**	

Chromatogram of enantioenriched product **5j**

Chromatogram

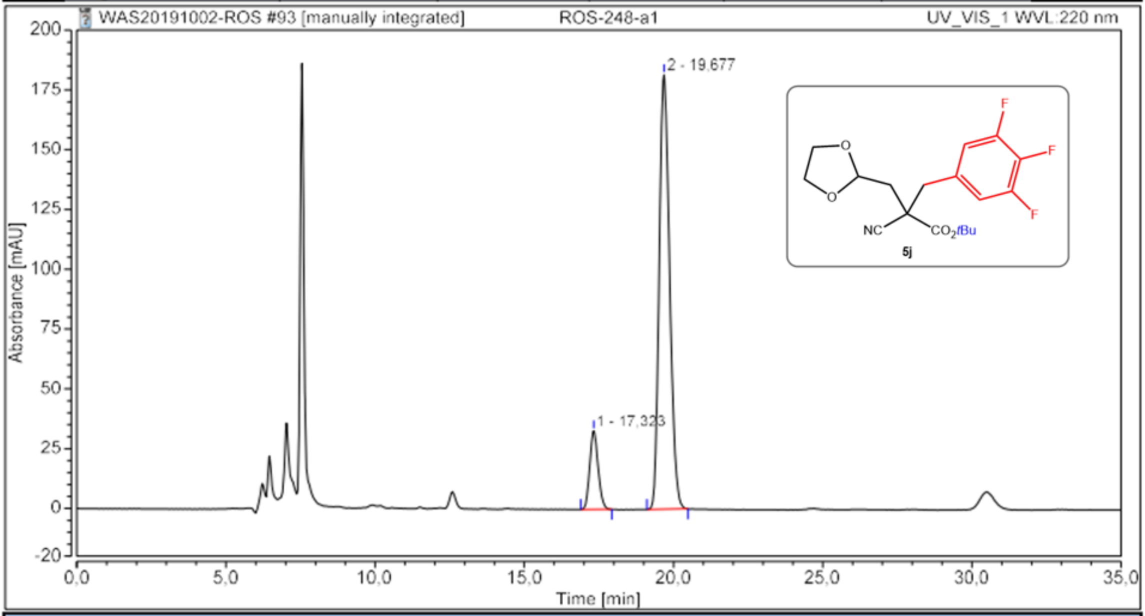

Integration Results

No.	Peak Name	Retention Time min	Area mAU*min	Height mAU	Relative Area %	Relative Height %	Amount n.a.
1		17,323	10,704	32,843	12,77	15,30	n.a.
2		19,677	73,138	181,752	87,23	84,70	n.a.
**Total:**			**83,842**	**214,595**	**100,00**	**100,00**	

Chromatogram of racemic product **5k**

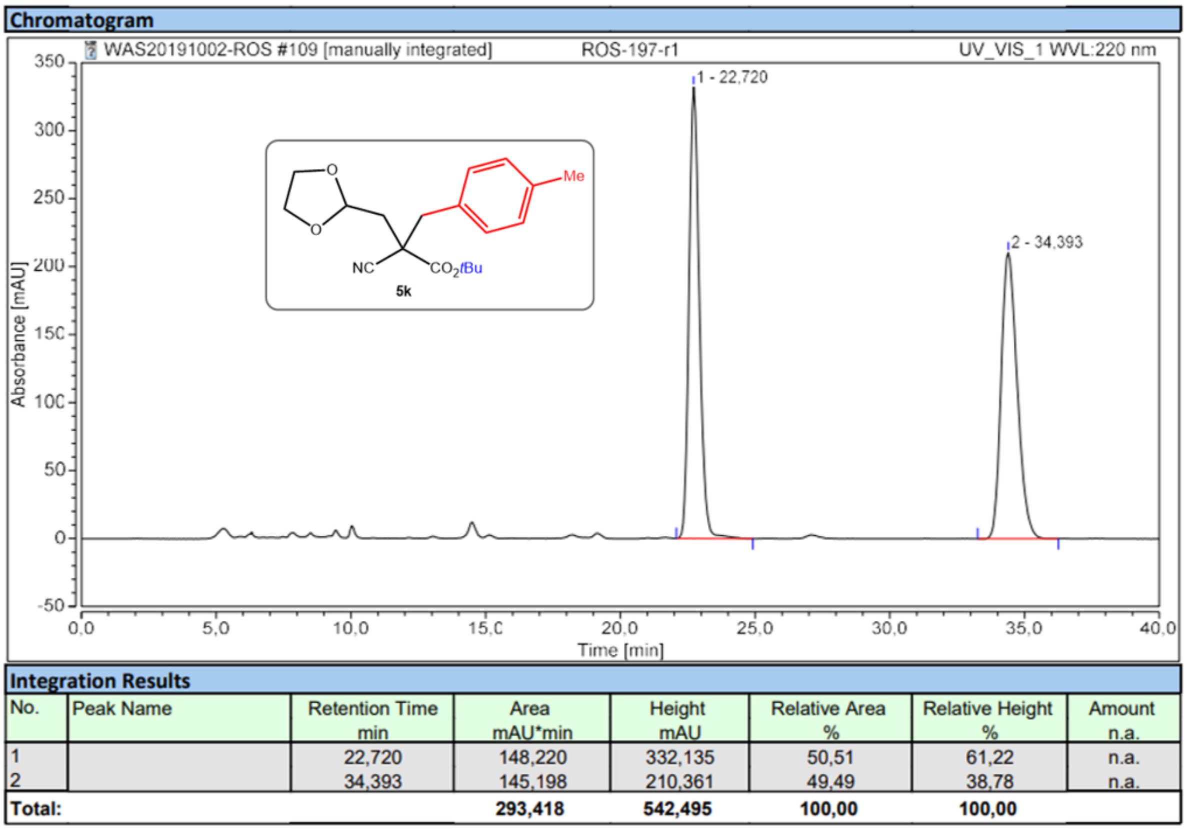

Integration Results

No.	Peak Name	Retention Time min	Area mAU*min	Height mAU	Relative Area %	Relative Height %	Amount n.a.
1		22,720	148,220	332,135	50,51	61,22	n.a.
2		34,393	145,198	210,361	49,49	38,78	n.a.
**Total:**			**293,418**	**542,495**	**100,00**	**100,00**	

Chromatogram of enantioenriched product **5k**

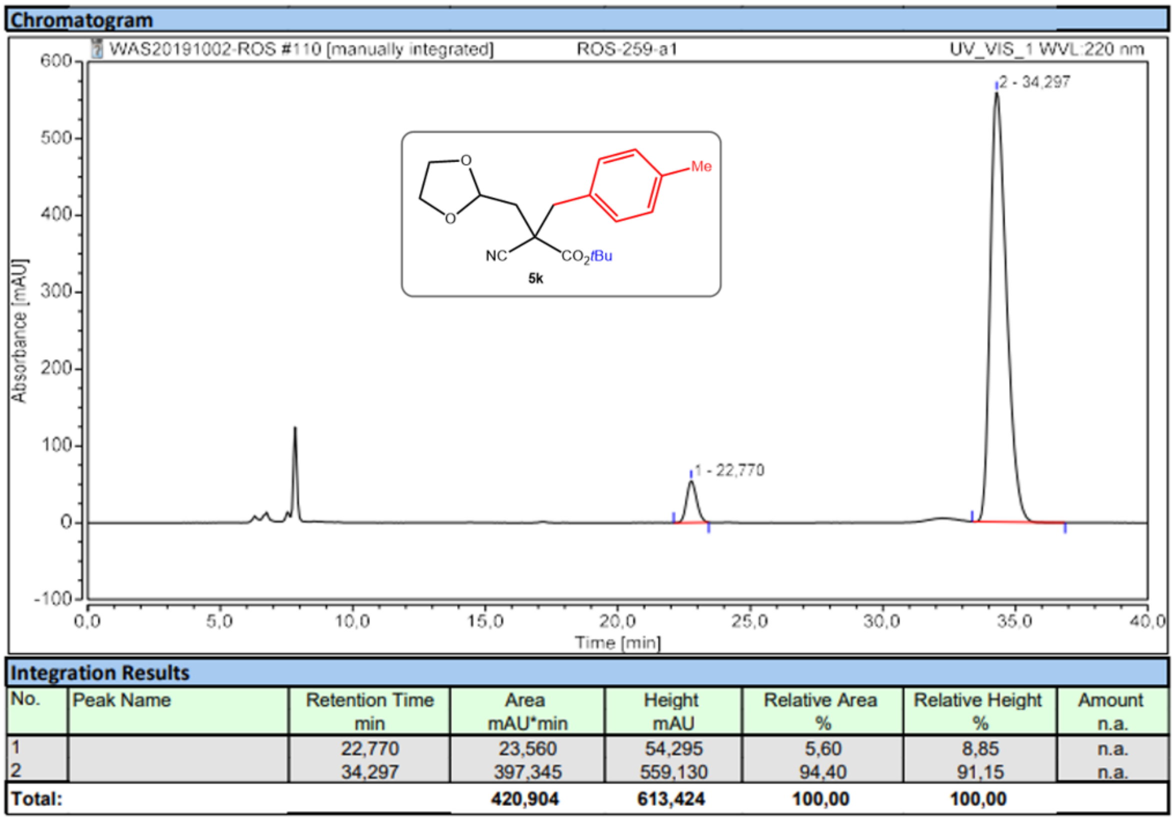

Integration Results

No.	Peak Name	Retention Time min	Area mAU*min	Height mAU	Relative Area %	Relative Height %	Amount n.a.
1		22,770	23,560	54,295	5,60	8,85	n.a.
2		34,297	397,345	559,130	94,40	91,15	n.a.
**Total:**			**420,904**	**613,424**	**100,00**	**100,00**	

Chromatogram of racemic product **5l**

Chromatogram

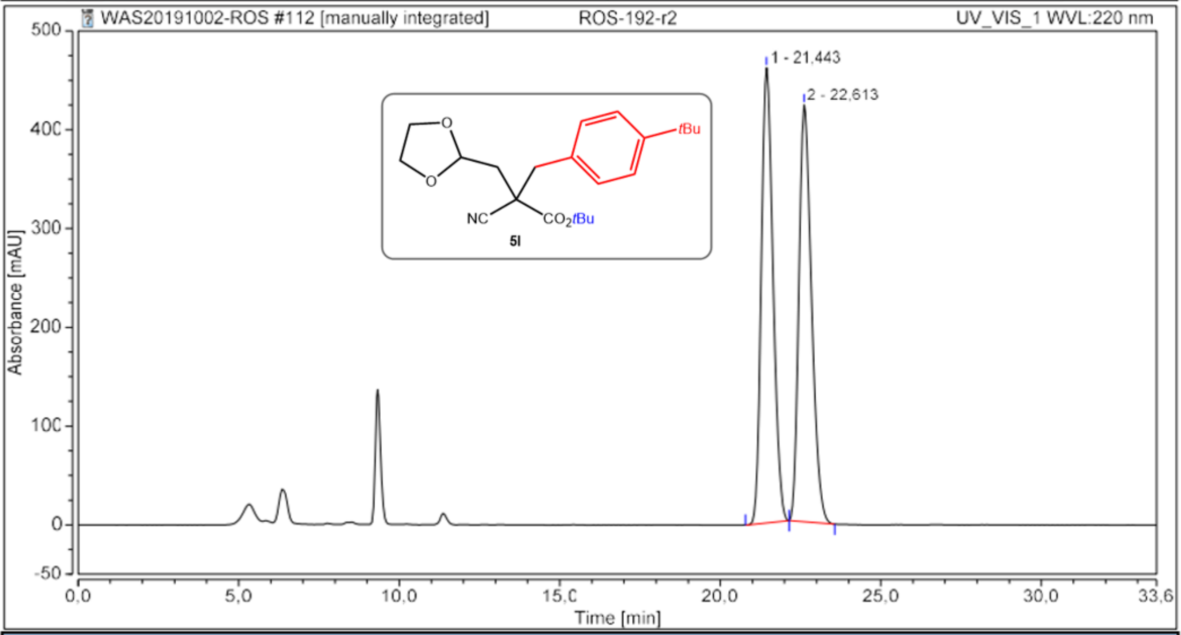

Integration Results

No.	Peak Name	Retention Time min	Area mAU*min	Height mAU	Relative Area %	Relative Height %	Amount n.a.
1		21,443	193,720	460,712	50,02	52,20	n.a.
2		22,613	193,598	421,951	49,98	47,80	n.a.
**Total:**			**387,318**	**882,663**	**100,00**	**100,00**	

Chromatogram of enantioenriched product **5l**

Chromatogram

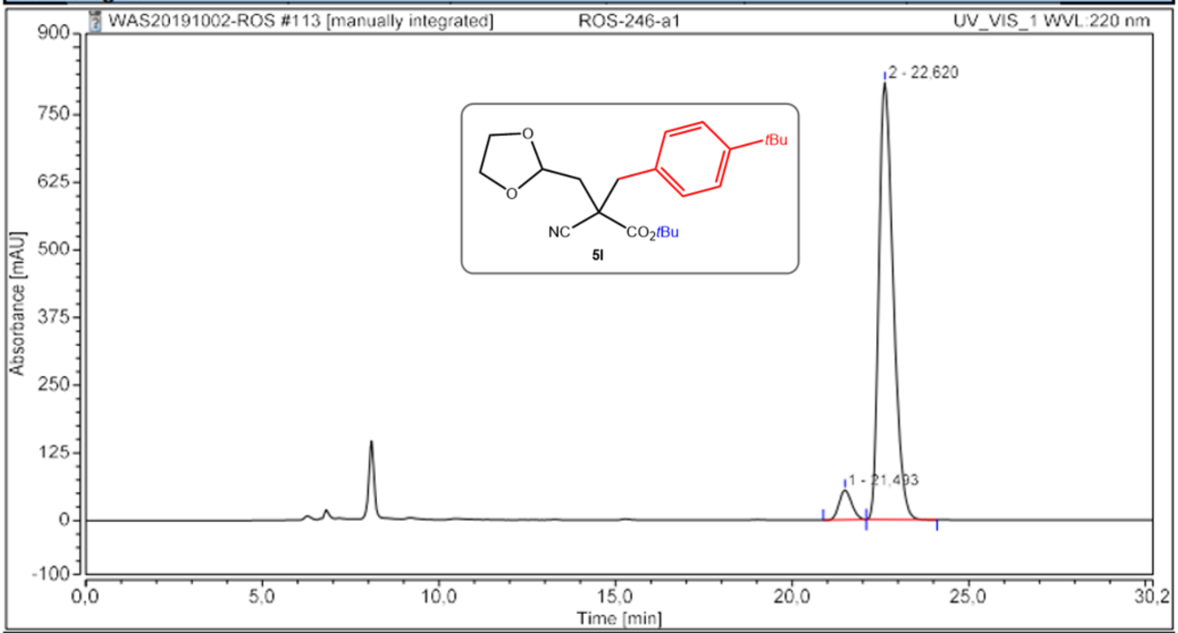

Integration Results

No.	Peak Name	Retention Time min	Area mAU*min	Height mAU	Relative Area %	Relative Height %	Amount n.a.
1		21,493	22,530	54,721	5,59	6,34	n.a.
2		22,620	380,825	808,314	94,41	93,66	n.a.
**Total:**			**403,355**	**863,035**	**100,00**	**100,00**	

Chromatogram of racemic product **5m**

Chromatogram

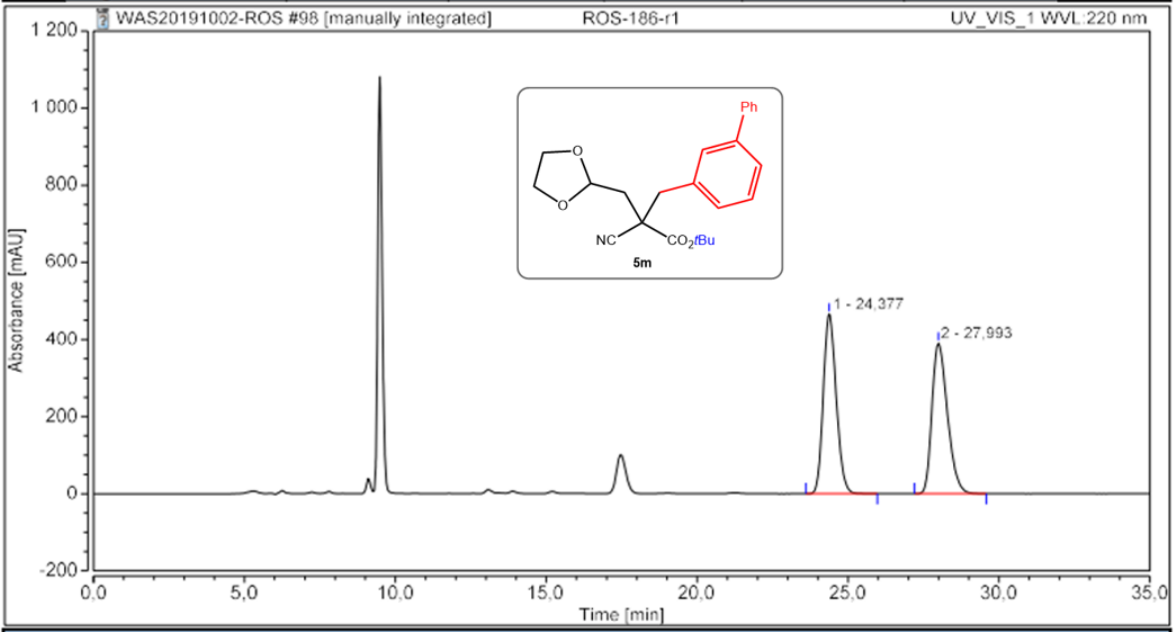

Integration Results

No.	Peak Name	Retention Time min	Area mAU*min	Height mAU	Relative Area %	Relative Height %	Amount n.a.
1		24,377	230,280	465,531	49,84	54,41	n.a.
2		27,993	231,745	390,096	50,16	45,59	n.a.
**Total:**			**462,025**	**855,627**	**100,00**	**100,00**	

Chromatogram of enantioenriched product **5m**

Chromatogram

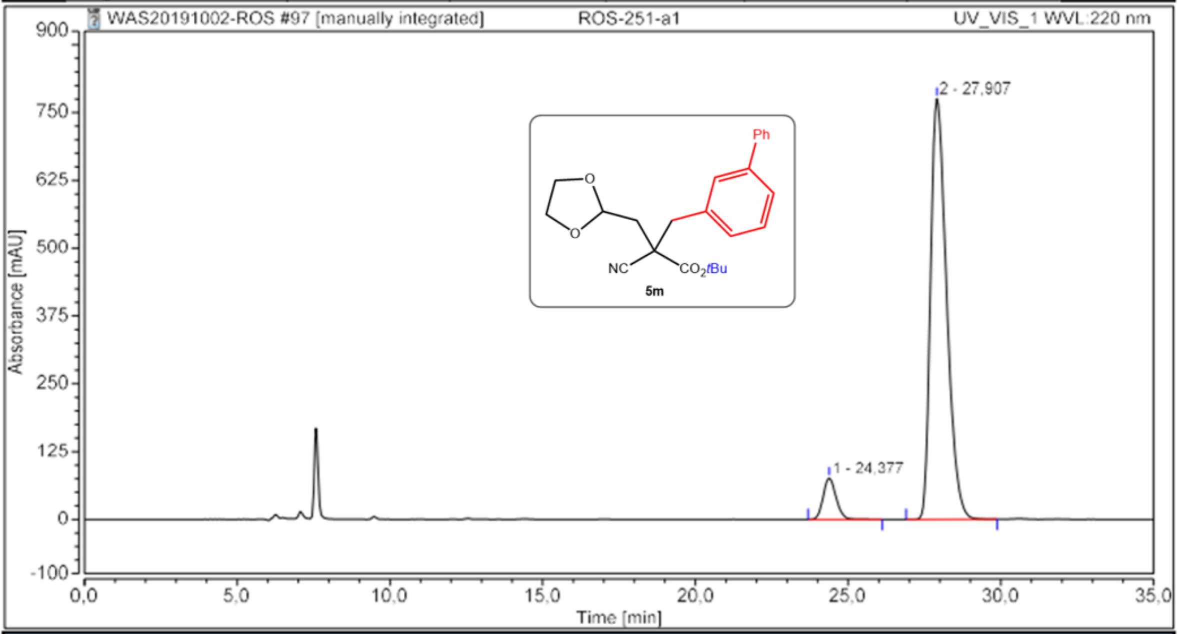

Integration Results

No.	Peak Name	Retention Time min	Area mAU*min	Height mAU	Relative Area %	Relative Height %	Amount n.a.
1		24,377	37,229	76,371	7,29	8,96	n.a.
2		27,907	473,653	776,370	92,71	91,04	n.a.
**Total:**			**510,882**	**852,742**	**100,00**	**100,00**	

Chromatogram of racemic product **5n**

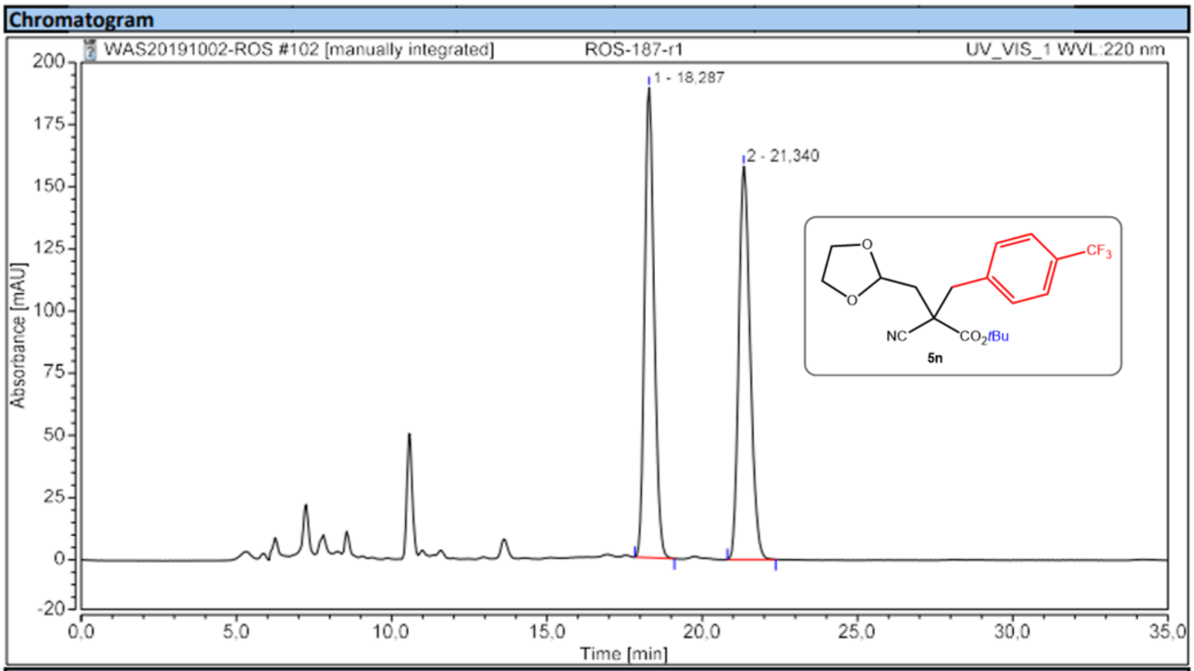

Integration Results

No.	Peak Name	Retention Time min	Area mAU*min	Height mAU	Relative Area %	Relative Height %	Amount n.a.
1		18,287	67,058	189,054	49,86	54,44	n.a.
2		21,340	67,435	158,195	50,14	45,56	n.a.
Total:			134,493	347,249	100,00	100,00	

Chromatogram of enantioenriched product **5n**

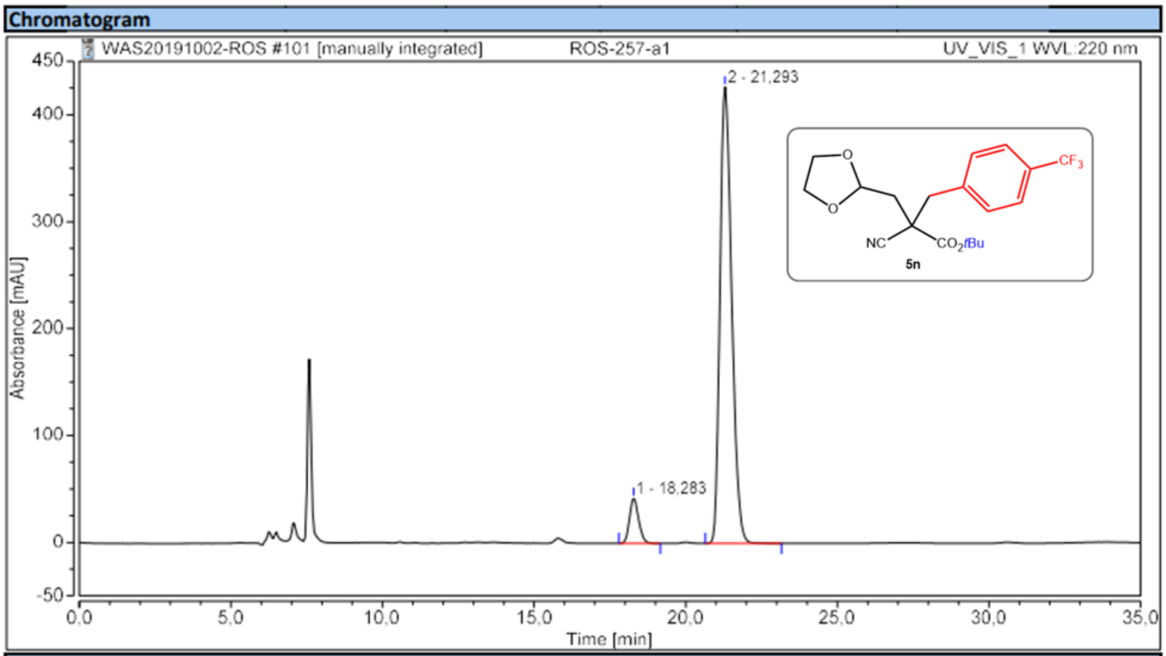

Integration Results

No.	Peak Name	Retention Time min	Area mAU*min	Height mAU	Relative Area %	Relative Height %	Amount n.a.
1		18,283	14,700	42,050	7,16	8,97	n.a.
2		21,293	190,650	426,810	92,84	91,03	n.a.
Total:			205,350	468,860	100,00	100,00	

Chromatogram of racemic product **5o**

Chromatogram

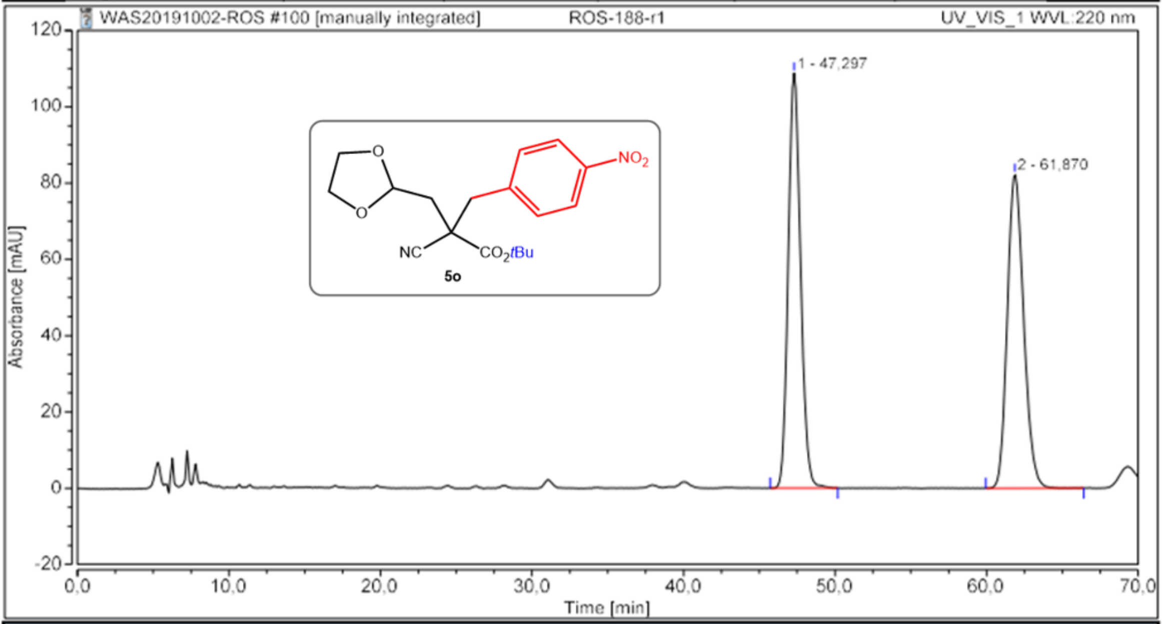

Integration Results

No.	Peak Name	Retention Time min	Area mAU*min	Height mAU	Relative Area %	Relative Height %	Amount n.a.
1		47,297	102,809	108,831	49,98	56,93	n.a.
2		61,870	102,881	82,320	50,02	43,07	n.a.
**Total:**			**205,691**	**191,151**	**100,00**	**100,00**	

Chromatogram of enantioenriched product **5o**

Chromatogram

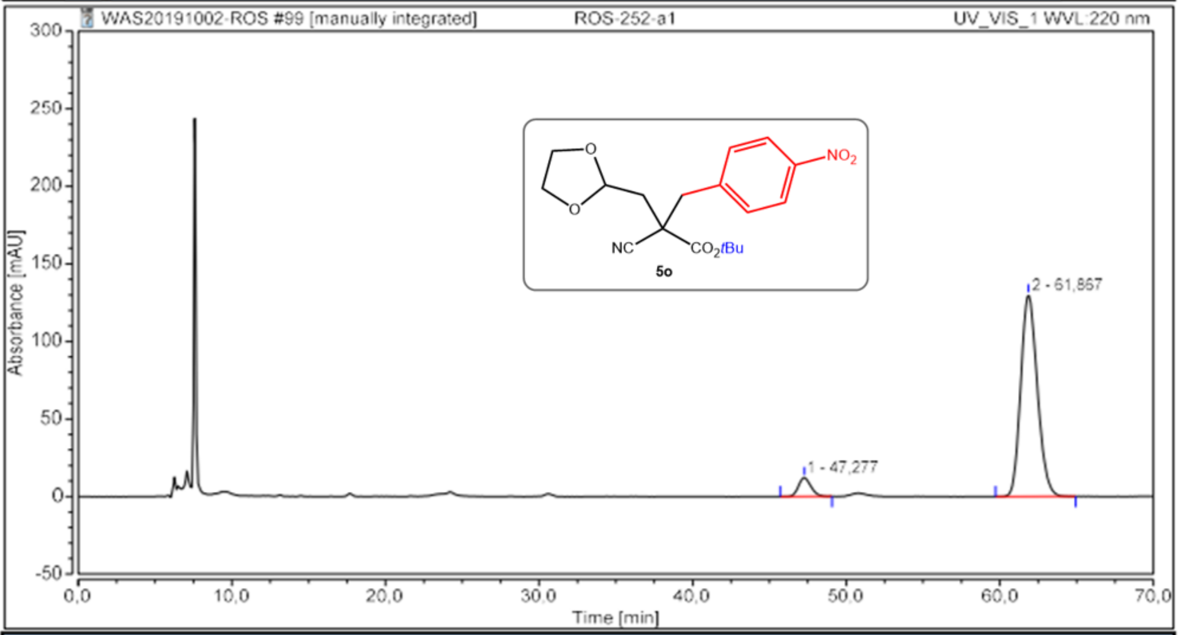

Integration Results

No.	Peak Name	Retention Time min	Area mAU*min	Height mAU	Relative Area %	Relative Height %	Amount n.a.
1		47,277	11,401	12,192	6,55	8,57	n.a.
2		61,867	162,697	130,022	93,45	91,43	n.a.
**Total:**			**174,098**	**142,214**	**100,00**	**100,00**	

Chromatogram of racemic product **5p**

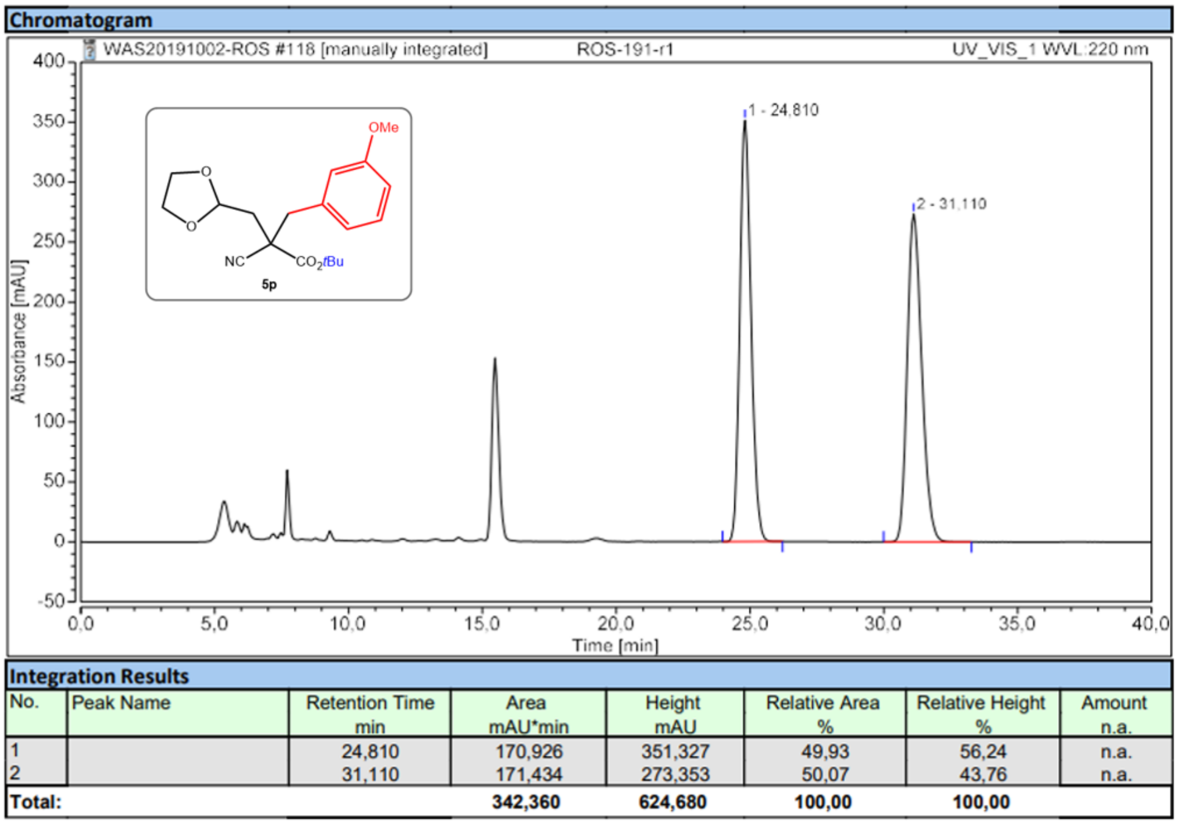

Integration Results

No.	Peak Name	Retention Time min	Area mAU*min	Height mAU	Relative Area %	Relative Height %	Amount n.a.
1		24,810	170,926	351,327	49,93	56,24	n.a.
2		31,110	171,434	273,353	50,07	43,76	n.a.
**Total:**			**342,360**	**624,680**	**100,00**	**100,00**	

Chromatogram of enantioenriched product **5p**

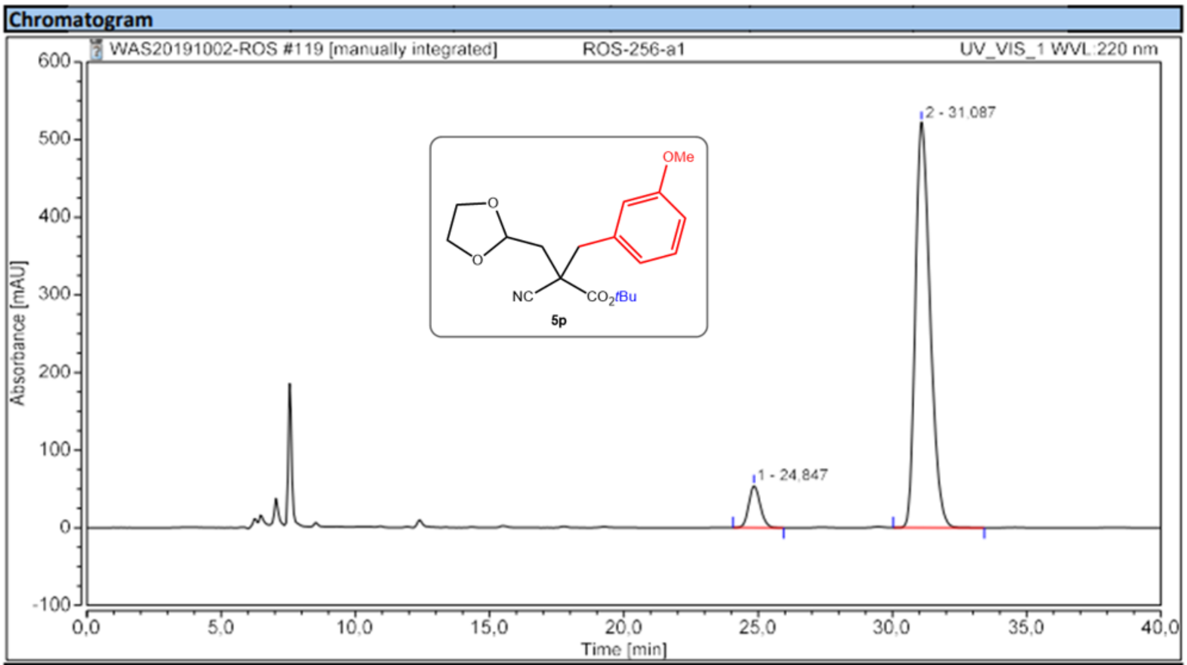

Integration Results

No.	Peak Name	Retention Time min	Area mAU*min	Height mAU	Relative Area %	Relative Height %	Amount n.a.
1		24,847	26,639	54,192	7,41	9,40	n.a.
2		31,087	332,647	522,440	92,59	90,60	n.a.
**Total:**			**359,286**	**576,632**	**100,00**	**100,00**	

Chromatogram of racemic product **5q**

Chromatogram

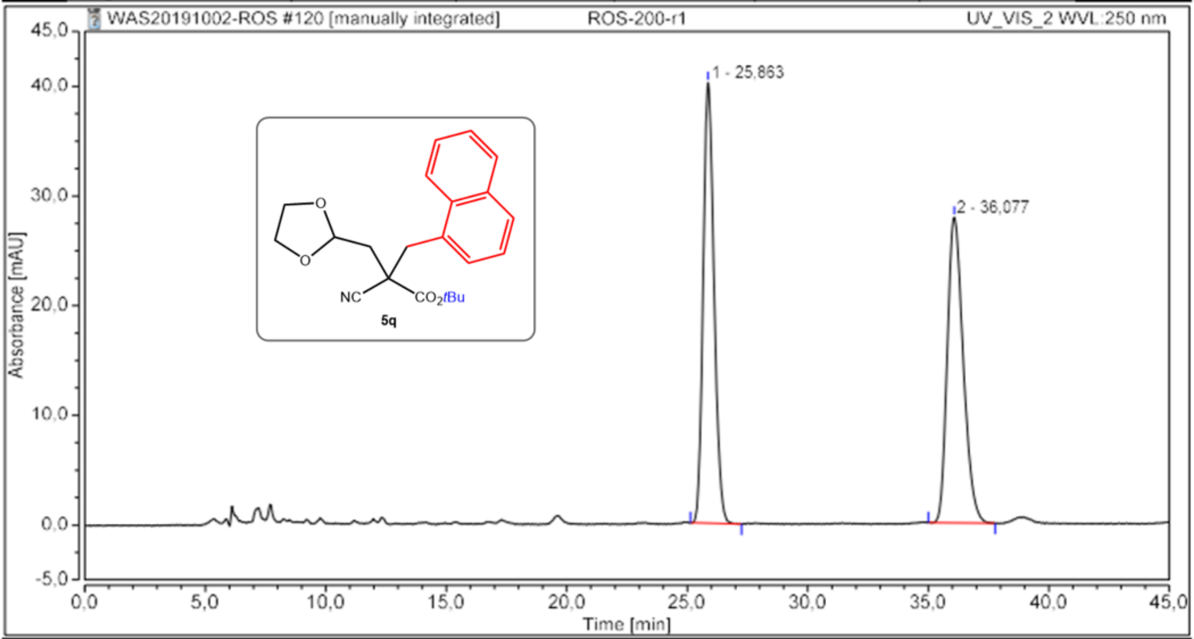

Integration Results

No.	Peak Name	Retention Time min	Area mAU*min	Height mAU	Relative Area %	Relative Height %	Amount n.a.
1		25,863	21,032	40,175	49,65	59,04	n.a.
2		36,077	21,325	27,875	50,35	40,96	n.a.
**Total:**			**42,357**	**68,050**	**100,00**	**100,00**	

Chromatogram of enantioenriched product **5q**

Chromatogram

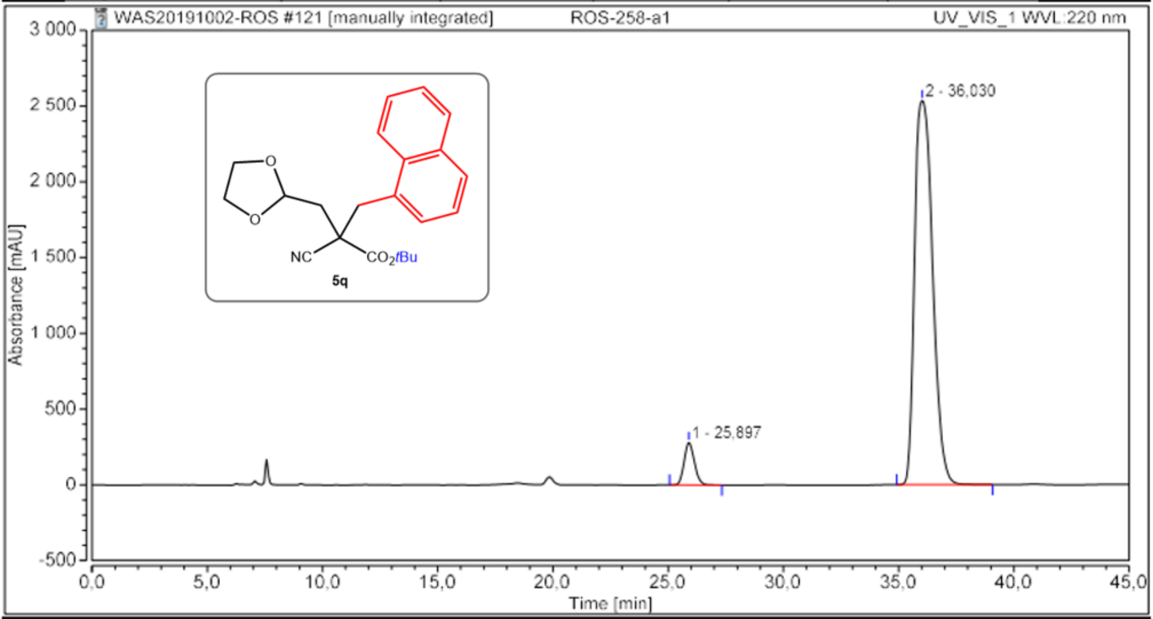

Integration Results

No.	Peak Name	Retention Time min	Area mAU*min	Height mAU	Relative Area %	Relative Height %	Amount n.a.
1		25,897	144,969	281,779	5,93	10,00	n.a.
2		36,030	2300,388	2535,800	94,07	90,00	n.a.
**Total:**			**2445,357**	**2817,578**	**100,00**	**100,00**	
